# Palladium-catalyzed/copper-mediated carbon–carbon cross-coupling reaction for synthesis of 6-unsubstituted 2-aryldihydropyrimidines†

**DOI:** 10.1039/d2ra05155a

**Published:** 2022-10-03

**Authors:** Yoshio Nishimura, Takanori Kubo, Saho Takayama, Hanako Yoshida, Hidetsura Cho

**Affiliations:** School of Pharmaceutical Sciences, Ohu University 31-1 Misumido, Tomita-machi Koriyama Fukushima 963-8611 Japan y-nishimura@pha.ohu-u.ac.jp; Faculty of Pharmacy, Yasuda Women's University 6-13-1, Yasuhigashi, Asaminami-ku Hiroshima 731-0153 Japan; Graduate School of Pharmaceutical Sciences, Tohoku University 6-3 Aoba, Aramaki, Aoba-ku Sendai 980-8578 Japan

## Abstract

Dihydropyrimidines (DPs) show a wide range of biological activities for medicinal applications. Among the DP derivatives, 2-aryl-DPs have been reported to display remarkable pharmacological properties. In this work, we describe a method for the synthesis of hitherto unavailable 6-unsubstituted 2-aryl-DPs by Pd-catalyzed/Cu-mediated carbon–carbon cross-coupling reaction of 1-Boc 2-methylthio-DPs with organostannane reagents. The Boc group of the substrate significantly increases the substrate reactivity. Aryl tributylstannanes having various substituents such as MeO, Ph, CF_3_, CO_2_Me, and NO_2_ groups smoothly afforded the corresponding products in high yields. Various heteroaryl tributylstannanes having 2-, or 3-thienyl, 2-, or 3-pyridinyl groups were also applicable to the reaction. Regarding the substituents at the 4-position, the reactions of DPs bearing various aryl and alkyl substituents proceeded smoothly to give the desired products. The Boc group of the products was removed under a standard acidic condition to produce *N*-unsubstituted DP as a mixture of the tautomers in quantitative yields. The synthetic procedure was also applied to 4,4,6-trisubstituted 2-methylthio-DP to give novel 2,4,4,5,6-pentasubstituted DP. Therefore, the Pd-catalyzed/Cu-mediated reaction should help expand the DP-based molecular diversity, which would impact biological and pharmacological studies.

## Abstract 2

This protocol enables the synthesis of 6-unsubstituted 2-aryldihydropyrimidines using various substituents at the 2- and 4-positions, which would impact dihydropyrimidine-based biological and pharmacological studies.

## Graphical abstract

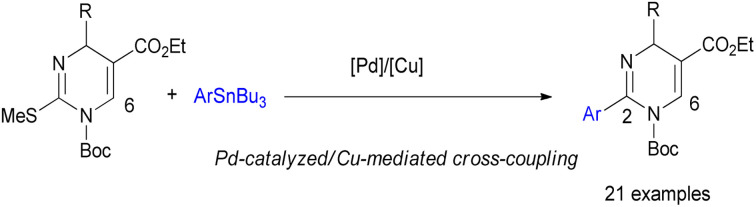

## Introduction

**Fig. 1:**
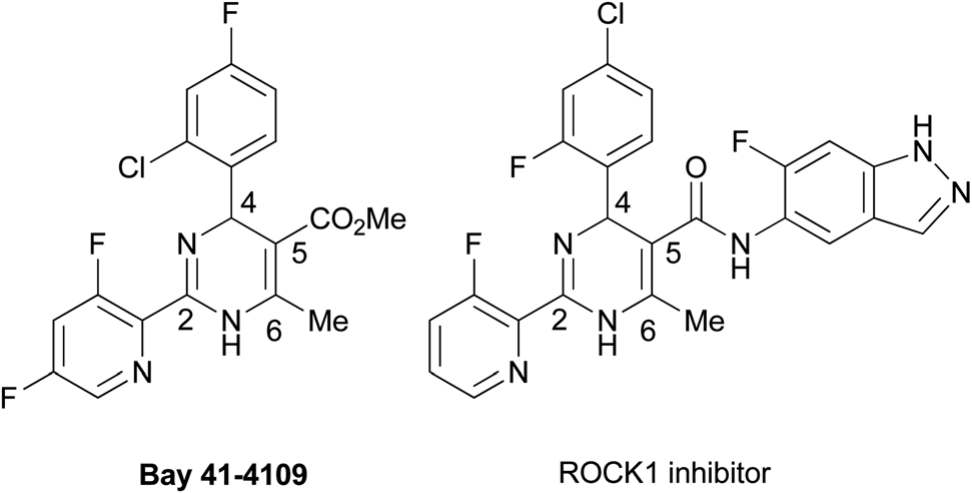
Biologically active 2-aryl-DPs.

Dihydropyrimidines (DPs) show a wide range of biological activities for medicinal applications. They display calcium channel inhibitory,^1^ anticancer,^2^ antibacterial,^3^ antifungal,^4^ anti-HIV,^5^ antimalarial,^6^ anti-inflammatory,^7^ and antioxidation^8^ activities. Many reviews on synthetic methods developed for the heterocycles and their biological activities published thus far suggest their great potential as leading compounds for developing medicines.^9^ Among the DP derivatives, tautomeric 2-aryl-DPs have been reported to display remarkable pharmacological properties (Fig. 1). In 2003, Bay 41-4109 was shown to exhibit highly potent anti-hepatitis B virus (HBV) replication activity *in vitro* and *in vivo*.^10^ As a Bay 41-4109 analog with good water solubility, 6-morpholinylmethyl DP hydrochloride salt was reported as a HBV capsid assembly inhibitor.^11^ In 2008, another tautomeric 2-aryl-DP was also developed as a Rho-associated kinase isoform 1 (ROCK1) inhibitor, which may be a potential therapeutic agent for cardiovascular diseases.^12^ Recently 2-arylethenyl DP was reported as a potent heat shock protein 90 (Hsp90) C-terminal inhibitor, which may be a drug candidate for cancer therapeutics.^13^

**Scheme 1:**
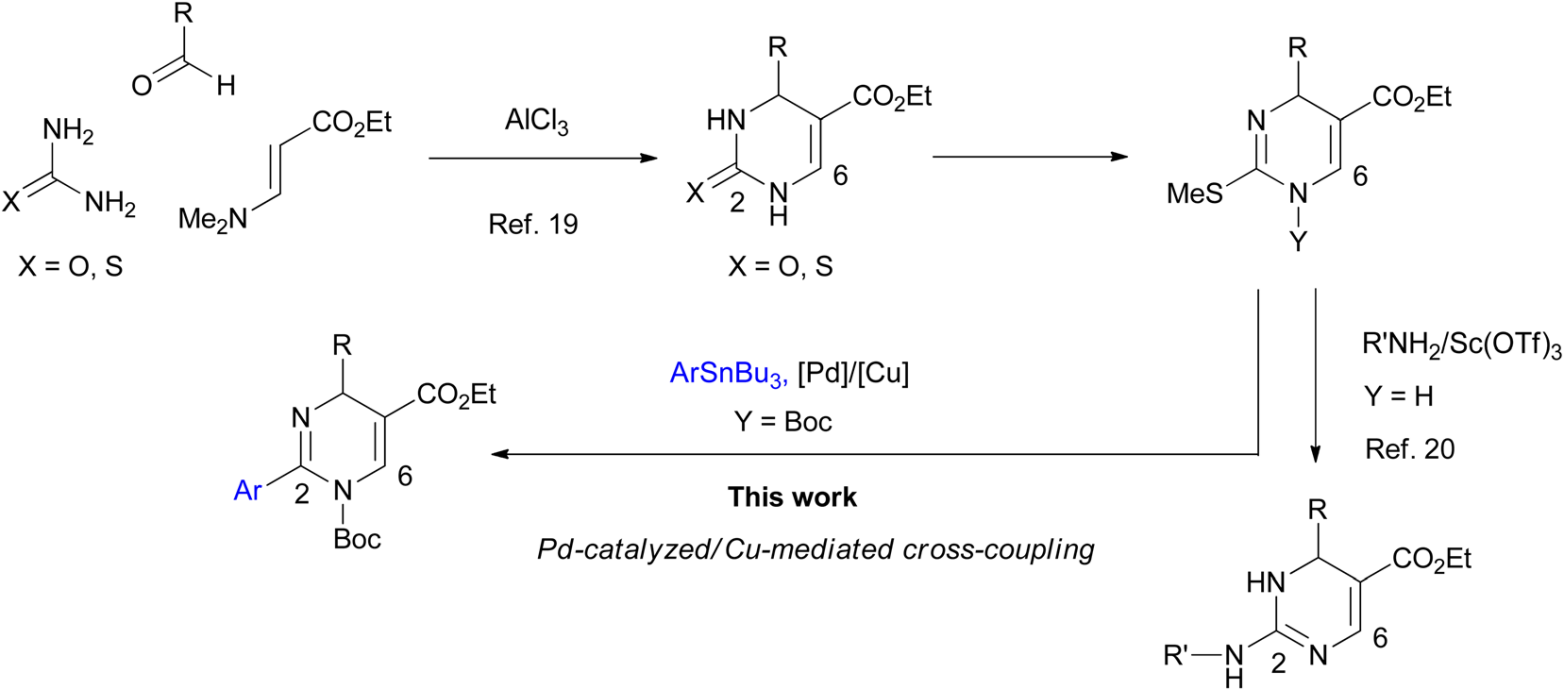
Synthesis of a series of 6-unsubstituted DPs.

The biologically important tautomeric 2-aryl-DPs shown in Fig. 1 have four substituents at the 2-, 4-, 5-, and 6-positions. In general, these derivatives and related compounds were synthesized by three-component cyclocondensation reaction such as Biginelli reaction,^9–12^ or a transition-metal-catalyzed arylation reaction from 2-thioxo-DPs prepared in advance.^13,14^ Recently a one-pot synthetic method for tetrasubstituted 2-aryl-DPs from α-azidocinnamates by irradiation of LED light and base-catalyzed isomerization was also reported.^15^ Development of synthetic methods to access tautomeric 2-aryl-DPs with different substituent patterns expands their structural diversity, which impacts the DP-based drug discovery program. For example, a conventional cyclocondensation reaction of arylamidine with α,β-unsaturated aldehydes gives simple 2-aryl-DPs with fewer substituents.^16^ We previously reported the cyclization–elimination sequential reactions of 1,3-diazabuta-1,3-diene with electron-deficient olefins to give hitherto unavailable 4,6-unsubstituted 2-phenyl-DPs and related analogs.^17^ With our continuing interest in developing efficient methods of synthesizing DPs with fewer or more substituents,^18^ we have recently developed a general synthetic method for 6-unsubstituted DPs (Scheme 1). The 2-oxo- and 2-thioxo-DPs were synthesized by an AlCl_3_-mediated Biginelli-type three-component cyclocondensation reaction involving urea, aldehyde, and aminoacrylate.^19^ The 2-thioxo-DPs were stepwise converted into hitherto unavailable 2-amino-DPs *via* Sc(OTf)_3_-mediated nucleophilic substitution of 2-methylthio-DPs with amines.^20^ The proliferative effect of these 6-unsubstituted 2-oxo-, 2-thioxo-, and 2-amino-DPs on the human promyelocytic leukemia cell line HL-60 was also accessed, which led to the discovery of a highly active 2-benzylamino-DP with IC_50_ of <100 nM.^20^ In this study, we planned that 2-methylthio-DPs or 2-thioxo-DPs were used as precursors for the synthesis of hitherto unavailable 6-unsubstituted 2-aryl-DPs by a transition-metal-catalyzed 2-arylation reaction, Liebeskind–Srogl-type cross-coupling reaction.^21^ As a result, we realized the Pd-catalyzed/Cu-mediated 2-arylation reaction of 1-Boc 2-methylthio-DPs with arylstannane reagents.^22^ The Boc group significantly increases reactivity of DPs. This protocol enables the synthesis of 6-unsubstituted 2-aryl-DPs using various substituents at the 2- and 4-positions; to the best of our knowledge, the general formula of the 2-aryl-DPs has not been reported. Owing to our results, a series of 6-unsubstituted 2-oxo-, 2-thioxo-, and 2-amino-, and 2-aryl-DPs becomes available, which would impact DP-based biological and pharmacological studies.

## Results and discussion

**Scheme 2:**
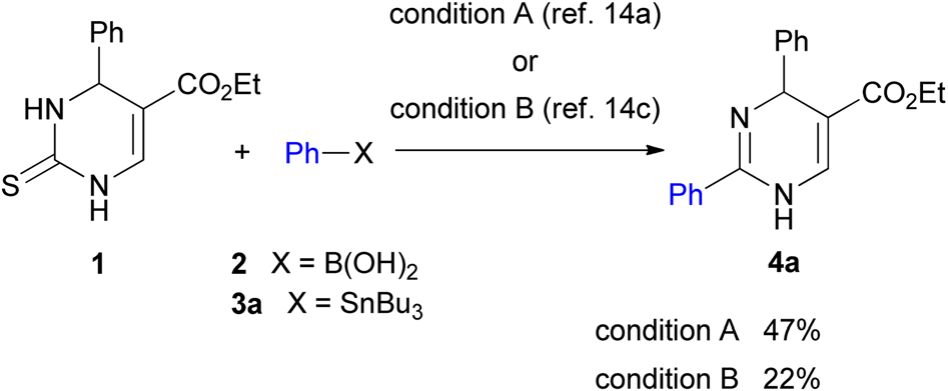
Reactions of 2-thioxo-DP 1 under reported reaction conditions.

Initial studies of 2-thioxo-DP 1 were carried out under reaction condition A reported by Kappe^14*a*^ [Pd(PPh_3_)_4_ (3.0 mol%), Cu(i)-thiophene-2-carboxylate (CuTC, 3.0 equiv.), PhB(OH)_2_2 (1.5 equiv.) in THF at reflux for 18 h] and condition B reported by Suzenet^14*c*^ [Pd(PPh_3_)_4_ (5.0 mol%), CuBr·Me_2_S (2.2 equiv.), PhSnBu_3_3a (2.2 equiv.) in THF at reflux for 24 h]. These reactions gave 2-phenyl-DP 4a in moderate yields of 47% under condition A and 22% under condition B (Scheme 2).

**Scheme 3:**
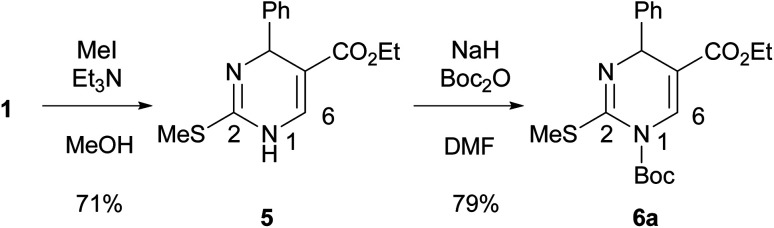
Preparation of DP 5 and 6a from 1.

To increase the yield of the 2-arylation product, DP 1 was converted into 2-methylthio-DP 5 because the methylthio group is a typical substrate for the Liebeskind–Srogl reaction (Scheme 3).^21^ Our previous studies on the substitution reaction of DPs showed that a Boc group increased the electrophilicity of DPs.^23^ Therefore, 1-Boc 2-methylthio DP 6a was prepared by incorporating the Boc group into 5. The reaction occurred preferentially at the 1-position of 5 to give 6a in 79% yield. The position of the Boc group of 6a was determined; as for 1-Boc 2-phenyl DP 7a shown in Table 1, a significant heteronuclear multiple bond correlation (HMBC) was observed between the 6-proton and the carbonyl carbon of the Boc group at the 1-position. Therefore, the Boc groups of 7a and 6a were determined to be located at the 1-position. To determine a suitable substrate for the cross-coupling reaction, the reactivity of 6a was examined and compared with those of 1 and 5.

**Table 1:** Optimization of reaction conditions^a^

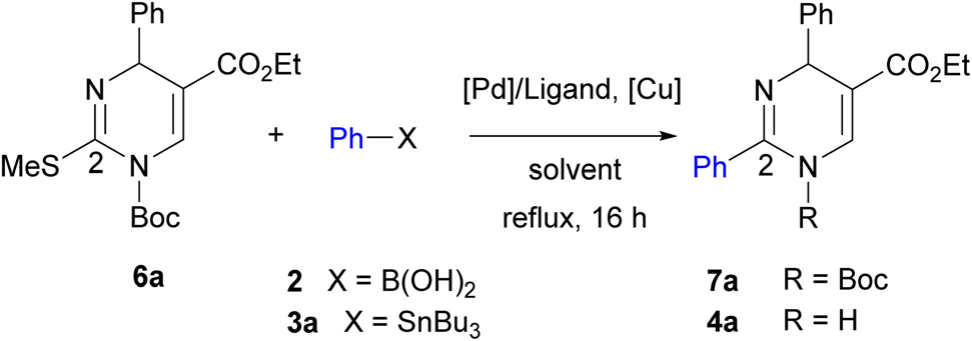					
Entry	DP/arylating reagent^a^	[Pd]/ligand/[Cu]^a^	Solvent/temp./time	Combined yield (%) (7a + 4a)	Recovery (%) of DP
1	6a/3a	Pd(PPh_3_)_4_/none/CuTC	THF/reflux/16 h	65 (55 + 10)	8
2	6a/3a	Pd(PPh_3_)_4_/none/CuBr·Me_2_S	THF/reflux/16 h	58 (16 + 42)	35
3	6a/3a	PdCl_2_(PPh_3_)_2_/none/CuTC	THF/reflux/16 h	74 (64 + 10)	13
4	6a/3a	Pd(OAc)_2_/none/CuTC	THF/reflux/16 h	54 (49 + 5)	33
5	6a/3a	Pd_2_dba_3_/(2-furyl)_3_P/CuTC	THF/reflux/16 h	80 (70 + 10)	7
6	6a/2	Pd_2_dba_3_/(2-furyl)_3_P/CuTC	THF/reflux/16 h	65 (56 + 9)	24
7	6a/3a	Pd_2_dba_3_/none/CuTC	THF/reflux/16 h	55 (45 + 10)	43
8	6a/3a	Pd_2_dba_3_/Ph_3_P/CuTC	THF/reflux/16 h	63 (58 + 5)	27
9	6a/3a	Pd_2_dba_3_/(2-thienyl)_3_P/CuTC	THF/reflux/16 h	63 (56 + 7)	31
10	6a/3a	Pd_2_dba_3_/(2-MeOC_6_H_4_)_3_P/CuTC	THF/reflux/16 h	16 (16 + 0)	76
11	6a/3a	Pd_2_dba_3_/(*cyclo*-C_6_H_11_)_3_P/CuTC	THF/reflux/16 h	11 (11 + 0)	86
12	6a/3a	Pd_2_dba_3_/dppm/CuTC	THF/reflux/16 h	21 (21 + 0)	68
13	6a/3a	Pd_2_dba_3_/dppe/CuTC	THF/reflux/16 h	17 (17 + 0)	70
14	6a/3a	Pd_2_dba_3_/dppp/CuTC	THF/reflux/16 h	22 (22 + 0)	65
15	6a/3a	Pd_2_dba_3_/dppb/CuTC	THF/reflux/16 h	49 (41 + 8)	48
16	6a/3a	Pd_2_dba_3_/dppf/CuTC	THF/reflux/16 h	51 (44 + 7)	44
17	6a/3a	Pd_2_dba_3_/*rac*-BINAP/CuTC	THF/reflux/16 h	26 (26 + 0)	58
18	6a/3a	None/none/CuTC	THF/reflux/16 h	3 (3 + 0)	95
19	6a/3a	Pd_2_dba_3_/(2-furyl)_3_P/none	THF/reflux/16 h	0	96
20	6a/3a	Pd_2_dba_3_/(2-furyl)_3_P/CuTC	Dioxane/70 °C/16 h	74 (66 + 8)	22
21	6a/3a	Pd_2_dba_3_/(2-furyl)_3_P/CuTC	DMF/70 °C/16 h	78 (62 + 16)	18
22	6a/3a	Pd_2_dba_3_/(2-furyl)_3_P/CuTC	Toluene/70 °C/16 h	66 (63 + 3)	27
23	6a/3a	Pd_2_dba_3_/(2-furyl)_3_P/CuTC	1,2-DCE/70 °C/16 h	78 (72 + 6)	20
24	6a/3a	Pd_2_dba_3_/(2-furyl)_3_P/CuTC	CH_2_Cl_2_/reflux/16 h	81 (79 + 2)	18
25	6a/3a	Pd_2_dba_3_/(2-furyl)_3_P/CuTC	CH_2_Cl_2_/reflux/30 h	93 (91 + 2)	2
26	1/3a	Pd_2_dba_3_/(2-furyl)_3_P/CuTC	CH_2_Cl_2_/reflux/30 h	24 (only 4a)	0
27	5/3a	Pd_2_dba_3_/(2-furyl)_3_P/CuTC	CH_2_Cl_2_/reflux/30 h	55 (only 4a)	15
28^b^	6a/3a	Pd_2_dba_3_/(2-furyl)_3_P/CuTC	CH_2_Cl_2_/reflux/30 h	82 (80 + 2)	10

a Reaction conditions: 6a (0.25 mmol), 3a (0.50 mmol), Pd catalyst (5.0 mol%), ligand (20 mol%), and Cu reagent (0.50 mmol) in solvent (3 mL) were reacted under Ar.

b Pd_2_dba_3_ (1.0 mol%) and (2-furyl)_3_P (8.0 mol%) were used.

The optimized reaction conditions for 6a are summarized in Table 1. The effect of two Cu sources was examined under the same reaction condition, and results showed that CuTC worked better than CuBr·Me_2_S to give a combined yield of 65% for a desired 2-phenyl-DP 7a and 4a (entries 1 and 2). In all reactions using 3 in this study, the DPs 7a and 4a were purified by column chromatography using silica gel–K_2_CO_3_ (10 : 1) to prevent mixing with degradation product from 3.^24^ Among the Pd catalysts tested, tris(dibenzylideneacetone)dipalladium (Pd_2_dba_3_) with (2-furyl)_3_P used in the reaction gave a good combined yield of 80% for 7a and 4a (entries 1, 3–5). As an arylation reagent, PhSnBu_3_3a showed a higher reactivity than PhB(OH)_2_2 (entries 5 and 6). Subsequently, the effect of phosphine ligands was examined; only a few monodentate ligands, such as (2-furyl)_3_P, (2-thienyl)_3_P, and triphenylphosphine (Ph_3_P), increased the yields compared with the reaction without phosphine (entries 5, 7–9). The reactions using other monodentate ligands such as (2-MeOC_6_H_4_)_3_P and (*cyclo*-C_6_H_11_)_3_P resulted in low yields (entries 10 and 11). All bidentate ligands including 1,1-bis(diphenylphophino)methane (dppm), 1,2-bis(diphenylphophino)ethane (dppe), 1,3-bis(diphenylphophino)propane (dppp), 1,1-bis(diphenylphophino)butane (dppb), 1,1′-bis(diphenylphophino)ferrocene (dppf), and racemic BINAP (*rac*-BINAP) gave low yields (entries 12–17). As a result, the best ligand was determined to be (2-furyl)_3_P (entry 5). We confirmed that either reaction in the absence of Pd_2_dba_3_/(2-furyl)_3_P or CuTC hardly proceeded with the recovery of only 6a (entries 18 and 19); therefore, the addition of these reagents was essential for the reaction. To examine the effect of solvents, several polar and nonpolar solvents, such as dioxane (1,4-dioxane), DMF, toluene, 1,2-DCE (1,2-dichloroethane), and CH_2_Cl_2_, were used (entries 20–24). Although a small effect on the yields was observed, the reaction in CH_2_Cl_2_ showed a superior result and good mass balance to give a combined yield of 81% for 7a and 4a with 18% recovery of 6a (entry 24). When the reaction was conducted for a longer time of 30 h, the combined yield of 7a and 4a was increased to 93% (entry 25). When the optimized reaction condition was applied to the reactions using 1 or 5 as a substrate, the desired 4a was obtained in lower yields of 24% and 55%, respectively (entries 26 and 27). Therefore, the best substrate among 1, 5, and 6a for the reaction was determined to be 6a. The Boc group in 6a had a significant effect on the reactivity of 6a probably owing to its high electrophilicity being further increased by the group. When lower amount of Pd_2_dba_3_ (1 mol%) and (2-furyl)_3_P (8 mol%) were used, the combined yield slightly decreased to 82% (entry 28).

**Scheme 4:**
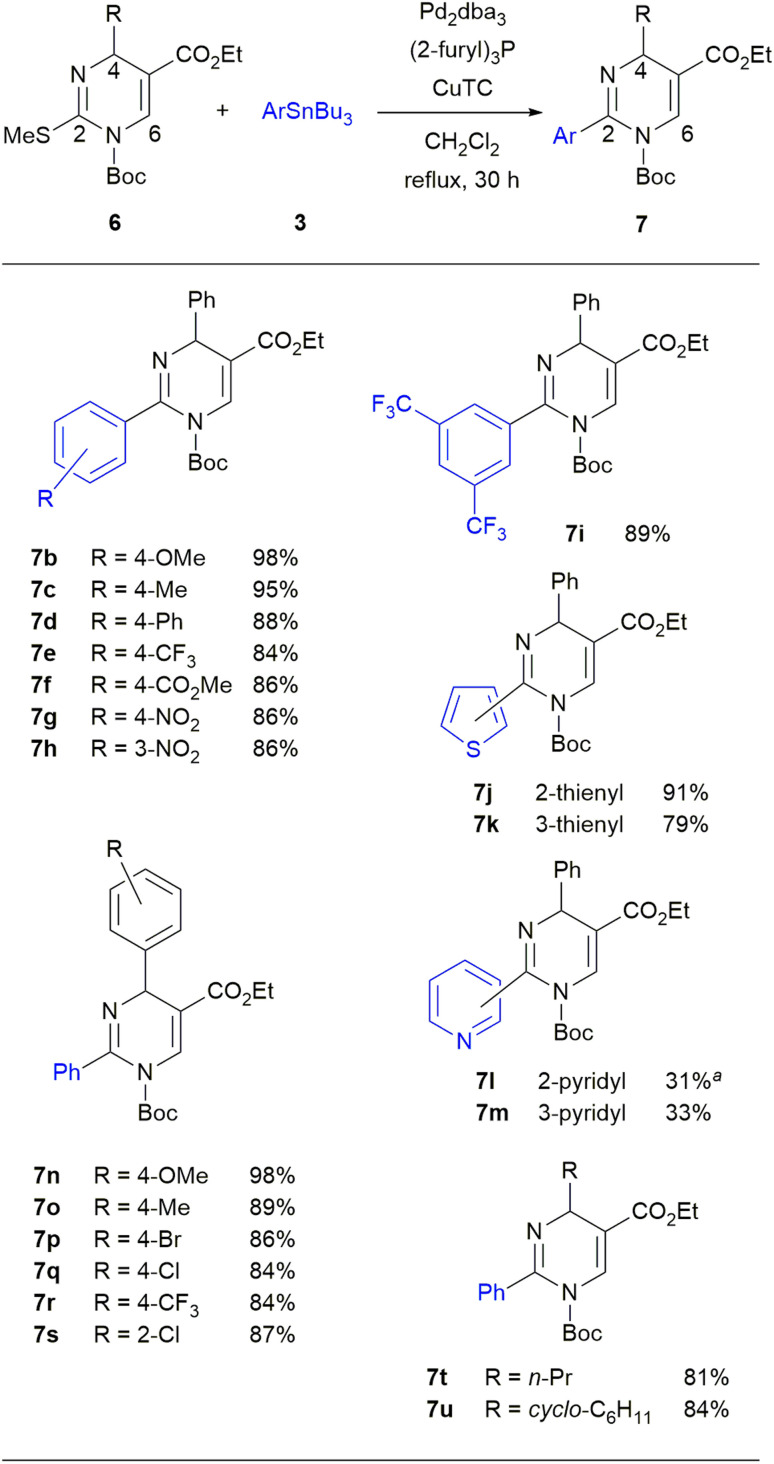
Synthesis of 6-unsubstituted 2-aryl-DPs 7. Reaction conditions: 6 (0.25 mmol), 3 (0.50 mmol, 2.0 equiv.), Pd_2_dba_3_ (2.5 mol%), (2-furyl)_3_P (20 mol%), CuTC (0.50 mmol, 2.0 equiv.), and CH_2_Cl_2_ (3 mL) at reflux for 30 h under Ar. ^*a*^3 (4.0 equiv.), Pd_2_dba_3_ (5.0 mol%), (2-furyl)_3_P (40 mol%), CuTC (4.0 equiv.) were used.

With the optimized condition in hand, we examined the scope of the Pd-catalyzed/Cu-mediated reaction using diverse aryl tributylstannanes 3 and DP derivatives 6 (Scheme 4). Regarding 3, we found no clear preference for either electron-donating or electron-withdrawing substituents of the phenyl group. When 6a (R = Ph) was reacted with *p*-methoxyphenyl- or *p*-tolyl tributylstannanes, the desired DPs 7b and 7c were produced in high yields of 98% and 95%, respectively. Aryl tributylstannanes having other substituents such as Ph, CF_3_, CO_2_Me, and NO_2_ groups at the *para* position smoothly afforded to give the products 7d–7g in 84–88% yields. The reactions using *m*-nitrophenyl or 3,5-bis(trifluoromethyl)phenyl tributylstannanes also proceeded smoothly to afford the products 7h and 7i in 86% and 89% yields, respectively. Various heteroaryl tributylstannanes having 2-thienyl, 3-thienyl, 2-pyridinyl, and 3-pyridinyl groups also reacted with 6a to give 7j–7m, albeit with low yields of 31–33% in the case of pyridine. We next examined the reaction scope for 6 using different substituents at the 4-position. We prepared seven 4-aryl-DPs 6a–6g having substituents such as H, OMe, Me, Br, Cl, and CF_3_ groups at the *para* position and Cl group at the *ortho* position. 4-*n*-Propyl-DP 6h and 4-cyclohexyl-DP 6i were also prepared. The synthetic procedure and the characteristic data of these DPs 6a–6i were shown in the experimental section. Regarding the aryl group of 6 at the 4-position, the reactions of DPs bearing substituents at the *para* position, proceeded smoothly to give the desired products 7n–7r in 84–98% yields. The reaction of the DP with the *ortho*-chlorophenyl group at the 4-position gave a DP 7s in 87% yield. Alkyl substituents such as *n*-propyl and cyclohexyl groups were also tolerated in the reaction to afford 7t and 7u in good yields.

**Scheme 5:**
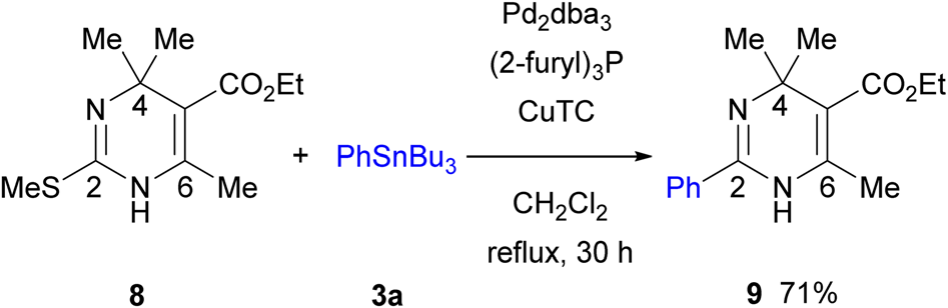
Synthesis of 2,4,4,5,6-pentasubstituted DP 9.

The Pd-catalyzed/Cu-mediated reaction was applied to 4,4,6-trisubstituted 2-methylthio-DP 8 (Scheme 5).^18*a*^ An attempt to incorporate a Boc group to *N*-unsubstituted 8 using NaH/Boc_2_O failed owing to the steric congestion around the nitrogen atom. However, the reaction of 8 under the optimized conditions in Table 1 proceeded smoothly to give 2,4,4,5,6-pentasubstituted DP 9 in 71% yield. Such fully substituted 2-aryl-DP 9 has not been found in literature. Further optimization of the reaction condition for the synthesis of related pentasubstituted DPs is in progress.

**Scheme 6:**
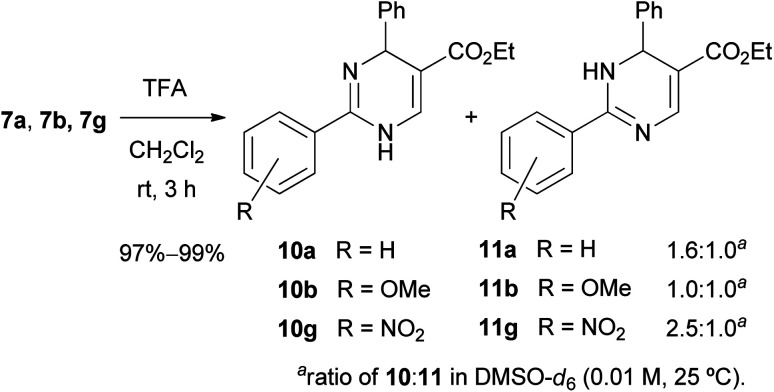
Synthesis and analysis of 2-aryl-DP tautomers.

The Boc group of 7 was removed under a standard acidic condition (TFA in CH_2_Cl_2_) to produce *N*-unsubstituted 1,4-DP 10 and 1,6-DP 11 as a mixture of the tautomers (Scheme 6). To analyze the tautomeric behavior of 10 and 11, ^1^H NMR spectra of a mixture of 10a/11a, 10b/11b, and 10g/11g were measured in CD_3_OD and DMSO-*d*_6_, respectively (0.01 M, 25 °C). In CD_3_OD, only average spectra of 10/11 were observed because of the relatively fast tautomerization in the protic solvent. On the other hand, two individual tautomers of 10/11 were observed in the ratio of 1.0 : 1.0–2.5 : 1.0 in DMSO-*d*_6_. The ratio of 10/11 in DMSO-*d*_6_ was affected by substituents at the *para* position of the 2-phenyl group; the ratios were 1.0 : 1.0 for 10b/11b (R = OMe), 1.6 : 1.0 for 10a/11a (R = H), and 2.5 : 1.0 for 10g/11g (R = NO_2_). These results indicate that the electron-donating property of the MeO group stabilized 1,6-DP 11b and increased the ratio of 11b owing to the resonance effect from the MeO group to the carbonyl group at the 5-position. In contrast, the electron-withdrawing property of the NO_2_ group weakens the effect and destabilizes 1,6-DP 11g. The thermodynamic preference of 1,4-DPs such as 10a and 10g over 11a and 11g was supported by our previous experimental and theoretical studies on 2-substituted DP tautomers.^25^

In summary, we have developed a Pd-catalyzed/Cu-mediated cross-coupling reaction for the synthesis of 6-unsubstituted 2-aryl-DPs 7 from 1-Boc 2-methylthio-DP 6. The incorporation of the Boc group at the nitrogen atom of 6 significantly increased the reactivity of 6. The method is compatible with diverse DP substrates and aryl tributylstannane reagents. The method is also applicable to the reaction using 8 for the synthesis of highly pentasubstituted 2-aryl-DP 9. The Boc group of 7 was removed quantitatively to obtain a tautomeric mixture of 10/11. The synthetic procedure should help expand the DP-based molecular diversity, which would impact biological and pharmacological studies.

## Experimental section

### General information

Melting points were determined with an AS ONE melting point apparatus ATM-02 (AS ONE Corporation, Japan) or Yanaco melting point apparatus MP-J3 without correction. ^1^H NMR spectra were recorded on a Bruker AVANCE™ III 600 (600 MHz, Bruker Japan K.K., Japan) or JEOL JNM-ECZ500R (500 MHz, JEOL Ltd., Japan) with tetramethylsilane (*δ* 0 ppm) in CDCl_3_ or dimethylsulfoxide (*δ* 2.49 ppm) in DMSO-*d*_6_, or methanol (*δ* 3.30 ppm) in CD_3_OD as internal standards. ^13^C NMR spectra were recorded on a Bruker AVANCE™ III 600 (150 MHz) or JEOL JNM-ECZ500R (125 MHz) with chloroform (*δ* 77.0 ppm) in CDCl_3_ or dimethylsulfoxide (*δ* 39.7 ppm) in DMSO-*d*_6_ or methanol (*δ* 49.0 ppm) in CD_3_OD as internal standards. Multiplicities for ^1^H NMR were designated as s = singlet, d = doublet, t = triplet, q = quartet, dd = doublet of doublets, dt = doublet of triplets, dq = doublet of quartets, tt = triplet of triplets, ddd = doublet of doublets of doublets, m = multiplet, and br = broad. Infrared spectra (IR) were measured on a JASCO FT/IR-6100 or JASCO FT/IR-4100 Fourier transform infrared spectrophotometer (JASCO Corporation, Japan). Mass spectra were recorded on a JEOL JMS-700 mass analyzer (JEOL Ltd., Japan). High-resolution spectroscopy (HRMS) was performed using a JEOL JMS-700 mass analyzer.

### Synthesis of starting materials 6

Following the literature procedure,^19^ 1-*tert*-butyl 5-ethyl 2-methylthio-4-phenyl-1,4-dihydropyrimidine-1,5-dicarboxylate (6a),^19^ 1-*tert*-butyl 5-ethyl 4-(4-methoxyphenyl)-2-methylthio-1,4-dihydropyrimidine-1,5-dicarboxylate (6b), 1-*tert*-butyl 5-ethyl 4-(4-methylphenyl)-2-methylthio-1,4-dihydropyrimidine-1,5-dicarboxylate (6c), 1-*tert*-butyl 5-ethyl 4-(4-bromophenyl)-2-methylthio-1,4-dihydropyrimidine-1,5-dicarboxylate (6d), 1-*tert*-butyl 5-ethyl 4-(4-chlorophenyl)-2-methylthio-1,4-dihydropyrimidine-1,5-dicarboxylate (6e), 1-*tert*-butyl 5-ethyl 4-[4-(trifluoromethyl)phenyl]-2-methylthio-1,4-dihydropyrimidine-1,5-dicarboxylate (6f), 1-*tert*-butyl 5-ethyl 4-(2-chlorophenyl)-2-methylthio-1,4-dihydropyrimidine-1,5-dicarboxylate (6g), 1-*tert*-butyl 5-ethyl 2-methylthio-4-propyl-1,4-dihydropyrimidine-1,5(4*H*)-dicarboxylate (6h),^19^ 1-*tert*-butyl 5-ethyl 4-cyclohexyl-2-methylthio-1,4-dihydropyrimidine-1,5-dicarboxylate (6i) were prepared.

#### 1-*tert*-Butyl 5-ethyl 4-(4-methoxyphenyl)-2-methylthio-1,4-dihydropyrimidine-1,5-dicarboxylate (6b)

Pale yellow oil. ^1^H NMR (CDCl_3_, 600 MHz): *δ* = 1.23 (t, *J* = 7.2 Hz, 3H), 1.59 (s, 9H), 2.28 (s, 3H), 3.78 (s, 3H), 4.14 (dq, *J* = 10.8, 7.2 Hz, 1H), 4.17 (dq, *J* = 10.8, 7.2 Hz, 1H), 5.67 (s, 1H), 6.84 (d, *J* = 8.4 Hz, 2H), 7.22 (d, *J* = 8.4 Hz, 2H), 7.93 (s, 1H). ^13^C NMR (CDCl_3_, 150 MHz): *δ* = 14.1, 15.9, 28.0, 55.2, 58.3, 60.5, 85.7, 111.9, 113.8, 128.3, 132.3, 134.7, 148.4, 149.2, 158.9, 165.3. IR (neat): 2981, 1741, 1711, 1669, 1607, 1510, 1335, 1250, 1155, 1082, 1044 cm^−1^. HRMS-FAB: *m*/*z* [M + H]^+^ calcd for C_20_H_27_N_2_O_5_S: 407.1641; found: 407.1644.

#### 1-*tert*-Butyl 5-ethyl 4-(4-methylphenyl)-2-methylthio-1,4-dihydropyrimidine-1,5-dicarboxylate (6c)

Pale yellow oil. ^1^H NMR (CDCl_3_, 600 MHz): *δ* = 1.23 (t, *J* = 7.2 Hz, 3H), 1.59 (s, 9H), 2.29 (s, 3H), 2.32 (s, 3H), 4.14 (dq, *J* = 10.8, 7.2 Hz, 1H), 4.17 (dq, *J* = 10.8, 7.2 Hz, 1H), 5.70 (s, 1H), 7.11 (d, *J* = 8.4 Hz, 2H), 7.19 (d, *J* = 8.4 Hz, 2H), 7.93 (s, 1H). ^13^C NMR (CDCl_3_, 150 MHz): *δ* = 14.1, 15.9, 21.1, 28.0, 58.6, 60.5, 85.7, 111.9, 127.0, 129.1, 132.4, 137.0, 139.4, 148.5, 149.2, 165.3. IR (neat): 2981, 1739, 1712, 1669, 1600, 1371, 1335, 1251, 1154, 1083, 1043 cm^−1^. HRMS-FAB: *m*/*z* [M + H]^+^ calcd for C_20_H_27_N_2_O_4_S: 391.1692; found: 391.1697.

#### 1-*tert*-Butyl 5-ethyl 4-(4-bromophenyl)-2-methylthio-1,4-dihydropyrimidine-1,5-dicarboxylate (6d)

Pale yellow oil. ^1^H NMR (CDCl_3_, 600 MHz): *δ* = 1.23 (t, *J* = 7.2 Hz, 3H), 1.60 (s, 9H), 2.28 (s, 3H), 3.78 (s, 3H), 4.14 (dq, *J* = 10.8, 7.2 Hz, 1H), 4.18 (dq, *J* = 10.8, 7.2 Hz, 1H), 5.68 (s, 1H), 7.17 (d, *J* = 8.4 Hz, 2H), 7.43 (d, *J* = 8.4 Hz, 2H), 7.94 (s, 1H). ^13^C NMR (DMSO-*d*_6_, 150 MHz): *δ* = 14.1, 15.7, 27.6, 58.0, 60.6, 86.1, 110.1, 120.8, 129.4, 131.7, 132.7, 141.8, 148.61, 148.65, 164.4. IR (neat): 2981, 1743, 1711, 1669, 1597, 1486, 1371, 1335, 1251, 1154, 1083, 1043, 1011, 847 cm^−1^. HRMS-FAB: *m*/*z* [M + H]^+^ calcd for C_19_H_24_^79^BrN_2_O_4_S: 455.0640; found: 455.0644.

#### 1-*tert*-Butyl 5-ethyl 4-(4-chlorophenyl)-2-methylthio-1,4-dihydropyrimidine-1,5-dicarboxylate (6e)

Pale yellow oil. ^1^H NMR (CDCl_3_, 600 MHz): *δ* = 1.23 (t, *J* = 7.2 Hz, 3H), 1.60 (s, 9H), 2.28 (s, 3H), 4.14 (dq, *J* = 10.8, 7.2 Hz, 1H), 4.18 (dq, *J* = 10.8, 7.2 Hz, 1H), 5.70 (s, 1H), 7.23 (d, *J* = 8.4 Hz, 2H), 7.28 (d, *J* = 8.4 Hz, 2H), 7.94 (s, 1H). ^13^C NMR (CDCl_3_, 150 MHz): *δ* = 14.1, 15.9, 28.0, 58.3, 60.7, 86.1, 111.2, 128.56, 128.57, 132.7, 133.2, 140.9, 149.0, 149.1, 165.1. IR (neat): 2981, 1744, 1711, 1669, 1598, 1334, 1251, 1233, 1154, 1084, 1043 cm^−1^. HRMS-FAB: *m*/*z* [M + H]^+^ calcd for C_19_H_24_^35^ClN_2_O_4_S: 411.1145; found: 411.1149.

#### 1-*tert*-Butyl 5-ethyl 4-[4-(trifluoromethyl)phenyl]-2-methylthio-1,4-dihydropyrimidine-1,5-dicarboxylate (6f)

Pale yellow oil. ^1^H NMR (CDCl_3_, 600 MHz): *δ* = 1.24 (t, *J* = 7.2 Hz, 3H), 1.60 (s, 9H), 2.29 (s, 3H), 4.15 (dq, *J* = 10.8, 7.2 Hz, 1H), 4.19 (dq, *J* = 10.8, 7.2 Hz, 1H), 5.79 (s, 1H), 7.42 (d, *J* = 8.4 Hz, 2H), 7.57 (d, *J* = 8.4 Hz, 2H), 7.97 (s, 1H). ^13^C NMR (CDCl_3_, 150 MHz): *δ* = 14.1, 15.9, 27.9, 58.5, 60.7, 86.2, 110.8, 124.1 (q, *J* = 270.0 Hz), 125.4 (q, *J* = 3.8 Hz), 127.5, 129.6 (q, *J* = 33.0 Hz), 133.0, 146.2, 149.0, 149.5, 165.0. IR (neat): 2981, 1743, 1711, 1669, 1598, 1371, 1334, 1251, 1233, 1154, 1084, 1043 cm^−1^. HRMS-FAB: *m*/*z* [M + H]^+^ calcd for C_20_H_24_F_3_N_2_O_4_S: 445.1409; found: 445.1406.

#### 1-*tert*-Butyl 5-ethyl 4-(2-chlorophenyl)-2-methylthio-1,4-dihydropyrimidine-1,5-dicarboxylate (6g)

Pale yellow oil. ^1^H NMR (CDCl_3_, 600 MHz): *δ* = 1.15 (t, *J* = 7.2 Hz, 3H), 1.61 (s, 9H), 2.22 (s, 3H), 4.08 (dq, *J* = 10.8, 7.2 Hz, 1H), 4.12 (dq, *J* = 10.8, 7.2 Hz, 1H), 6.12 (s, 1H), 7.15–7.23 (m, 3H), 7.38 (dd, *J* = 7.2, 1.2 Hz, 1H), 8.10 (s, 1H). ^13^C NMR (CDCl_3_, 150 MHz): *δ* = 14.0, 15.9, 28.0, 56.2, 60.5, 85.9, 109.7, 127.0, 128.6, 128.9, 129.8, 133.6, 133.8, 139.6, 147.7, 149.1, 165.1. IR (neat): 2982, 1739, 1714, 1671, 1600, 1337, 1253, 1221, 1155, 1087, 1036 cm^−1^. HRMS-FAB: *m*/*z* [M + H]^+^ calcd for C_19_H_24_^35^ClN_2_O_4_S: 411.1145; found: 411.1154.

#### 1-*tert*-Butyl 5-ethyl 4-cyclohexyl-2-methylthio-1,4-dihydropyrimidine-1,5-dicarboxylate (6i)

Colorless crystals, mp 111–112 °C (*n*-hexane–EtOAc). ^1^H NMR (CDCl_3_, 600 MHz): *δ* = 0.85–0.94 (m, 1H), 1.05–1.37 (m, 4H), 1.29 (t, *J* = 7.2 Hz, 3H), 1.48–1.77 (m, 6H), 1.58 (s, 9H), 2.31 (s, 3H), 4.20 (dq, *J* = 10.8, 7.2 Hz, 1H), 4.23 (dq, *J* = 10.8, 7.2 Hz, 1H), 4.54 (d, 1H, *J* = 4.8 Hz), 7.83 (s, 1H). ^13^C NMR (CDCl_3_, 150 MHz): *δ* = 14.2, 15.7, 26.1, 26.35, 26.41, 27.4, 28.0, 29.2, 44.3, 60.3, 60.4, 85.3, 111.3, 133.3, 147.3, 149.3, 165.8. IR (KBr): 2923, 2848, 1743, 1709, 1663, 1604, 1370, 1332, 1260, 1235, 1146, 1071, 1020 cm^−1^. HRMS-FAB: *m*/*z* [M + H]^+^ calcd for C_19_H_31_N_2_O_4_S: 383.2005; found: 383.2010.

### General procedure for synthesis of 2-aryl-DPs 7 and 9

#### 1-*tert*-Butyl 5-ethyl 2,4-diphenyl-1,4-dihydropyrimidine-1,5-dicarboxylate (7a)

Under an atmosphere of Ar, a mixture of 6a (94.0 mg, 0.250 mmol, 1.0 equiv.), phenyltributylstannane 2a (184 mg, 0.501 mmol, 2.0 equiv.), Pd_2_dba_3_ (5.8 mg, 0.00633 mmol, 0.025 equiv.), (2-furyl)_3_P (11.6 mg, 0.0500 mmol, 0.20 equiv.), and CuTC (96 mg, 0.503 mmol, 2.0 equiv.) in CH_2_Cl_2_ (3.0 mL) was heated at reflux for 30 h. The mixture was filtered through a Celite pad and washed with EtOAc (20 mL). The filtrate was washed with aqueous 1 M NaOH solution (10 mL), and the organic layer was separated. The aqueous layer was extracted with EtOAc (10 mL). The combined organic layers were washed with water (5 mL) and brine (5 mL), dried over anhydrous Na_2_SO_4_, filtered, and concentrated under reduced pressure. The residue was purified by flash column chromatography (silica gel–K_2_CO_3_, 10 : 1;^24^ eluent: *n-*hexane–EtOAc, 11 : 1 to 6 : 1) to give 7a (93.0 mg, 0.229 mmol, 91%) as colorless crystals. Mp 139–141 °C (*n*-hexane–EtOAc). ^1^H NMR (CDCl_3_, 600 MHz): *δ* = 1.18 (s, 9H), 1.28 (t, *J* = 7.2 Hz, 3H), 4.20 (dq, *J* = 10.8, 7.2 Hz, 1H), 4.24 (dq, *J* = 10.8, 7.2 Hz, 1H), 5.94 (s, 1H), 7.28 (t, *J* = 7.8 Hz, 1H), 7.32–7.44 (m, 7H), 7.47 (d, *J* = 7.8 Hz, 2H), 8.13 (d, *J* = 1.2 Hz, 1H). ^13^C NMR (CDCl_3_, 150 MHz): *δ* = 14.2, 27.3, 58.7, 60.7, 84.6, 114.2, 127.0, 127.2, 127.5, 128.1, 128.7, 129.7, 133.6, 136.7, 141.0, 149.5, 151.3, 165.0. IR (KBr): 2981, 1726, 1709, 1673, 1353, 1267, 1243, 1154, 1070, 754, 703 cm^−1^. HRMS-FAB: *m*/*z* [M + H]^+^ calcd for C_24_H_27_N_2_O_4_: 407.1971; found: 407.1975.

#### 1-*tert*-Butyl 5-ethyl 2-(4-methoxyphenyl)-4-phenyl-1,4-dihydropyrimidine-1,5-dicarboxylate (7b)

Eluent in chromatography: *n-*hexane–EtOAc, 6 : 1 to 4 : 1. Yield: 98%; pale yellow oil. ^1^H NMR (CDCl_3_, 600 MHz): *δ* = 1.23 (s, 9H), 1.28 (t, *J* = 7.2 Hz, 3H), 3.83 (s, 3H), 4.20 (dq, *J* = 10.8, 7.2 Hz, 1H), 4.24 (dq, *J* = 10.8, 7.2 Hz, 1H), 5.92 (s, 1H), 6.89 (d, *J* = 9.0 Hz, 2H), 7.27 (t, *J* = 7.8 Hz, 1H), 7.33 (t, *J* = 7.8 Hz, 2H), 7.37 (d, *J* = 7.8 Hz, 2H), 7.43 (d, *J* = 9.0 Hz, 2H), 8.09 (d, *J* = 1.2 Hz, 1H). ^13^C NMR (CDCl_3_, 150 MHz): *δ* = 14.2, 27.4, 55.4, 58.5, 60.7, 84.3, 113.4, 114.6, 126.9, 127.4, 128.6, 128.8, 128.9, 133.7, 141.0, 149.6, 151.1, 161.0, 165.0. IR (neat): 2980, 1733, 1711, 1669, 1609, 1514, 1354, 1250, 1152, 1025 cm^−1^. HRMS-FAB: *m*/*z* [M + H]^+^ calcd for C_25_H_29_N_2_O_5_: 437.2076; found: 437.2094.

#### 1-*tert*-Butyl 5-ethyl 2-(4-methylphenyl)-4-phenyl-1,4-dihydropyrimidine-1,5-dicarboxylate (7c)

Eluent in chromatography: *n-*hexane–EtOAc, 11 : 1 to 6 : 1. Yield: 95%; pale yellow oil. ^1^H NMR (CDCl_3_, 600 MHz): *δ* = 1.20 (s, 9H), 1.28 (t, *J* = 7.2 Hz, 3H), 2.38 (s, 3H), 4.19 (dq, *J* = 10.8, 7.2 Hz, 1H), 4.24 (dq, *J* = 10.8, 7.2 Hz, 1H), 5.93 (s, 1H), 7.17 (d, *J* = 8.4 Hz, 2H), 7.27 (t, *J* = 7.2 Hz, 1H), 7.33 (t, *J* = 7.2 Hz, 2H), 7.35–7.40 (m, 4H), 8.10 (d, *J* = 1.2 Hz, 1H). ^13^C NMR (CDCl_3_, 150 MHz): *δ* = 14.2, 21.3, 27.4, 58.6, 60.6, 84.4, 114.3, 126.9, 127.2, 127.4, 128.6, 128.7, 133.6, 133.7, 139.8, 141.0, 149.5, 151.4, 165.0. IR (neat): 2980, 1734, 1712, 1670, 1615, 1354, 1315, 1246, 1152, 1028 cm^−1^. HRMS-FAB: *m*/*z* [M + H]^+^ calcd for C_25_H_29_N_2_O_4_: 421.2127; found: 421.2135.

#### 1-*tert*-Butyl 5-ethyl 4-phenyl-2-[(1,1′-biphenyl)-4-yl]-1,4-dihydropyrimidine-1,5-dicarboxylate (7d)

Eluent in chromatography: *n-*hexane–EtOAc, 12 : 1 to 5 : 1. Yield: 88%; pale yellow amorphous. ^1^H NMR (CDCl_3_, 600 MHz): *δ* = 1.21 (s, 9H), 1.29 (t, *J* = 7.2 Hz, 3H), 4.20 (dq, *J* = 10.8, 7.2 Hz, 1H), 4.25 (dq, *J* = 10.8, 7.2 Hz, 1H), 5.95 (s, 1H), 7.29 (t, *J* = 7.2 Hz, 1H), 7.35 (t, *J* = 7.2 Hz, 2H), 7.37 (t, *J* = 7.2 Hz, 1H), 7.40 (d, *J* = 7.2 Hz, 2H), 7.45 (t, *J* = 7.2 Hz, 2H), 7.54 (d, *J* = 8.4 Hz, 2H), 7.60 (d, *J* = 7.2 Hz, 2H), 7.61 (d, *J* = 8.4 Hz, 2H), 8.14 (d, 1H, *J* = 1.2 Hz). ^13^C NMR (CDCl_3_, 150 MHz): *δ* = 14.2, 27.4, 58.7, 60.7, 84.6, 114.3, 126.7, 127.0, 127.1, 127.5, 127.70, 127.73, 128.6, 128.8, 133.6, 135.5, 140.3, 141.0, 142.6, 149.4, 151.0, 165.0. IR (KBr): 2980, 1734, 1711, 1669, 1370, 1355, 1246, 1152, 754 cm^−1^. HRMS-FAB: *m*/*z* [M + H]^+^ calcd for C_30_H_31_N_2_O_4_: 483.2284; found: 483.2292.

#### 1-*tert*-Butyl 5-ethyl 2-[4-(trifluoromethyl)phenyl]-4-phenyl-1,4-dihydropyrimidine-1,5-dicarboxylate (7e)

Eluent in chromatography: *n-*hexane–EtOAc, 12 : 1 to 6 : 1. Yield: 79%; pale yellow oil. ^1^H NMR (CDCl_3_, 600 MHz): *δ* = 1.21 (s, 9H), 1.28 (t, *J* = 7.2 Hz, 3H), 4.20 (dq, *J* = 10.8, 7.2 Hz, 1H), 4.24 (dq, *J* = 10.8, 7.2 Hz, 1H), 5.93 (s, 1H), 7.30 (tt, *J* = 6.6, 1.8 Hz, 1H), 7.33–7.39 (m, 4H), 7.58 (d, *J* = 8.4 Hz, 2H), 7.64 (d, *J* = 8.4 Hz, 2H), 8.12 (d, *J* = 0.6 Hz, 1H). ^13^C NMR (CDCl_3_, 150 MHz): *δ* = 14.2, 27.4, 58.9, 60.8, 85.1, 114.2, 123.8 (q, *J* = 271.5 Hz), 125.1 (q, *J* = 3.5 Hz), 127.0, 127.6, 127.8, 128.8, 131.6 (q, *J* = 33.0 Hz), 133.3, 140.2, 140.6, 149.0, 149.9, 164.8. IR (neat): 2981, 1739, 1713, 1673, 1326, 1247, 1154, 1068, 1025, 851 cm^−1^. HRMS-FAB: *m*/*z* [M + H]^+^ calcd for C_25_H_26_F_3_N_2_O_4_: 475.1845; found: 475.1855.

#### 1-*tert*-Butyl 5-ethyl 2-[4-(methoxycarbonyl)phenyl]-4-phenyl-1,4-dihydropyrimidine-1,5-dicarboxylate (7f)

Eluent in chromatography: *n-*hexane–EtOAc, 8 : 1 to 4 : 1. Yield: 86%; pale yellow oil. ^1^H NMR (CDCl_3_, 600 MHz): *δ* = 1.19 (s, 9H), 1.28 (t, *J* = 7.2 Hz, 3H), 3.94 (s, 3H), 4.20 (dq, *J* = 10.8, 7.2 Hz, 1H), 4.24 (dq, *J* = 10.8, 7.2 Hz, 1H), 5.94 (s, 1H), 7.30 (tt, *J* = 7.2, 1.8 Hz, 1H), 7.35 (t, *J* = 7.2 Hz, 2H), 7.38 (dd, *J* = 7.2, 1.8 Hz, 2H), 7.54 (d, *J* = 8.4 Hz, 2H), 8.05 (d, *J* = 8.4 Hz, 2H), 8.12 (d, *J* = 1.2 Hz, 1H). ^13^C NMR (CDCl_3_, 150 MHz): *δ* = 14.2, 27.4, 52.2, 58.9, 60.8, 85.0, 114.2, 127.0, 127.3, 127.7, 128.7, 129.4, 131.0, 133.3, 140.7, 141.0, 149.1, 150.3, 164.8, 166.4. IR (neat): 2980, 1723, 1671, 1355, 1280, 1247, 1152 cm^−1^. HRMS-FAB: *m*/*z* [M + H]^+^ calcd for C_26_H_29_N_2_O_6_: 465.2026; found: 465.2025.

#### 1-*tert*-Butyl 5-ethyl 2-(4-nitrophenyl)-4-phenyl-1,4-dihydropyrimidine-1,5-dicarboxylate (7g)

Eluent in chromatography: *n-*hexane–EtOAc, 10 : 1 to 5 : 1. Yield: 86%; pale yellow oil. ^1^H NMR (CDCl_3_, 600 MHz): *δ* = 1.26 (s, 9H), 1.28 (t, *J* = 7.2 Hz, 3H), 4.20 (dq, *J* = 10.8, 7.2 Hz, 1H), 4.24 (dq, *J* = 10.8, 7.2 Hz, 1H), 5.94 (s, 1H), 7.29–7.39 (m, 5H), 7.63 (d, *J* = 8.4 Hz, 2H), 8.10 (s, 1H), 8.24 (d, *J* = 8.4 Hz, 2H). ^13^C NMR (CDCl_3_, 150 MHz): *δ* = 14.1, 27.5, 59.1, 60.9, 85.4, 114.3, 123.3, 127.0, 127.9, 128.2, 128.8, 133.0, 140.3, 142.7, 148.3, 148.8, 149.1, 164.6. IR (neat): 2980, 1739, 1712, 1672, 1600, 1524, 1348, 1246, 1152 cm^−1^. HRMS-FAB: *m*/*z* [M + H]^+^ calcd for C_24_H_26_N_3_O_6_: 452.1822; found: 452.1831.

#### 1-*tert*-Butyl 5-ethyl 2-(3-nitrophenyl)-4-phenyl-1,4-dihydropyrimidine-1,5-dicarboxylate (7h)

Eluent in chromatography: *n-*hexane–EtOAc, 10 : 1 to 4 : 1. Yield: 86%; pale yellow oil. ^1^H NMR (CDCl_3_, 600 MHz): *δ* = 1.26 (s, 9H), 1.28 (t, *J* = 7.2 Hz, 3H), 4.20 (dq, *J* = 10.8, 7.2 Hz, 1H), 4.25 (dq, *J* = 10.8, 7.2 Hz, 1H), 5.94 (d, *J* = 1.2 Hz, 1H), 7.29–7.40 (m, 5H), 7.57 (t, *J* = 7.8 Hz, 1H), 7.83 (ddd, *J* = 7.8, 1.8, 1.2 Hz, 1H), 8.12 (d, *J* = 1.2 Hz, 1H), 8.28 (ddd, *J* = 7.8, 2.4, 1.2 Hz, 1H), 8.31 (dd, *J* = 2.4, 1.8 Hz, 1H). ^13^C NMR (CDCl_3_, 150 MHz): *δ* = 14.1, 27.5, 59.0, 60.9, 85.4, 114.5, 122.3, 124.3, 127.0, 127.9, 128.8, 129.2, 133.15, 133.22, 138.3, 140.3, 147.8, 148.8, 148.9, 164.6. IR (neat): 2979, 1738, 1712, 1674, 1616, 1533, 1348, 1318, 1245, 1152, 1024, 752 cm^−1^. HRMS-FAB: *m*/*z* [M + H]^+^ calcd for C_24_H_26_N_3_O_6_: 452.1822; found: 452.1825.

#### 1-*tert*-Butyl 5-ethyl 2-[3,5-bis(trifluoromethyl)phenyl]-4-phenyl-1,4-dihydropyrimidine-1,5-dicarboxylate (7i)

Eluent in chromatography: *n-*hexane–EtOAc, 15 : 1. Yield: 89%; pale yellow oil. ^1^H NMR (CDCl_3_, 600 MHz): *δ* = 1.24 (s, 9H), 1.28 (t, *J* = 7.2 Hz, 3H), 4.19 (dq, *J* = 10.8, 7.2 Hz, 1H), 4.25 (dq, *J* = 10.8, 7.2 Hz, 1H), 5.94 (s, 1H), 7.29–7.40 (m, 5H), 7.90 (s, 2H), 7.93 (s, 1H), 8.12 (d, *J* = 0.6 Hz, 1H). ^13^C NMR (CDCl_3_, 150 MHz): *δ* = 14.1, 27.4, 59.2, 60.9, 85.6, 114.6, 122.98 (q, *J* = 271.5 Hz), 123.04 (q, *J* = 2.7 Hz), 127.1, 127.5, 128.0, 128.9, 131.7 (q, *J* = 33.0 Hz), 133.0, 138.8, 140.2, 148.4, 148.7, 164.5. IR (neat): 2982, 1743, 1714, 1675, 1341, 1280, 1244, 1150 cm^−1^. HRMS-FAB: *m*/*z* [M + H]^+^ calcd for C_26_H_25_F_6_N_2_O_4_: 543.1719; found: 543.1704.

#### 1-*tert*-Butyl 5-ethyl 4-phenyl-2-(thiophen-2-yl)-1,4-dihydropyrimidine-1,5-dicarboxylate (7j)

Eluent in chromatography: *n-*hexane–EtOAc, 11 : 1 to 6 : 1. Yield: 91%; pale yellow oil. ^1^H NMR (CDCl_3_, 600 MHz): *δ* = 1.28 (t, *J* = 7.2 Hz, 3H), 1.32 (s, 9H), 4.20 (dq, *J* = 10.8, 7.2 Hz, 1H), 4.24 (dq, *J* = 10.8, 7.2 Hz, 1H), 5.94 (s, 1H), 7.03 (dd, *J* = 4.8, 3.6 Hz, 1H), 7.23 (dd, *J* = 3.6, 1.2 Hz, 1H), 7.26 (t, *J* = 7.2 Hz, 1H), 7.32 (t, *J* = 7.2 Hz, 2H), 7.35 (d, *J* = 7.2 Hz, 2H), 7.38 (dd, *J* = 4.8, 1.2 Hz, 1H), 8.01 (d, *J* = 1.2 Hz, 1H). ^13^C NMR (CDCl_3_, 150 MHz): *δ* = 14.2, 27.5, 58.6, 60.7, 84.6, 115.6, 126.7, 126.8, 127.5, 127.8, 128.0, 128.6, 133.7, 138.4, 140.4, 146.6, 149.4, 164.8. IR (neat): 2978, 1735, 1711, 1664, 1340, 1245, 1151 cm^−1^. HRMS-FAB: *m*/*z* [M + H]^+^ calcd for C_22_H_25_N_2_O_4_S: 413.1535; found: 413.1534.

#### 1-*tert*-Butyl 5-ethyl 4-phenyl-2-(thiophen-3-yl)-1,4-dihydropyrimidine-1,5-dicarboxylate (7k)

Eluent in chromatography: *n-*hexane–EtOAc, 8 : 1 to 4 : 1. Yield: 79%; pale yellow oil. ^1^H NMR (CDCl_3_, 600 MHz): *δ* = 1.276 (t, *J* = 7.2 Hz, 3H), 1.281 (s, 9H), 4.19 (dq, *J* = 10.8, 7.2 Hz, 1H), 4.23 (dq, *J* = 10.8, 7.2 Hz, 1H), 5.90 (s, 1H), 7.16 (dd, *J* = 4.8, 1.2 Hz, 1H), 7.26–7.30 (m, 2H), 7.33 (t, *J* = 7.8 Hz, 2H), 7.37 (d, *J* = 7.8 Hz, 2H), 7.54 (dd, *J* = 3.0, 1.2 Hz, 1H), 8.07 (d, *J* = 1.2 Hz, 1H). ^13^C NMR (CDCl_3_, 150 MHz): *δ* = 14.2, 27.4, 58.5, 60.7, 84.5, 114.4, 125.2, 125.8, 126.8, 126.9, 127.5, 128.6, 133.5, 137.5, 140.8, 147.0, 149.4, 164.9. IR (neat): 2980, 1733, 1711, 1669, 1371, 1342, 1245, 1151, 1025 cm^−1^. HRMS-FAB: *m*/*z* [M + H]^+^ calcd for C_22_H_25_N_2_O_4_S: 413.1535; found: 413.1527.

#### 1-*tert*-Butyl 5-ethyl 4-phenyl-2-(pyridin-2-yl)-1,4-dihydropyrimidine-1,5-dicarboxylate (7l)

Tributyl(pyridin-2-yl)stannane (4.0 equiv.), Pd_2_dba_3_ (5.0 mol%), (2-furyl)_3_P (40 mol%), and CuTC (4.0 equiv.) were used. Eluent in chromatography: *n-*hexane–EtOAc–Et_3_N, 80 : 20 : 1 to 20 : 40 : 1. Yield: 31%; pale yellow oil. ^1^H NMR (CDCl_3_, 600 MHz): *δ* = 1.22 (s, 9H), 1.26 (t, *J* = 7.2 Hz, 3H), 4.17 (dq, *J* = 10.8, 7.2 Hz, 1H), 4.23 (dq, *J* = 10.8, 7.2 Hz, 1H), 5.94 (s, 1H), 7.29 (t, *J* = 7.2 Hz, 1H), 7.33–7.38 (m, 3H), 7.41 (d, *J* = 7.2 Hz, 2H), 7.68 (d, *J* = 7.8 Hz, 1H), 7.75 (ddd, *J* = 7.8, 7.8, 1.8 Hz, 1H), 8.17 (d, *J* = 1.2 Hz, 1H), 8.55–8.57 (m, 1H). ^13^C NMR (CDCl_3_, 150 MHz): *δ* = 14.2, 27.4, 58.9, 60.6, 84.2, 112.5, 123.1, 124.3, 127.1, 127.6, 128.7, 133.6, 136.8, 141.0, 148.0, 149.4, 150.4, 154.2, 165.0. IR (neat): 2980, 2932, 1741, 1711, 1671, 1362, 1321, 1244, 1155, 1075, 1025, 750 cm^−1^. HRMS-FAB: *m*/*z* [M + H]^+^ calcd for C_23_H_26_N_3_O_4_: 408.1923; found: 408.1927.

#### 1-*tert*-Butyl 5-ethyl 4-phenyl-2-(pyridin-3-yl)-1,4-dihydropyrimidine-1,5-dicarboxylate (7m)

Eluent in chromatography: *n-*hexane–EtOAc, 5 : 1 to 1 : 2. Yield: 33%; colorless crystals, mp 107–108 °C (*n*-hexane–EtOAc). ^1^H NMR (CDCl_3_, 600 MHz): *δ* = 1.24 (s, 9H), 1.28 (t, *J* = 7.2 Hz, 3H), 4.20 (dq, *J* = 10.8, 7.2 Hz, 1H), 4.24 (dq, *J* = 10.8, 7.2 Hz, 1H), 5.95 (s, 1H), 7.28–7.39 (m, 6H), 7.80 (dt, *J* = 7.8, 1.8 Hz, 1H), 8.13 (d, *J* = 1.2 Hz, 1H), 8.65 (dd, *J* = 4.8, 1.8 Hz, 1H), 8.69 (d, *J* = 1.8 Hz, 1H). ^13^C NMR (CDCl_3_, 150 MHz): *δ* = 14.2, 27.5, 58.9, 60.8, 85.2, 114.3, 123.0, 127.0, 127.8, 128.8, 132.7, 133.2, 134.8, 140.6, 148.1, 148.7, 149.0, 150.4, 164.7. IR (KBr): 2980, 1726, 1711, 1673, 1356, 1312, 1244, 1154, 1071 cm^−1^. HRMS-FAB: *m*/*z* [M + H]^+^ calcd for C_23_H_26_N_3_O_4_: 408.1923; found: 408.1918.

#### 1-*tert*-Butyl 5-ethyl 4-(4-methoxyphenyl)-2-phenyl-1,4-dihydropyrimidine-1,5-dicarboxylate (7n)

Eluent in chromatography: *n-*hexane–EtOAc, 6 : 1 to 3 : 1. Yield: 92%; pale yellow oil. ^1^H NMR (CDCl_3_, 600 MHz): *δ* = 1.18 (s, 9H), 1.28 (t, *J* = 7.2 Hz, 3H), 3.80 (s, 3H), 4.19 (dq, *J* = 10.8, 7.2 Hz, 1H), 4.23 (dq, *J* = 10.8, 7.2 Hz, 1H), 5.86 (s, 1H), 6.87 (d, *J* = 9.0 Hz, 2H), 7.30 (d, *J* = 9.0 Hz, 2H), 7.36 (t, *J* = 7.2 Hz, 2H), 7.41 (tt, *J* = 7.2, 1.8 Hz, 1H), 7.45 (dd, *J* = 7.2, 1.8 Hz, 2H), 8.12 (d, *J* = 0.6 Hz, 1H). ^13^C NMR (CDCl_3_, 150 MHz): *δ* = 14.2, 27.3, 55.2, 58.1, 60.6, 84.5, 114.0, 114.3, 127.2, 128.05, 128.10, 129.6, 133.28, 133.33, 136.7, 149.5, 150.9, 159.0, 165.0. IR (neat): 2980, 1734, 1712, 1670, 1610, 1511, 1354, 1317, 1247, 1153, 1035 cm^−1^. HRMS-FAB: *m*/*z* [M + H]^+^ calcd for C_25_H_29_N_2_O_5_: 437.2076; found: 437.2081.

#### 1-*tert*-Butyl 5-ethyl 4-(4-methylphenyl)-2-phenyl-1,4-dihydropyrimidine-1,5-dicarboxylate (7o)

Eluent in chromatography: *n-*hexane–EtOAc, 10 : 1 to 5 : 1. Yield: 89%; pale yellow oil. ^1^H NMR (CDCl_3_, 600 MHz): *δ* = 1.17 (s, 9H), 1.28 (t, *J* = 7.2 Hz, 3H), 2.34 (s, 3H), 4.19 (dq, *J* = 10.8, 7.2 Hz, 1H), 4.23 (dq, *J* = 10.8, 7.2 Hz, 1H), 5.89 (s, 1H), 7.15 (d, *J* = 8.4 Hz, 2H), 7.27 (d, *J* = 8.4 Hz, 2H), 7.36 (t, *J* = 7.2 Hz, 2H), 7.41 (tt, *J* = 7.2, 1.8 Hz, 1H), 7.46 (dd, *J* = 7.2, 1.8 Hz, 2H), 8.12 (d, *J* = 1.2 Hz, 1H). ^13^C NMR (CDCl_3_, 150 MHz): *δ* = 14.2, 21.1, 27.3, 58.5, 60.6, 84.4, 114.3, 126.9, 127.2, 128.1, 129.3, 129.6, 133.4, 136.7, 137.2, 138.1, 149.5, 151.1, 165.0. IR (KBr): 2979, 1734, 1712, 1669, 1354, 1245, 1151 cm^−1^. HRMS-FAB: *m*/*z* [M + H]^+^ calcd for C_25_H_29_N_2_O_4_: 421.2127; found: 421.2131.

#### 1-*tert*-Butyl 5-ethyl 4-(4-bromophenyl)-2-phenyl-1,4-dihydropyrimidine-1,5-dicarboxylate (7p)

Eluent in chromatography: *n-*hexane–EtOAc, 10 : 1 to 5 : 1. Yield: 86%; pale yellow oil. ^1^H NMR (CDCl_3_, 600 MHz): *δ* = 1.17 (s, 9H), 1.29 (t, *J* = 7.2 Hz, 3H), 4.20 (dq, *J* = 10.8, 7.2 Hz, 1H), 4.24 (dq, *J* = 10.8, 7.2 Hz, 1H), 5.88 (s, 1H), 7.26 (d, *J* = 8.4 Hz, 2H), 7.38 (t, *J* = 7.2 Hz, 2H), 7.41–7.46 (m, 3H), 7.47 (d, *J* = 8.4 Hz, 2H), 8.14 (d, *J* = 0.6 Hz, 1H). ^13^C NMR (CDCl_3_, 150 MHz): *δ* = 14.2, 27.3, 58.1, 60.8, 84.8, 113.5, 121.5, 127.2, 128.1, 128.7, 129.8, 131.7, 133.8, 136.5, 140.1, 149.3, 151.5, 164.8. IR (neat): 2980, 1737, 1711, 1671, 1371, 1353, 1245, 1152, 1011 cm^−1^. HRMS-FAB: *m*/*z* [M + H]^+^ calcd for C_24_H_26_^79^BrN_2_O_4_: 485.1076; found: 485.1068.

#### 1-*tert*-Butyl 5-ethyl 4-(4-chlorophenyl)-2-phenyl-1,4-dihydropyrimidine-1,5-dicarboxylate (7q)

Eluent in chromatography: *n-*hexane–EtOAc, 8 : 1 to 5 : 1. Yield: 84%; pale yellow oil. ^1^H NMR (CDCl_3_, 600 MHz): *δ* = 1.17 (s, 9H), 1.29 (t, *J* = 7.2 Hz, 3H), 4.20 (dq, *J* = 10.8, 7.2 Hz, 1H), 4.24 (dq, *J* = 10.8, 7.2 Hz, 1H), 5.89 (d, *J* = 1.2 Hz, 1H), 7.32 (s, 4H), 7.38 (t, *J* = 7.2 Hz, 2H), 7.41–7.46 (m, 3H), 8.14 (d, *J* = 1.2 Hz, 1H). ^13^C NMR (CDCl_3_, 150 MHz): *δ* = 14.2, 27.3, 58.0, 60.8, 84.8, 113.6, 127.2, 128.1, 128.3, 128.8, 129.8, 133.3, 133.8, 136.5, 139.5, 149.3, 151.5, 164.8. IR (neat): 2980, 1737, 1711, 1671, 1371, 1353, 1246, 1153, 1015 cm^−1^. HRMS-FAB: *m*/*z* [M + H]^+^ calcd for C_24_H_26_^35^ClN_2_O_4_: 441.1581; found: 441.1575.

#### 1-*tert*-Butyl 5-ethyl 4-[4-(trifluoromethyl)phenyl]-2-phenyl-1,4-dihydropyrimidine-1,5-dicarboxylate (7r)

Eluent in chromatography: *n-*hexane–EtOAc, 7 : 1 to 5 : 1. Yield: 84%; pale yellow oil. ^1^H NMR (CDCl_3_, 600 MHz): *δ* = 1.18 (s, 9H), 1.29 (t, *J* = 7.2 Hz, 3H), 4.21 (dq, *J* = 10.8, 7.2 Hz, 1H), 4.25 (dq, *J* = 10.8, 7.2 Hz, 1H), 5.98 (s, 1H), 7.39 (t, *J* = 7.8 Hz, 2H), 7.44 (tt, *J* = 7.8, 1.2 Hz, 1H), 7.46 (dd, *J* = 7.8, 1.2 Hz, 2H), 7.51 (d, *J* = 7.8 Hz, 2H), 7.61 (d, *J* = 7.8 Hz, 2H), 8.16 (d, *J* = 1.2 Hz, 1H). ^13^C NMR (CDCl_3_, 150 MHz): *δ* = 14.2, 27.3, 58.3, 60.9, 84.9, 113.3, 124.1 (*J* = 271.5 Hz), 125.6 (*J* = 3.8 Hz), 127.2, 127.3, 128.2, 129.8 (q, *J* = 31.5 Hz), 129.9, 134.0, 136.4, 144.9, 149.3, 151.8, 164.8. IR (neat): 2982, 1738, 1711, 1672, 1618, 1354, 1326, 1245, 1152, 1125, 1067 cm^−1^. HRMS-FAB: *m*/*z* [M + H]^+^ calcd for C_25_H_26_F_3_N_2_O_4_: 475.1845; found: 475.1850.

#### 1-*tert*-Butyl 5-ethyl 4-(2-chlorophenyl)-2-phenyl-1,4-dihydropyrimidine-1,5-dicarboxylate (7s)

Eluent in chromatography: *n-*hexane–EtOAc, 10 : 1 to 5 : 1. Yield: 87%; colorless crystals, mp 135–136 °C (*n*-hexane–EtOAc). ^1^H NMR (CDCl_3_, 600 MHz): *δ* = 1.17 (s, 9H), 1.22 (t, *J* = 7.2 Hz, 3H), 4.14 (dq, *J* = 10.8, 7.2 Hz, 1H), 4.17 (dq, *J* = 10.8, 7.2 Hz, 1H), 6.27 (s, 1H), 7.20–7.25 (m, 3H), 7.33 (t, *J* = 7.2 Hz, 2H), 7.35–7.40 (m, 3H), 7.42–7.46 (m, 1H), 8.29 (s, 1H). ^13^C NMR (CDCl_3_, 150 MHz): *δ* = 14.1, 27.3, 56.5, 60.7, 84.6, 112.3, 127.1, 127.2, 128.0, 128.7, 128.9, 129.5, 130.1, 134.1, 134.9, 136.9, 138.4, 149.5, 150.6, 164.7. IR (KBr): 2978, 1728, 1711, 1665, 1350, 1262, 1249, 1156 cm^−1^. HRMS-FAB: *m*/*z* [M + H]^+^ calcd for C_24_H_26_^35^ClN_2_O_4_: 441.1581; found: 441.1588.

#### 1-*tert*-Butyl 5-ethyl 2-phenyl-4-propyl-1,4-dihydropyrimidine-1,5-dicarboxylate (7t)

Eluent in chromatography: *n-*hexane–EtOAc, 10 : 1 to 5 : 1. Yield: 84%; pale yellow oil. ^1^H NMR (CDCl_3_, 600 MHz): *δ* = 0.98 (t, *J* = 7.2 Hz, 3H), 1.18 (s, 9H), 1.32 (t, *J* = 7.2 Hz, 3H), 1.43–1.70 (m, 4H), 4.23 (dq, *J* = 10.8, 7.2 Hz, 1H), 4.26 (dq, *J* = 10.8, 7.2 Hz, 1H), 4.80 (t, *J* = 6.0 Hz, 1H), 7.36 (t, *J* = 7.2 Hz, 2H), 7.40 (tt, *J* = 7.2, 1.8 Hz, 1H), 7.43 (dd, *J* = 7.2, 1.8 Hz, 2H), 8.02 (d, *J* = 1.2 Hz, 1H). ^13^C NMR (CDCl_3_, 150 MHz): *δ* = 14.1, 14.2, 18.4, 27.3, 38.0, 55.0, 60.5, 84.2, 115.1, 127.1, 128.0, 129.4, 133.7, 137.0, 149.6, 150.6, 165.2. IR (neat): 2960, 2935, 1733, 1712, 1670, 1370, 1351, 1245, 1153 cm^−1^. HRMS-FAB: *m*/*z* [M + H]^+^ calcd for C_21_H_29_N_2_O_4_: 373.2127; found: 373.2135.

#### 1-*tert*-Butyl 5-ethyl 4-cyclohexyl-2-phenyl-1,4-dihydropyrimidine-1,5-dicarboxylate (7u)

Eluent in chromatography: *n-*hexane–EtOAc, 20 : 1 to 6 : 1. Yield: 81%; pale yellow oil. ^1^H NMR (CDCl_3_, 600 MHz): *δ* = 1.02–1.45 (m, 5H), 1.18 (s, 9H), 1.32 (t, *J* = 7.2 Hz, 3H), 1.60–1.89 (m, 6H), 4.22 (dq, *J* = 10.8, 7.2 Hz, 1H), 4.26 (dq, *J* = 10.8, 7.2 Hz, 1H), 4.73 (d, *J* = 5.4 Hz, 1H), 7.36 (t, *J* = 7.2 Hz, 2H), 7.40 (t, *J* = 7.2 Hz, 1H), 7.45 (d, *J* = 7.2 Hz, 2H), 8.03 (d, *J* = 1.2 Hz, 1H). ^13^C NMR (CDCl_3_, 150 MHz): *δ* = 14.2, 26.3, 26.4, 27.4, 27.7, 29.2, 44.1, 60.2, 60.5, 84.0, 113.9, 127.1, 128.0, 129.4, 133.9, 137.0, 149.6, 150.6, 165.5. IR (neat): 2928, 2853, 1731, 1713, 1670, 1371, 1351, 1318, 1244, 1154, 1012 cm^−1^. HRMS-FAB: *m*/*z* [M + H]^+^ calcd for C_24_H_33_N_2_O_4_: 413.2440; found: 413.2446.

#### Ethyl 4,4,6-trimethyl-2-phenyl-1,4-dihydropyrimidine-5-carboxylate (9)

Eluent in chromatography: *n-*hexane–EtOAc–Et_3_N, 150 : 50 : 1 to 100 : 50 : 1. Yield: 71%; colorless crystals, mp 86–88 °C (*n*-hexane-Et_2_O). ^1^H NMR (CD_3_OD, 500 MHz): *δ* = 1.31 (t, *J* = 7.5 Hz, 3H), 1.47 (s, 6H), 2.09 (s, 3H), 4.20 (q, *J* = 7.5 Hz, 2H), 7.45 (t, *J* = 7.0 Hz, 2H), 7.51 (t, *J* = 7.0 Hz, 1H), 7.67 (d, *J* = 7.0 Hz, 2H). ^13^C NMR (CD_3_OD, 125 MHz): *δ* = 14.6, 19.9, 30.2, 54.9, 61.1, 109.7, 128.6, 129.5, 131.9, 135.7, 146.4 (br), 155.8 (br), 169.3. IR (neat): 2969, 1690, 1644, 1478, 1459, 1268, 1225, 1166, 1109, 1073, 1055, 770, 693 cm^−1^. HRMS-FAB: *m*/*z* [M + H]^+^ calcd for C_16_H_21_N_2_O_2_: 273.1603; found: 273.1602.

### General procedure for synthesis of tautomeric 2-aryl-DPs 10 and 11

#### Ethyl 2,4-diphenyl-1,4-dihydropyrimidine-5-carboxylate (10a) and ethyl 2,6-diphenyl-1,6-dihydropyrimidine-5-carboxylate (11a)

To a solution of 7a (334 mg, 0.822 mmol) in CH_2_Cl_2_ (8.0 mL) was added trifluoroacetic acid (2.50 mL, 32.7 mmol) at 0 °C. The reaction mixture was stirred at room temperature for 3 h, and 2 M NaOH aqueous solution (20 mL) and EtOAc (20 mL) were added. The organic layer was separated, and the aqueous layer was extracted with EtOAc (20 mL). The combined organic layers were washed with water (5 mL), brine (5 mL), dried over anhydrous Na_2_SO_4_, filtered, and concentrated under reduced pressure. The residue was purified by flash column chromatography (silica gel; eluent: *n-*hexane–EtOAc–Et_3_N, 150 : 60 : 1 to 100 : 100 : 1) to give a tautomeric mixture of 10a and 11a (249 mg, 0.813 mmol, 99%) as yellow crystals. Mp 152–153 °C (*n*-hexane–EtOAc). ^1^H NMR of the mixture of tautomers, 10a : 11a = 1.6 : 1 (DMSO-*d*_6_, 500 MHz): *δ* = 1.147 (10a, t, *J* = 7.0 Hz, 3H), 1.152 (11a, t, *J* = 7.0 Hz, 3H), 3.98–4.12 (10a, m, 2H + 11a, m, 2H), 5.45 (11a, d, *J* = 3.5 Hz, 1H), 5.57 (10a, s, 1H), 7.16–7.56 (10a, m, 8H + 11a, m, 8H), 7.38 (10a, d, *J* = 5.5 Hz, 1H), 7.66 (11a, s, 1H), 7.80 (10a, d, *J* = 8.5 Hz, 2H), 7.88 (11a, d, *J* = 8.5 Hz, 2H), 9.28 (11a, d, *J* = 3.5 Hz, 1H), 9.88 (10a, d, *J* = 5.5 Hz, 1H). ^1^H NMR, average spectrum of the tautomers (CD_3_OD, 500 MHz): *δ* = 1.21 (t, *J* = 7.0 Hz, 3H), 4.10 (dq, *J* = 10.5, 7.0 Hz, 1H), 4.13 (dq, *J* = 10.5, 7.0 Hz, 1H), 5.58 (s, 1H), 7.25 (t, *J* = 7.5 Hz, 1H), 7.33 (t, *J* = 7.5 Hz, 2H), 7.39 (d, *J* = 7.5 Hz, 2H), 7.45 (t, *J* = 7.5 Hz, 2H), 7.53 (t, *J* = 7.5 Hz, 1H), 7.58 (s, 1H), 7.69 (d, *J* = 7.5 Hz, 2H). ^13^C NMR, average spectrum of the tautomers (CD_3_OD, 125 MHz): *δ* = 14.6, 56.6, 61.3, 107.5 (br), 128.1, 128.3, 128.8, 129.6, 129.8, 132.5, 135.0, 140.7 (br), 146.2, 156.8 (br), 168.0. IR (neat): 2974, 1694, 1684, 1620, 1478, 1393, 1299, 1228, 1095, 756, 713, 698 cm^−1^. HRMS-FAB: *m*/*z* [M + H]^+^ calcd for C_19_H_19_N_2_O_2_: 307.1447; found: 307.1444.

#### Ethyl 2-(4-methoxyphenyl)-4-phenyl-1,4-dihydropyrimidine-5-carboxylate (10b) and ethyl 2-(4-methoxyphenyl)-6-phenyl-1,6-dihydropyrimidine-5-carboxylate (11b)

Eluent in chromatography: *n-*hexane–EtOAc–Et_3_N, 100 : 100 : 1 to 75 : 150 : 1. Yield: 98%; pale yellow amorphous. ^1^H NMR of the mixture of tautomers, 10b : 11b = 1 : 1 (DMSO-*d*_6_, 500 MHz): *δ* = 1.11–1.18 (10b, t, *J* = 7.0 Hz, 3H + 11b, t, *J* = 7.0 Hz, 3H), 3.76–3.81 (10b, s, 3H + 11b, s, 3H), 3.97–4.12 (10b, m, 2H + 11b, m, 2H), 5.41 (11b, d, *J* = 3.5 Hz, 1H), 5.54 (10b, s, 1H), 6.95–7.90 (10b, m, 9H + 11b, m, 9H), 7.37 (10b, d, *J* = 5.5 Hz, 1H), 7.64 (10b, s, 1H), 9.16 (11b, d, *J* = 3.5 Hz, 1H), 9.79 (10b, d, *J* = 5.5 Hz, 1H). ^1^H NMR, average spectrum of the tautomers (CD_3_OD, 500 MHz): *δ* = 1.21 (t, *J* = 7.0 Hz, 3H), 3.83 (s, 3H), 4.10 (dq, *J* = 10.5, 7.0 Hz, 1H), 4.13 (dq, *J* = 10.5, 7.0 Hz, 1H), 5.55 (s, 1H), 6.98 (d, *J* = 8.5 Hz, 2H), 7.25 (t, *J* = 7.0 Hz, 1H), 7.32 (t, *J* = 7.0 Hz, 2H), 7.37 (d, *J* = 7.0 Hz, 2H), 7.60 (s, 1H), 7.66 (d, *J* = 8.5 Hz, 2H). ^13^C NMR, average spectrum of the tautomers (CD_3_OD, 125 MHz): *δ* = 14.6, 55.9, 56.1, 61.2, 107.7 (br), 115.0, 126.9, 128.0, 128.8, 129.5, 130.1, 142.1 (br), 146.3, 157.2 (br), 164.0, 168.0. IR (neat): 1691, 1670, 1605, 1480, 1251, 1225, 1173, 1097, 1075, 1029, 838, 754, 697 cm^−1^. HRMS-FAB: *m*/*z* [M + H]^+^ calcd for C_20_H_21_N_2_O_3_: 337.1552; found: 337.1568.

#### Ethyl 2-(4-nitrophenyl)-4-phenyl-1,4-dihydropyrimidine-5-carboxylate (10g) and ethyl 2-(4-nitrophenyl)-6-phenyl-1,6-dihydropyrimidine-5-carboxylate (11g)

Eluent in chromatography: *n-*hexane–EtOAc–Et_3_N, 150 : 100 : 1 to 100 : 100 : 1. Yield: 97%; orange amorphous. ^1^H NMR of the mixture of tautomers, 10g : 11g = 2.5 : 1 (DMSO-*d*_6_, 500 MHz): *δ* = 1.14 (11g, t, *J* = 7.0 Hz, 3H), 1.16 (11g, t, *J* = 7.0 Hz, 3H), 3.97–4.12 (10g, m, 2H + 11g, m, 2H), 5.49 (11g, d, *J* = 3.0 Hz, 1H), 5.62 (10g, s, 1H), 7.16–7.44 (10g, m, 5H + 11g, m, 5H), 7.41 (10g, d, *J* = 5.0 Hz, 1H), 7.67 (11g, s, 1H), 8.05 (10g, d, *J* = 8.5 Hz, 2H), 8.12 (11g, d, *J* = 8.5 Hz, 2H), 8.30 (10g, d, *J* = 8.5 Hz, 2H), 8.32 (11g, d, *J* = 8.5 Hz, 2H), 9.54 (11g, d, *J* = 3.0 Hz, 1H), 10.15 (10g, d, *J* = 5.0 Hz, 1H). ^1^H NMR, average spectrum of the tautomers (CD_3_OD, 500 MHz): *δ* = 1.21 (t, *J* = 7.0 Hz, 3H), 4.10 (dq, *J* = 10.5, 7.0 Hz, 1H), 4.13 (dq, *J* = 10.5, 7.0 Hz, 1H), 5.63 (s, 1H), 7.26 (t, *J* = 7.5 Hz, 1H), 7.34 (t, *J* = 7.5 Hz, 2H), 7.40 (d, *J* = 7.5 Hz, 2H), 7.44–7.70 (brs, 1H), 7.92 (d, *J* = 9.0 Hz, 2H), 8.30 (d, *J* = 9.0 Hz, 2H). ^13^C NMR, average spectrum of the tautomers (CD_3_OD, 125 MHz): *δ* = 14.5, 57.4, 61.4, 105.5–108.5 (br), 124.7, 128.2, 128.9, 129.5, 129.7, 137.0–141.0 (br), 140.8, 146.1, 150.8, 153.0–156.0 (br), 167.7. IR (neat): 1695, 1674, 1600, 1521, 1487, 1344, 1297, 1242, 1190, 1097, 1072, 851, 752, 698 cm^−1^. HRMS-FAB: *m*/*z* [M + H]^+^ calcd for C_19_H_18_N_3_O_4_: 352.1297; found: 352.1305.

## Conflicts of interest

The authors declare no conflict of interest.

## Supplementary Material

RA-012-D2RA05155A-s001
