# Supplementary material for: Palladium-catalyzed/copper-mediated carbon–carbon cross-coupling reaction for synthesis of 6-unsubstituted 2-aryldihydropyrimidines
Source: RSC Adv. 2022 Oct 3;12(43):28113–22. doi: 10.1039/d2ra05155a (PMC9527642; doi:10.1039/d2ra05155a)

Electronic Supplementary Material (ESI) for RSC Advances.
This journal is © The Royal Society of Chemistry 2022

# Supplementary information

## Palladium-catalyzed/copper-mediated carbon-carbon cross-coupling reaction for synthesis of 6-unsubstituted 2-aryldihydropyrimidines

Yoshio Nishimura,[*,a,b] Takanori Kubo,[b] Saho Takayama,[b] Hanako Yoshida,[b] and Hidetsura Cho[c]

[a]*School of Pharmaceutical Sciences, Ohu University; 31-1 Misumido, Tomita-machi, Koriyama, Fukushima 963-8611, Japan.*

[b]*Faculty of Pharmacy, Yasuda Women's University, 6-13-1, Yasuhigashi, Asaminami-ku, Hiroshima 731-0153, Japan.*

[c]*Graduate School of Pharmaceutical Sciences, Tohoku University, 6-3 Aoba, Aramaki, Aoba-ku, Sendai 980-8578, Japan.*

**Table of Contents**

$^{1}H$ and $^{13}C$ spectra


Compound **6b** ........ 4

Compound **6c** ........ 5

Compound **6d** ........ 6

Compound **6e** ........ 7

Compound **6f** ........ 8

Compound **6g** ........ 9

Compound **6i** ........ 10

Compound **7a** ........ 11

Compound **7b** ........ 12

Compound **7c** ........ 13

Compound **7d** ........ 14

Compound **7e** ........ 15

Compound **7f** ........ 16

Compound **7g** ........ 17

Compound **7h** ........ 18

Compound **7i** ........ 19

Compound **7j** ........ 20

Compound **7k** ........ 21

Compound **7l** ........ 22

Compound **7m** ........ 23

Compound **7n** ........ 24

Compound **7o** ........ 25

Compound **7p** ........ 26

Compound **7q** ........ 27

Compound **7r** ........ 28

Compound **7s** ........ 29

Compound **7t** ........ 30

Compound **7u** ........ 31

Compound **9** ........ 32

Compounds **10a** and **11a** ........ 34

Compounds **10b** and **11b** ........ 37

Compounds **10g** and **11g** ........ 40

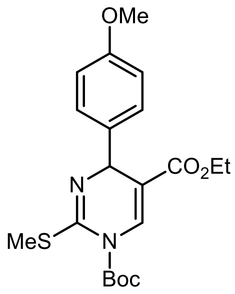


**6b**

$^{1}$H NMR (600 MHz, $CDCl_3$) spectrum of **6b**.

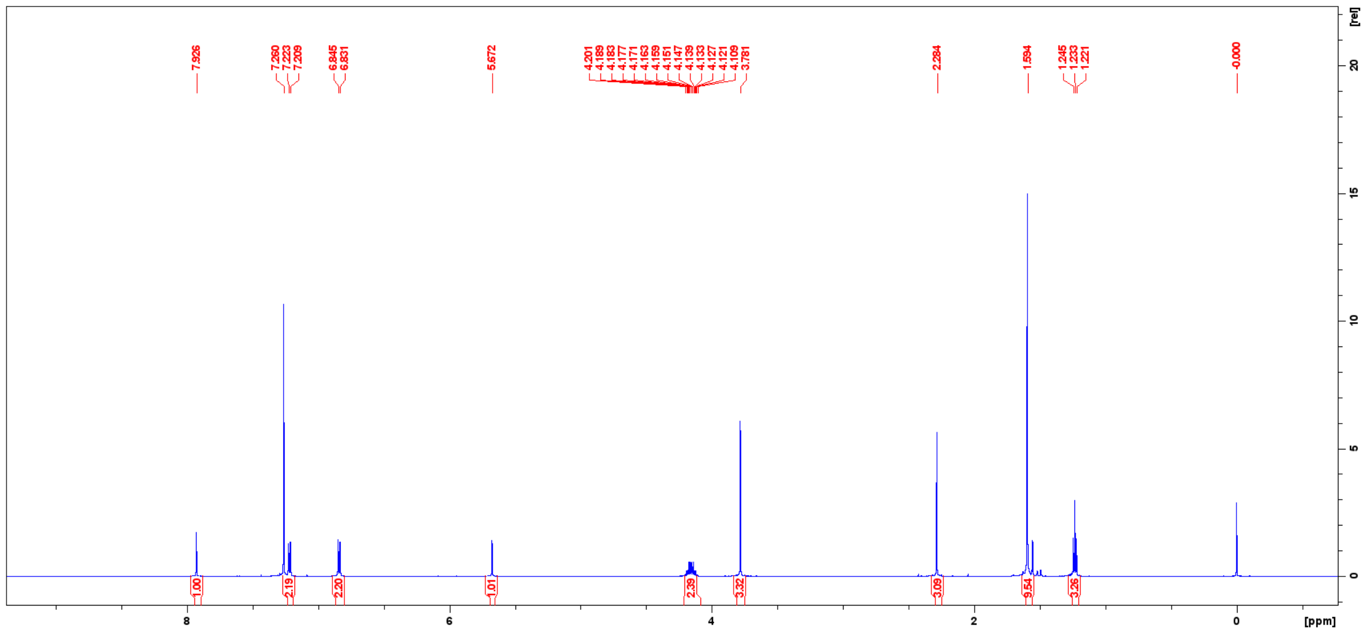


$^{13}$C NMR (150 MHz, $CDCl_3$) spectrum of **6b**.

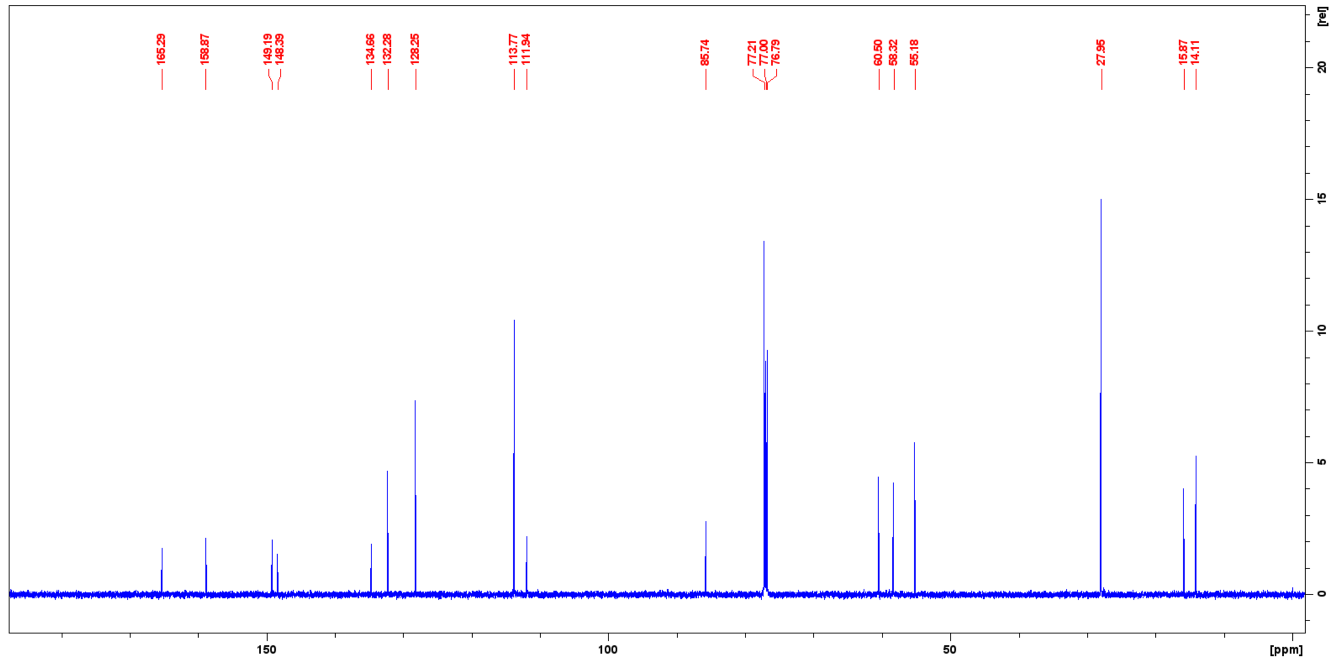

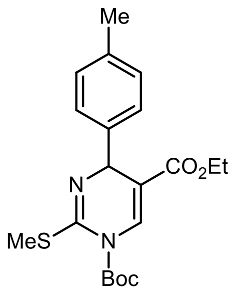


**6c**

$^{1}$H NMR (600 MHz, $CDCl_3$) spectrum of **6c**.

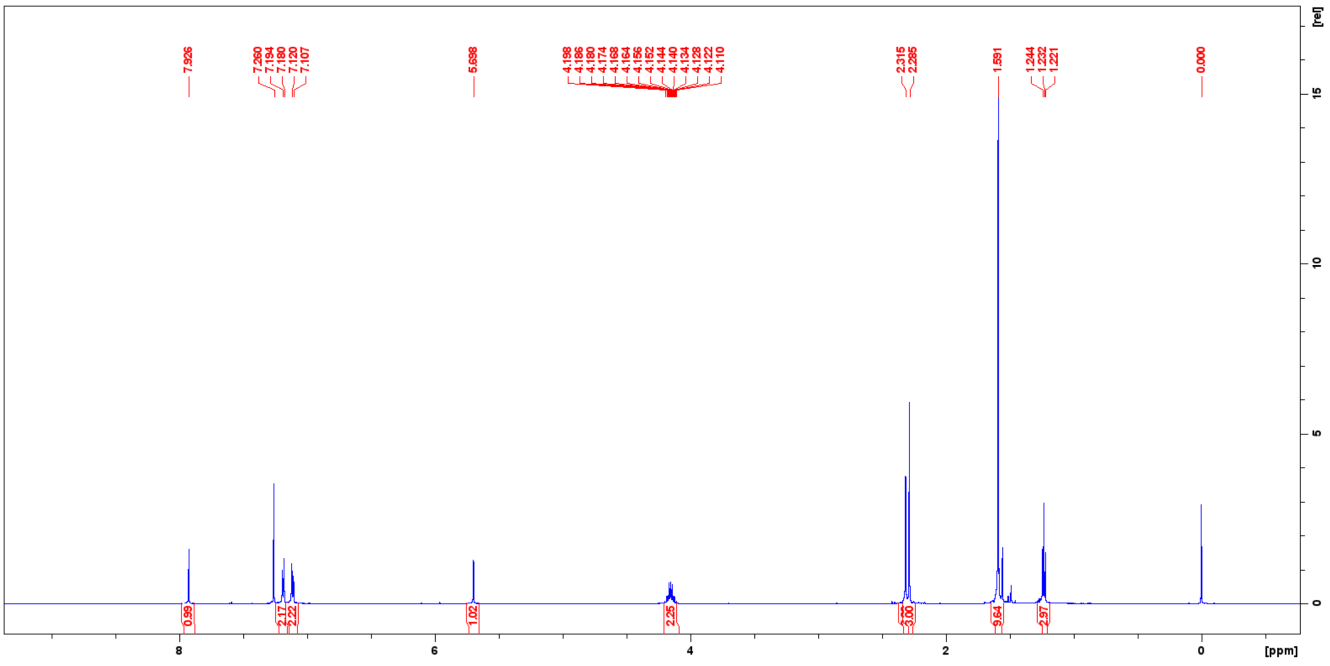

$^{13}$C NMR (150 MHz, $CDCl_3$) spectrum of **6c**.

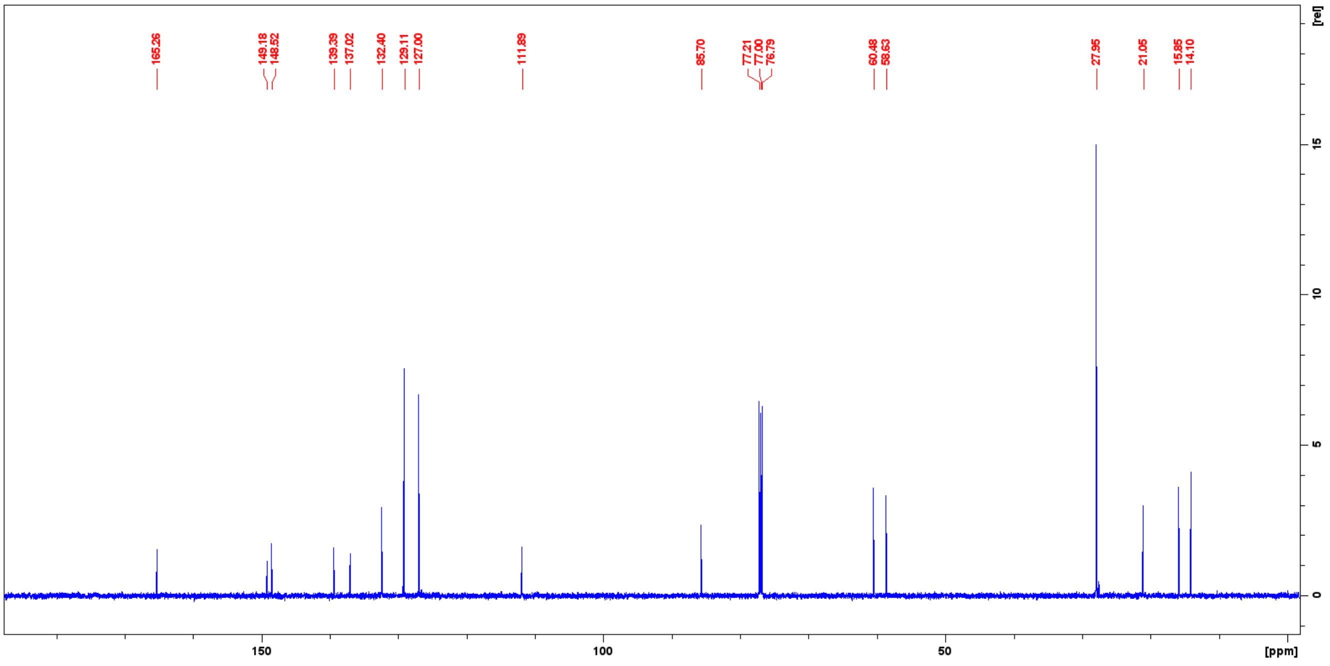

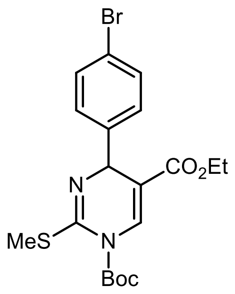


**6d**

$^{1}$H NMR (600 MHz, $CDCl_3$) spectrum of **6d**.

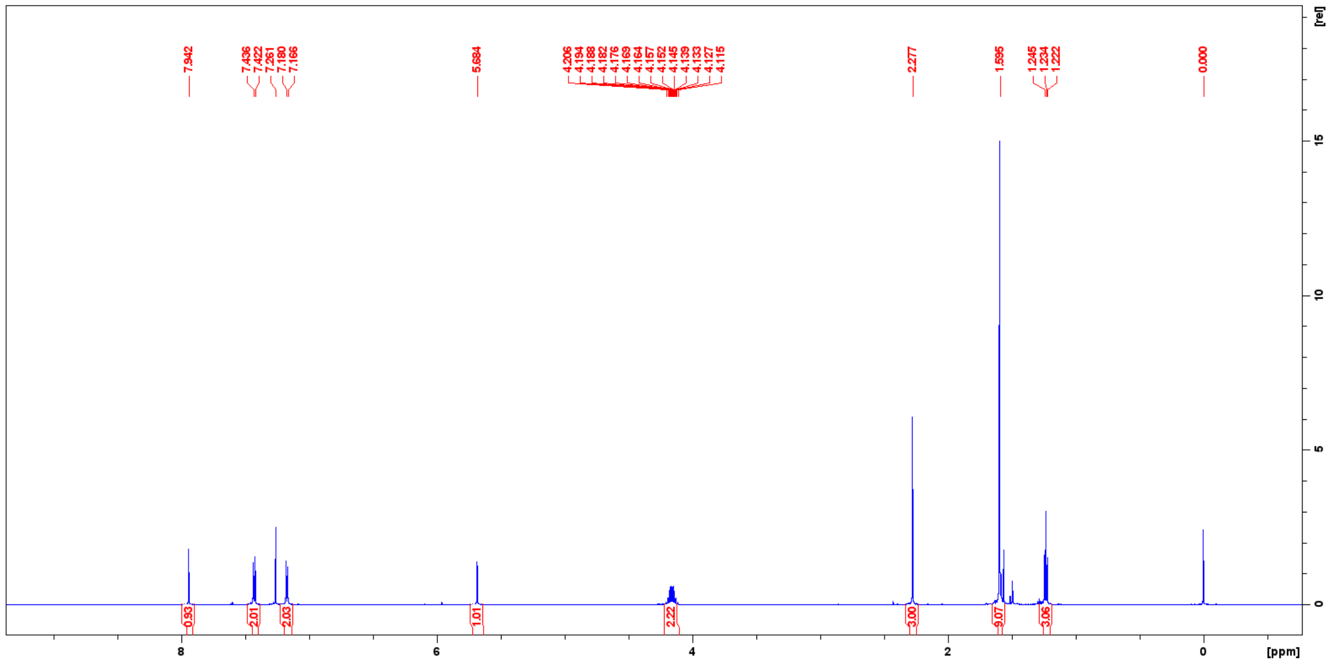


$^{13}$C NMR (150 MHz, DMSO-$d_6$) spectrum of **6d**.

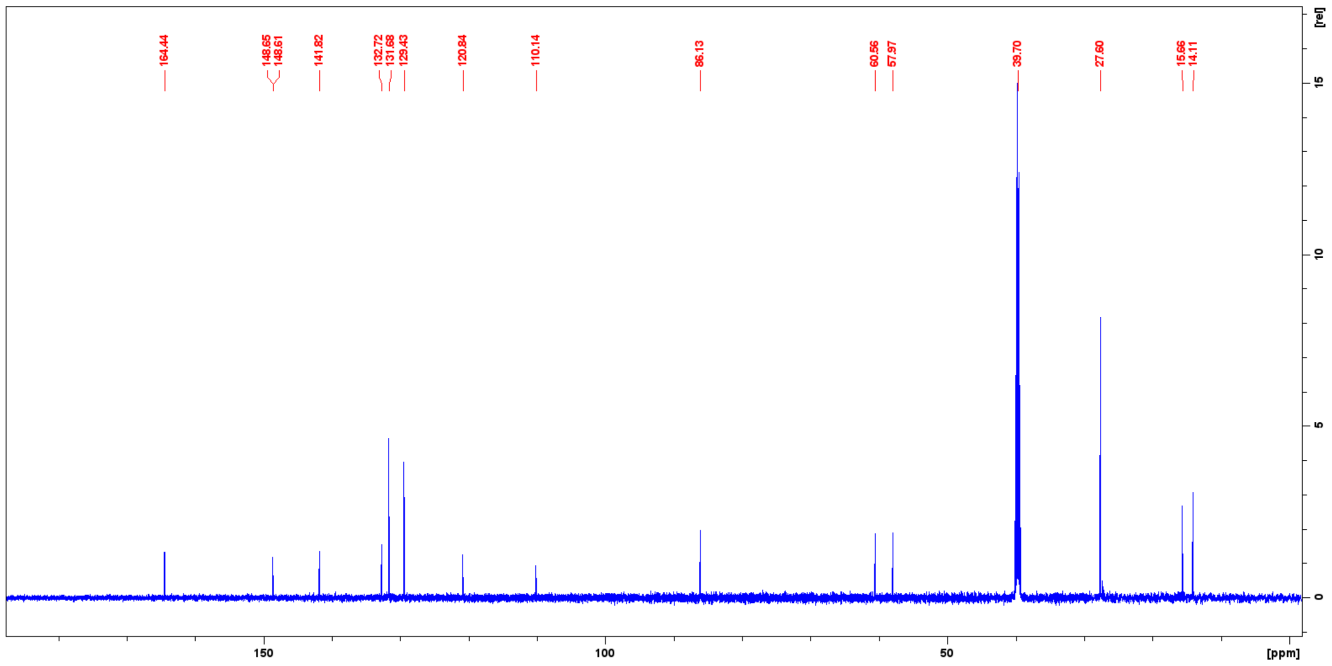

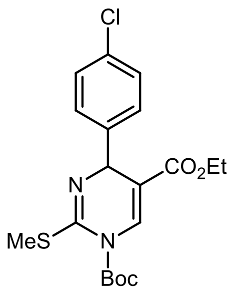


**6e**

$^{1}$H NMR (600 MHz, $CDCl_3$) spectrum of **6e**.

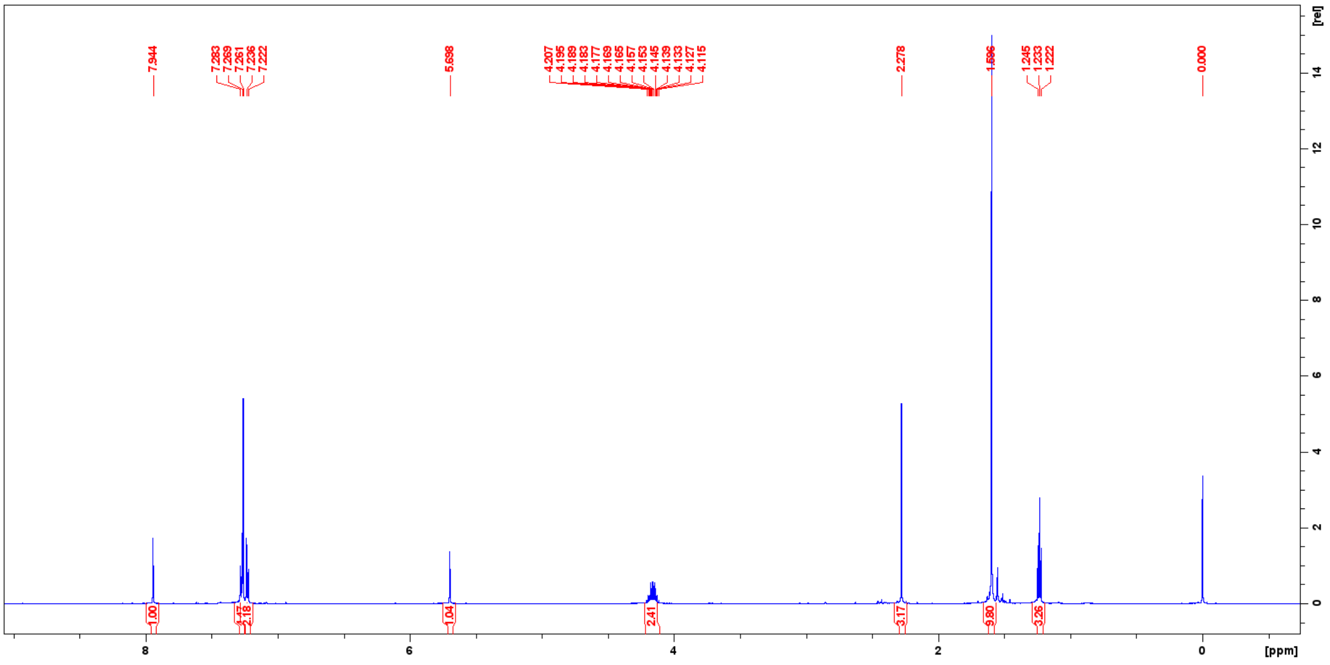


$^{13}$C NMR (150 MHz, $CDCl_3$) spectrum of **6e**.

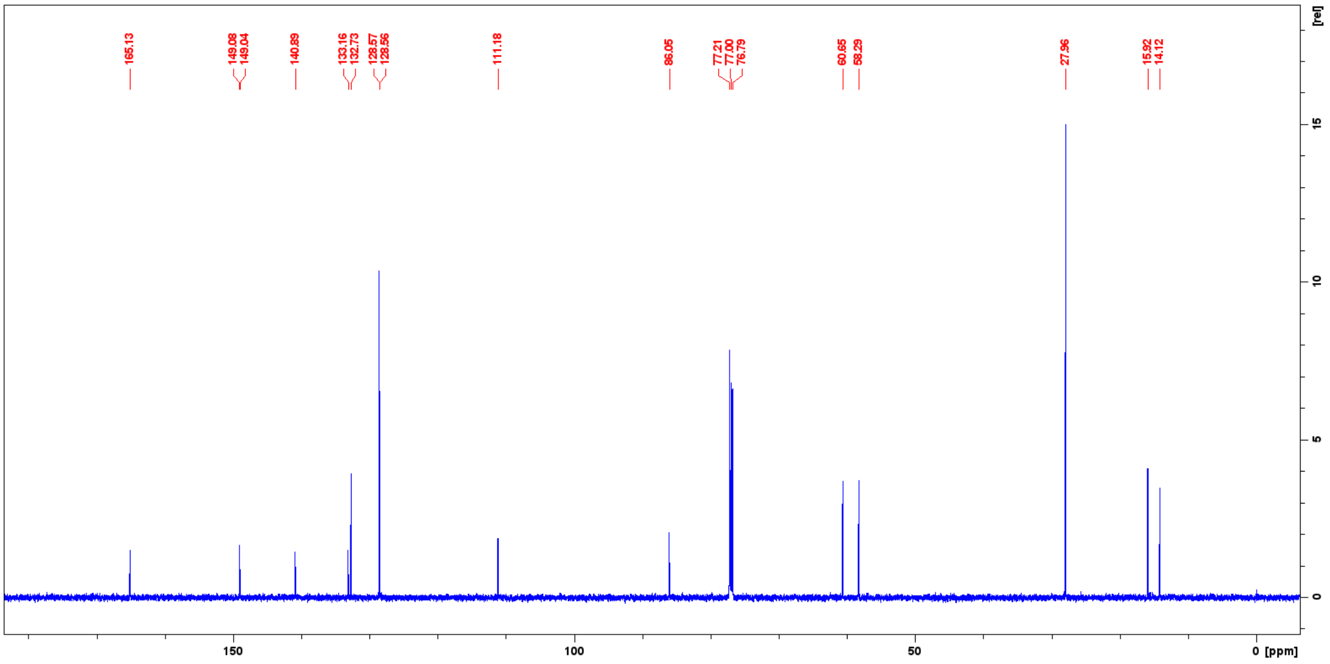

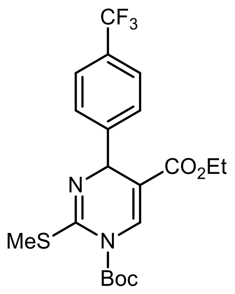


**6f**

$^{1}$H NMR (600 MHz, $CDCl_3$) spectrum of **6f**.

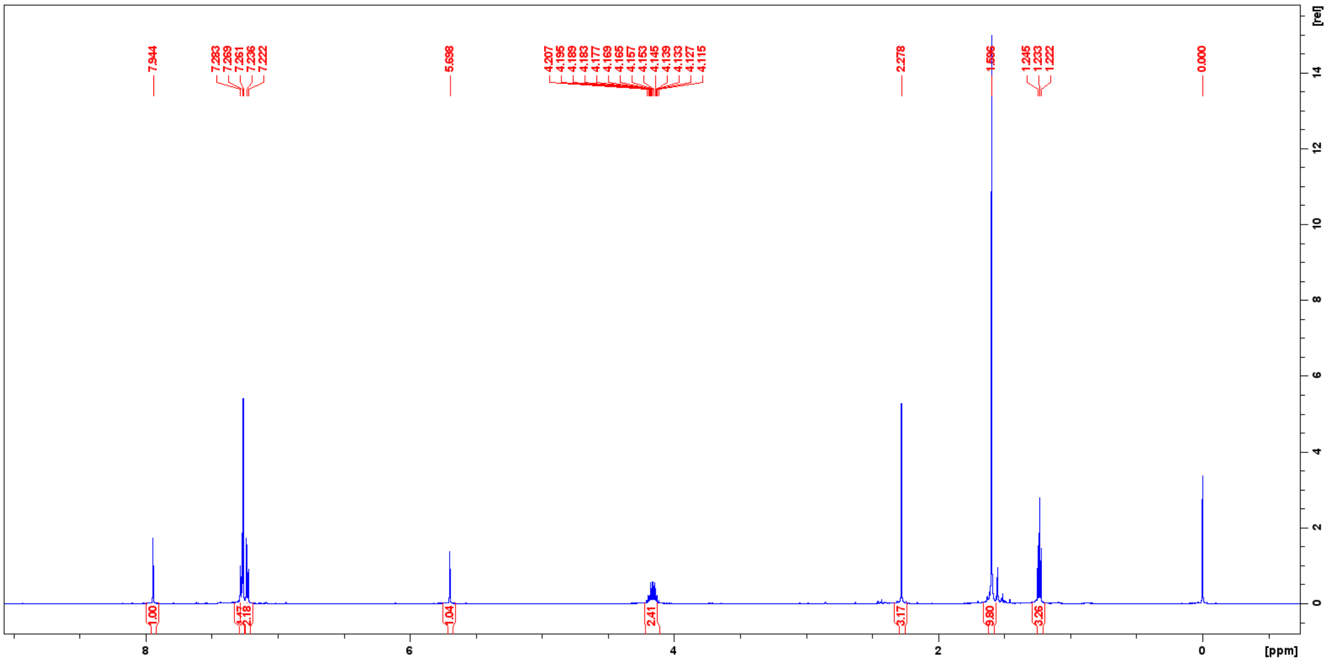


$^{13}$C NMR (150 MHz, $CDCl_3$) spectrum of **6f**.

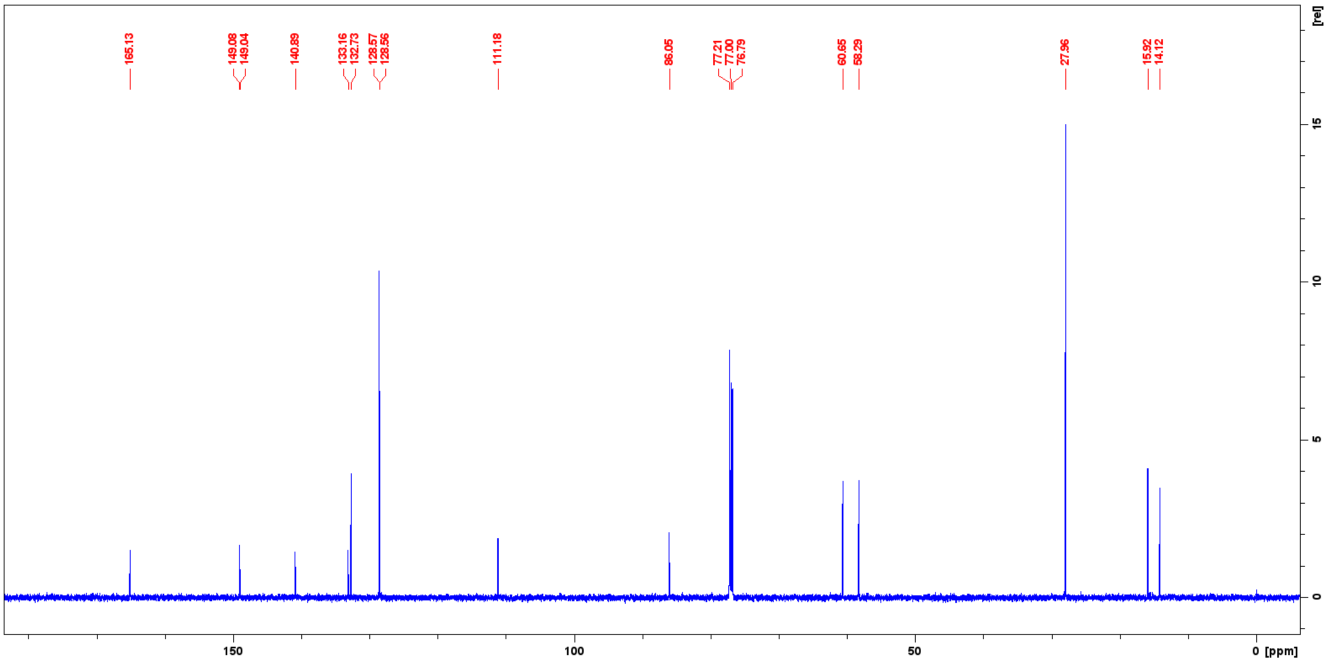

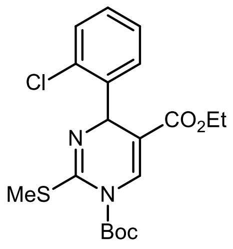


**6g**

$^{1}$H NMR (600 MHz, CDCl$_3$) spectrum of **6g**.

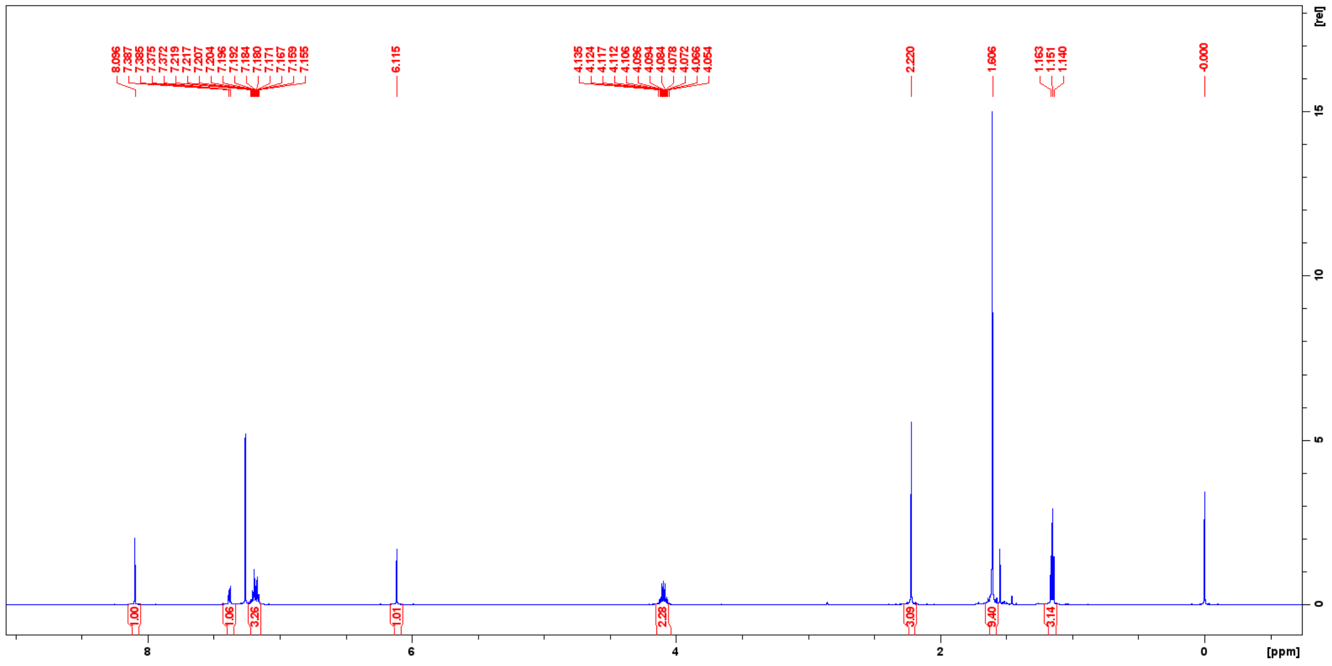


$^{13}$C NMR (150 MHz, CDCl$_3$) spectrum of **6g**.

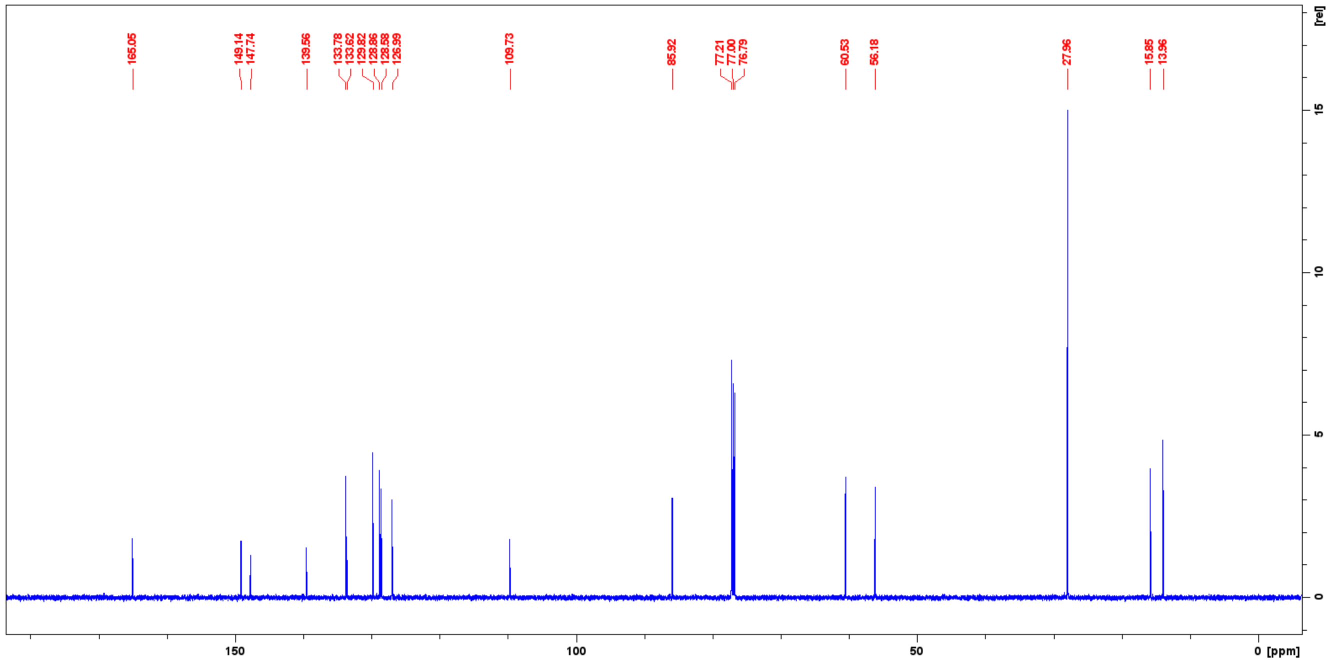

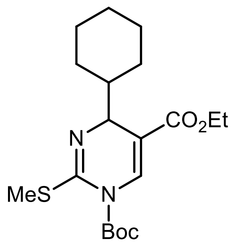


**6i**

$^{1}$H NMR (600 MHz, $CDCl_3$) spectrum of **6i**.

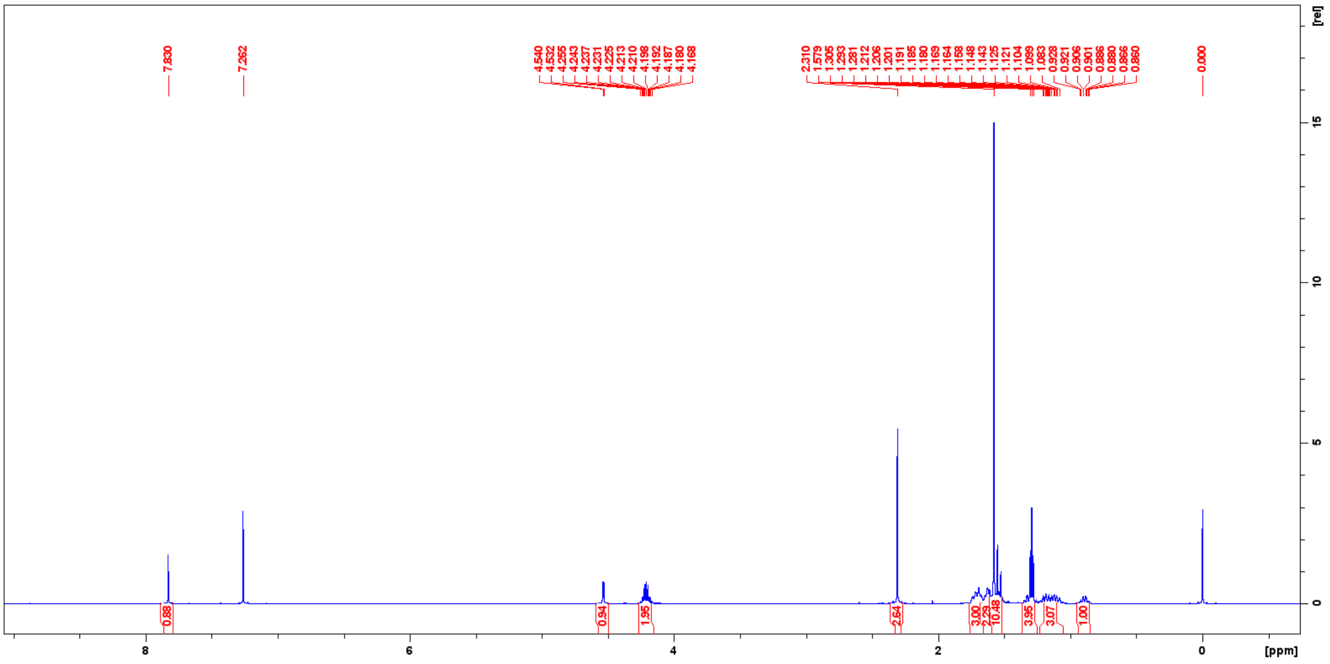


$^{13}$C NMR (150 MHz, $CDCl_3$) spectrum of **6i**.

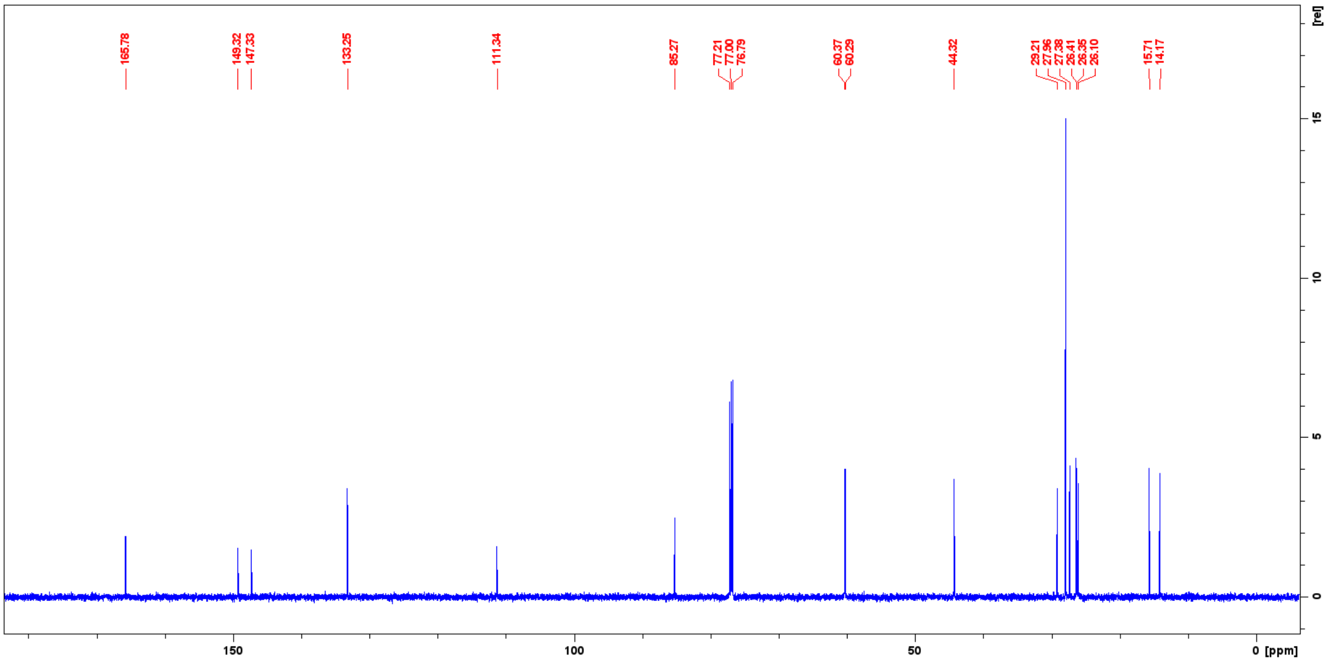

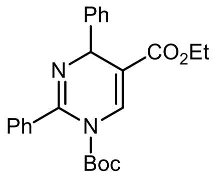


**7a**

$^{1}$H NMR (600 MHz, $CDCl_3$) spectrum of **7a**.

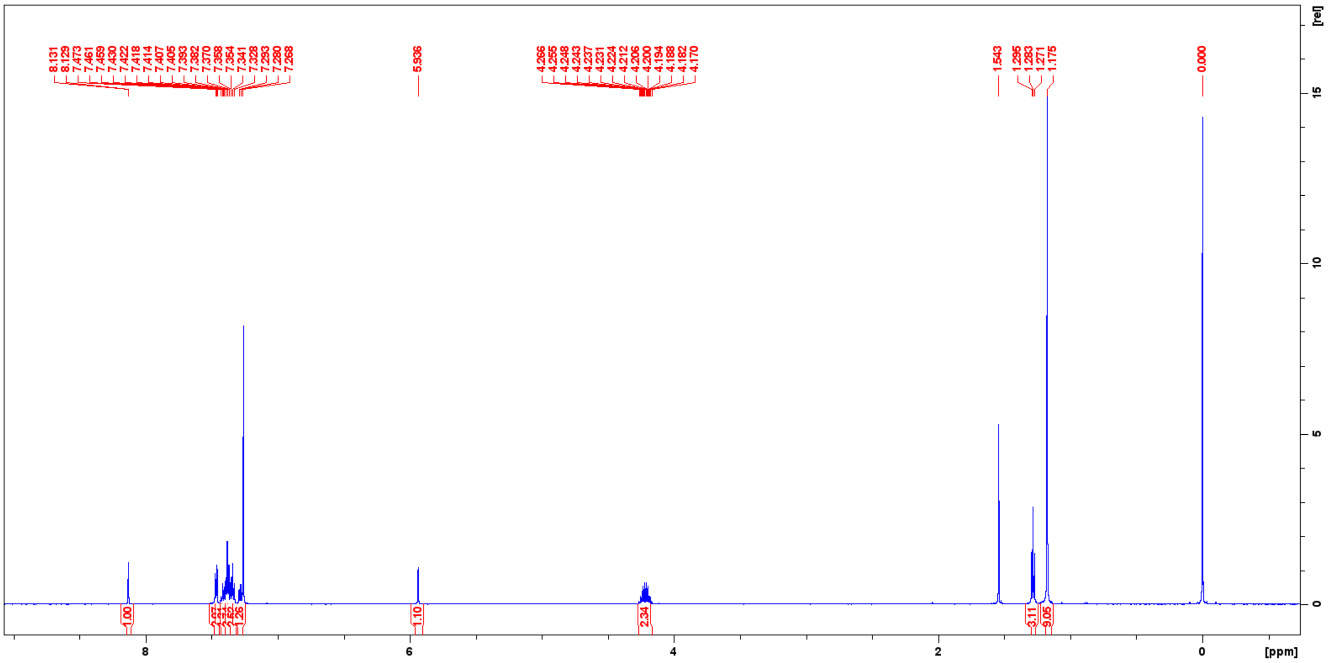


$^{13}$C NMR (150 MHz, $CDCl_3$) spectrum of **7a**.

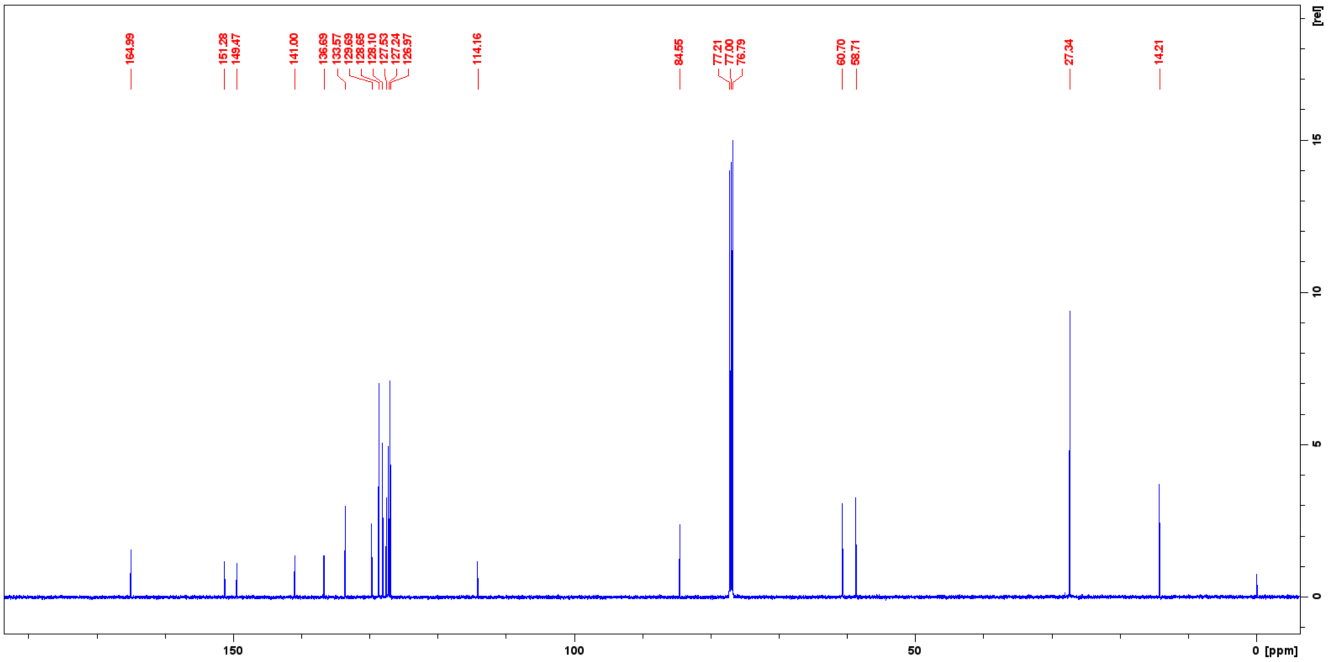

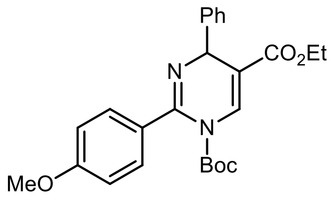


**7b**

$^{1}$H NMR (600 MHz, $CDCl_3$) spectrum of **7b**.

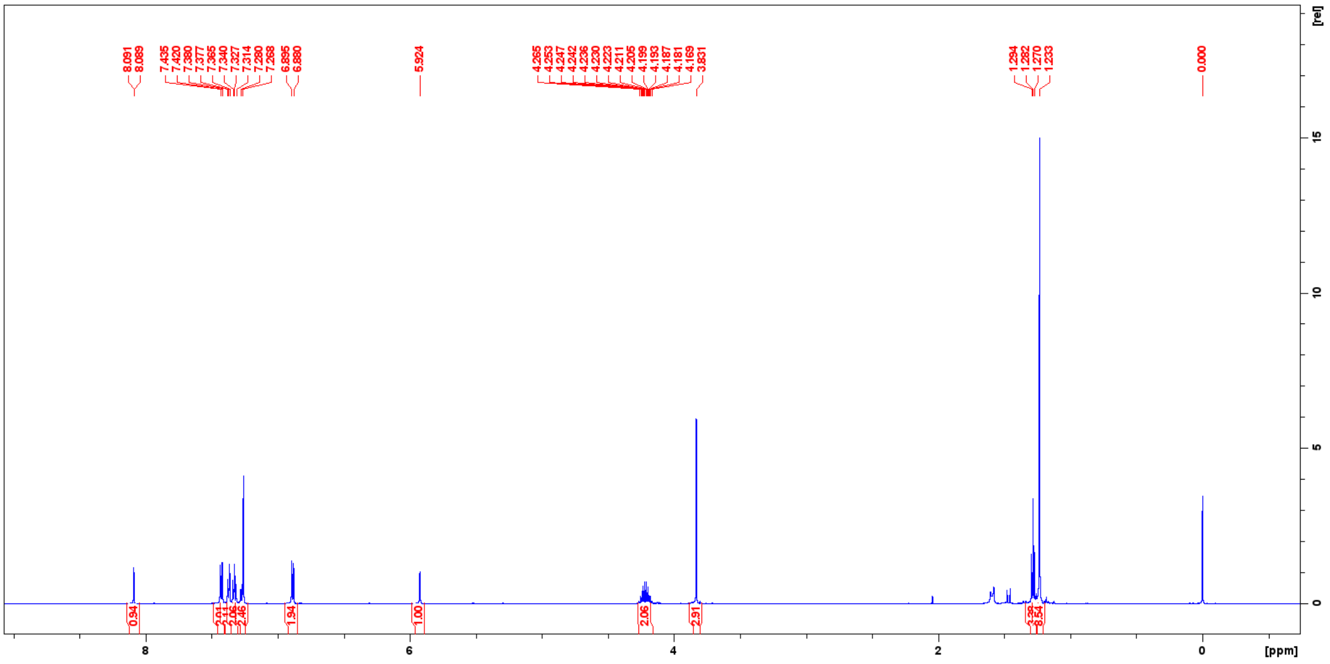


$^{13}$C NMR (150 MHz, $CDCl_3$) spectrum of **7b**.

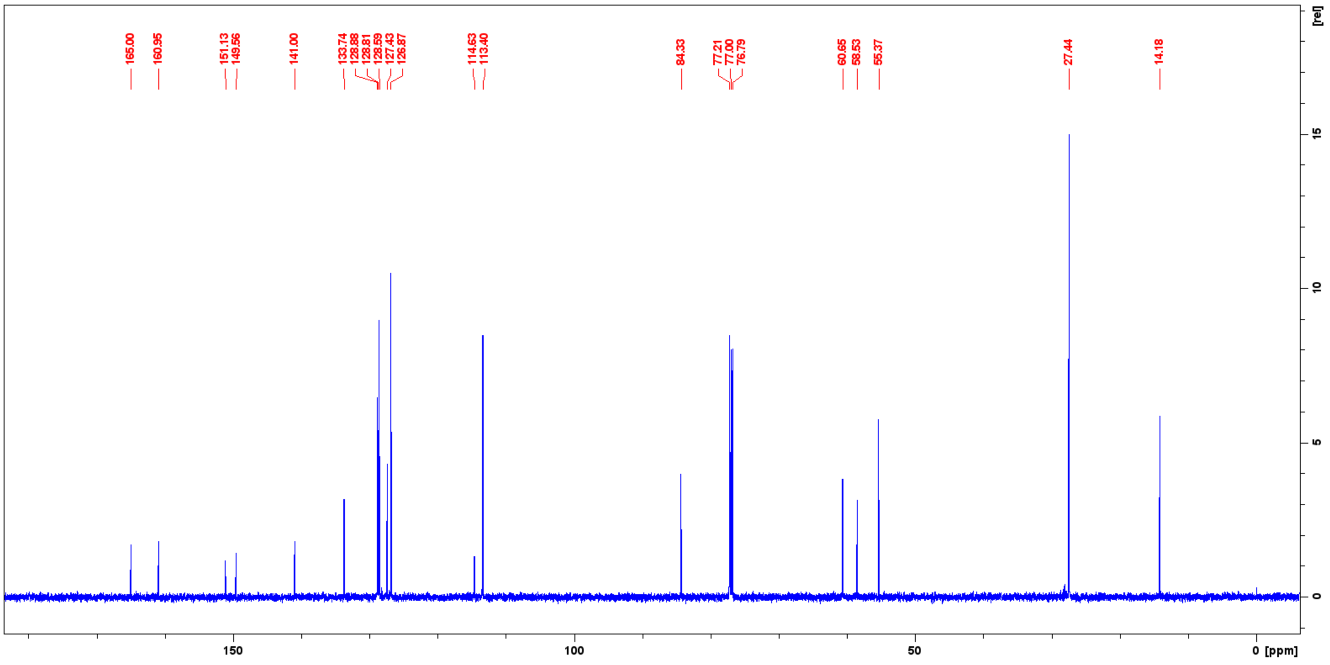

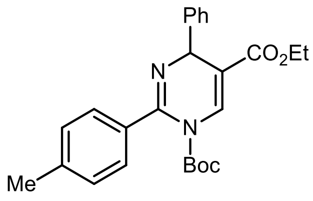


**7c**

$^{1}$H NMR (600 MHz, $CDCl_3$) spectrum of **7c**.

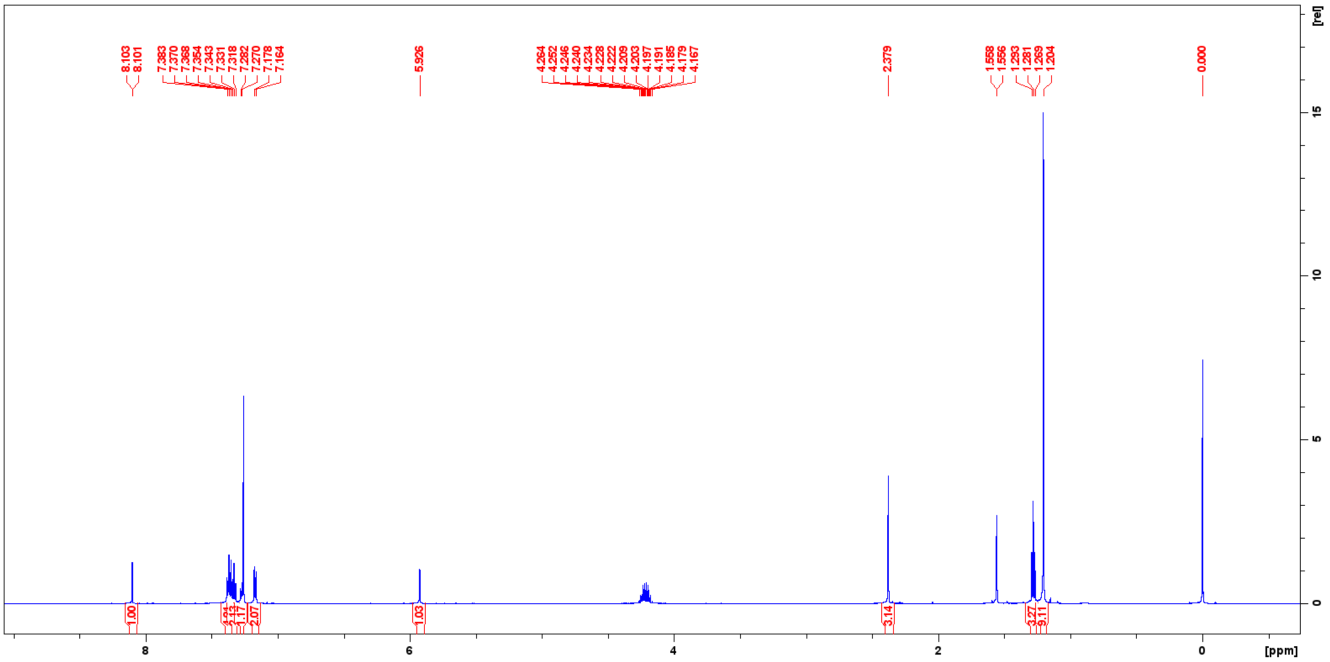


$^{13}$C NMR (150 MHz, $CDCl_3$) spectrum of **7c**.

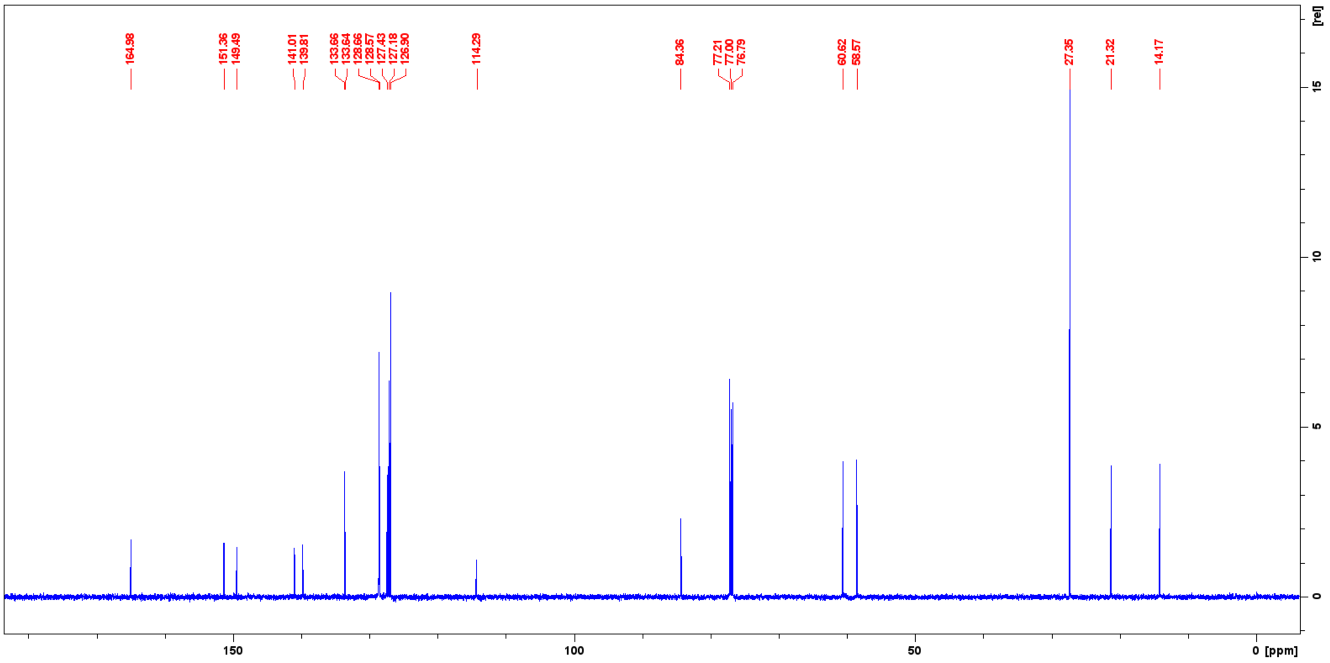

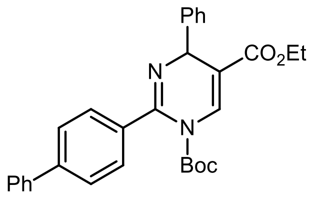


**7d**

$^{1}$H NMR (600 MHz, $CDCl_3$) spectrum of **7d**.

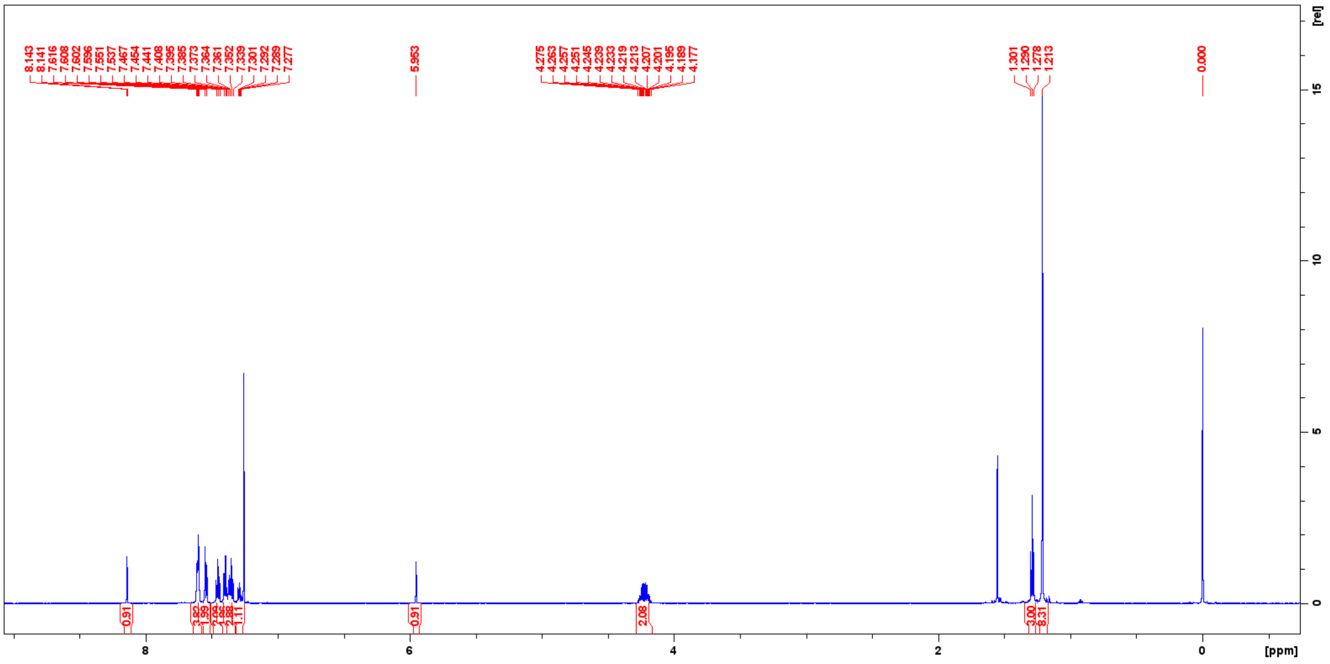


$^{13}$C NMR (150 MHz, $CDCl_3$) spectrum of **7d**.

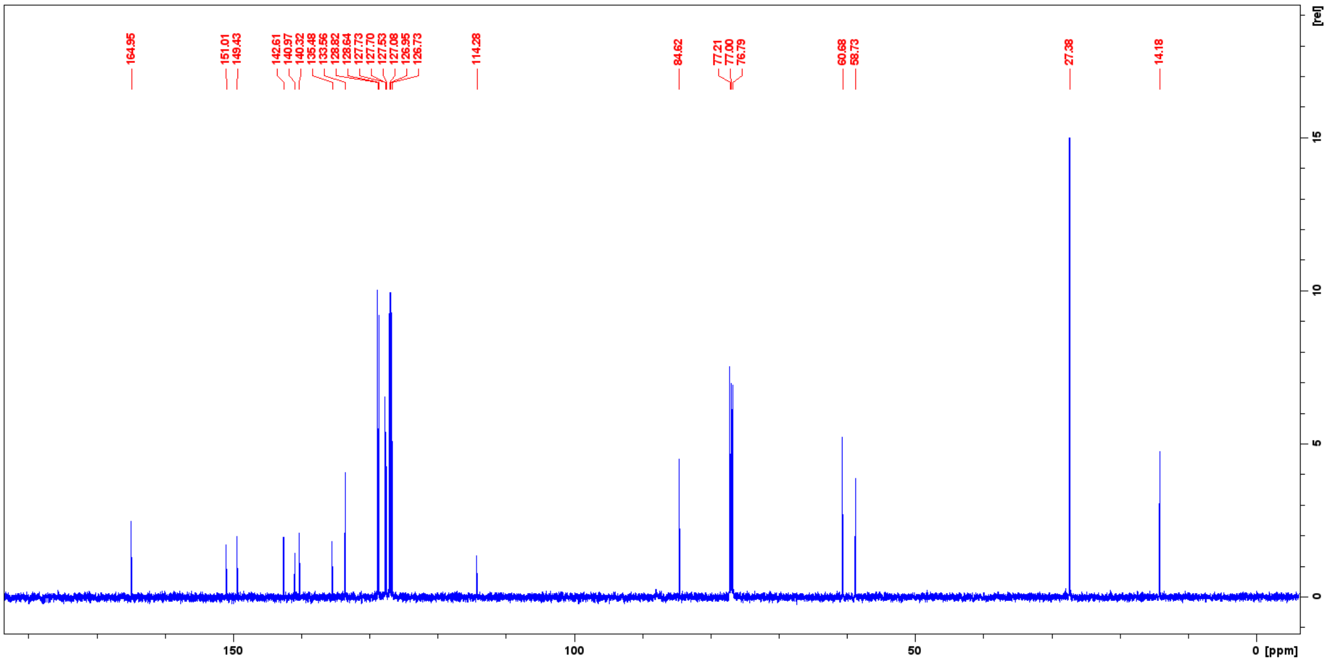

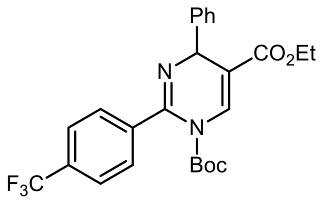


**7e**

$^{1}$H NMR (600 MHz, $CDCl_3$) spectrum of **7e**.

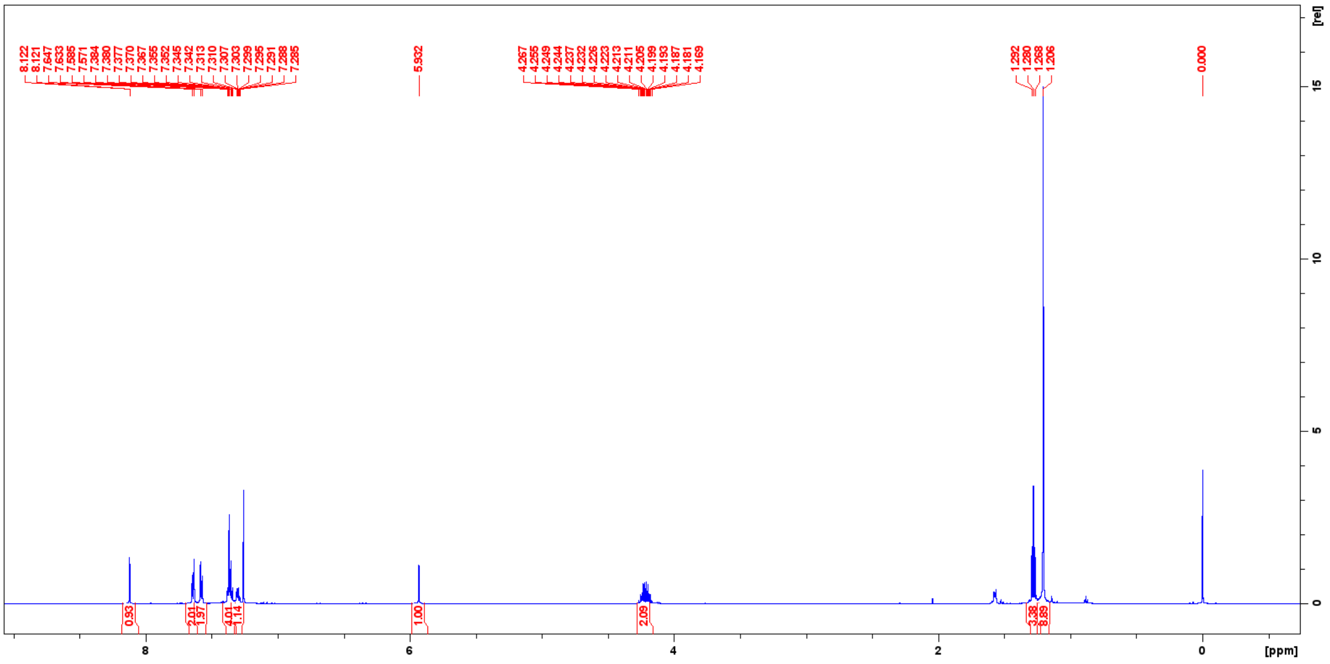


$^{13}$C NMR (150 MHz, $CDCl_3$) spectrum of **7e**.

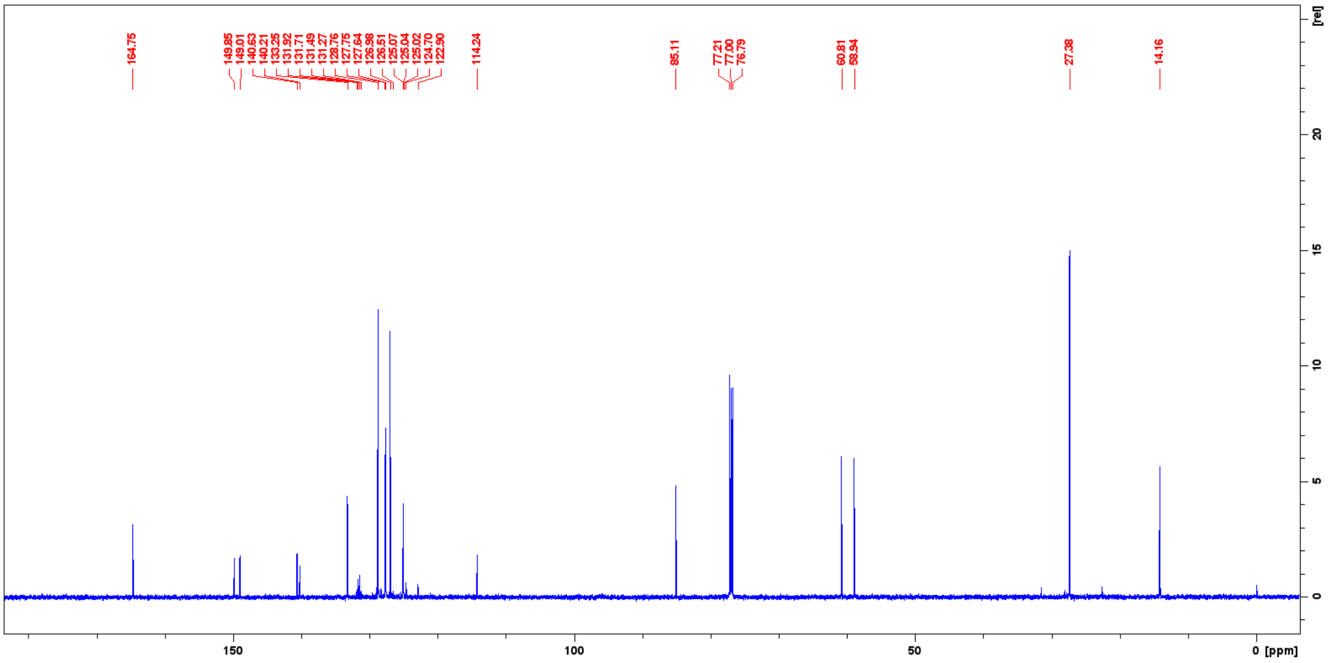

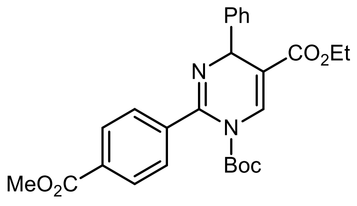


**7f**

$^{1}$H NMR (600 MHz, $CDCl_3$) spectrum of **7f**.

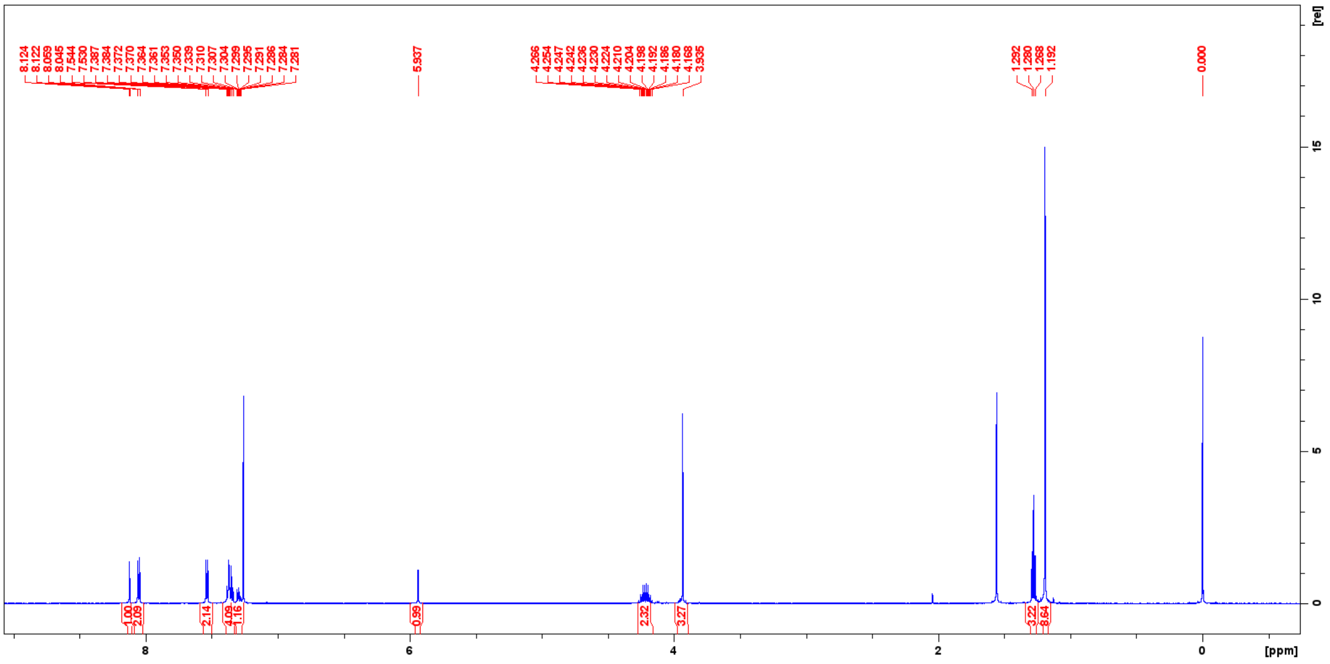


$^{13}$C NMR (150 MHz, $CDCl_3$) spectrum of **7f**.

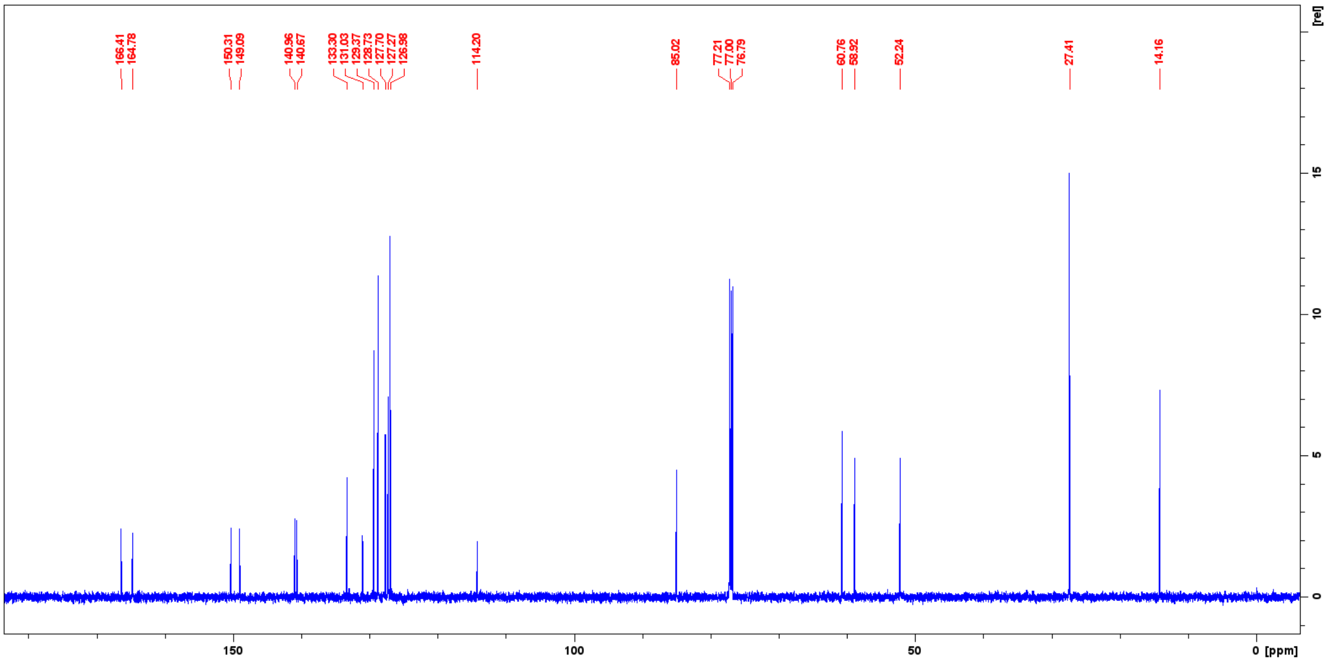

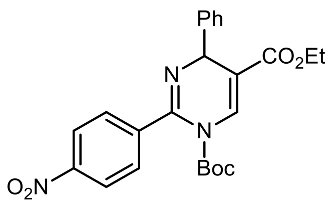


**7g**

$^{1}$H NMR (600 MHz, $CDCl_3$) spectrum of **7g**.

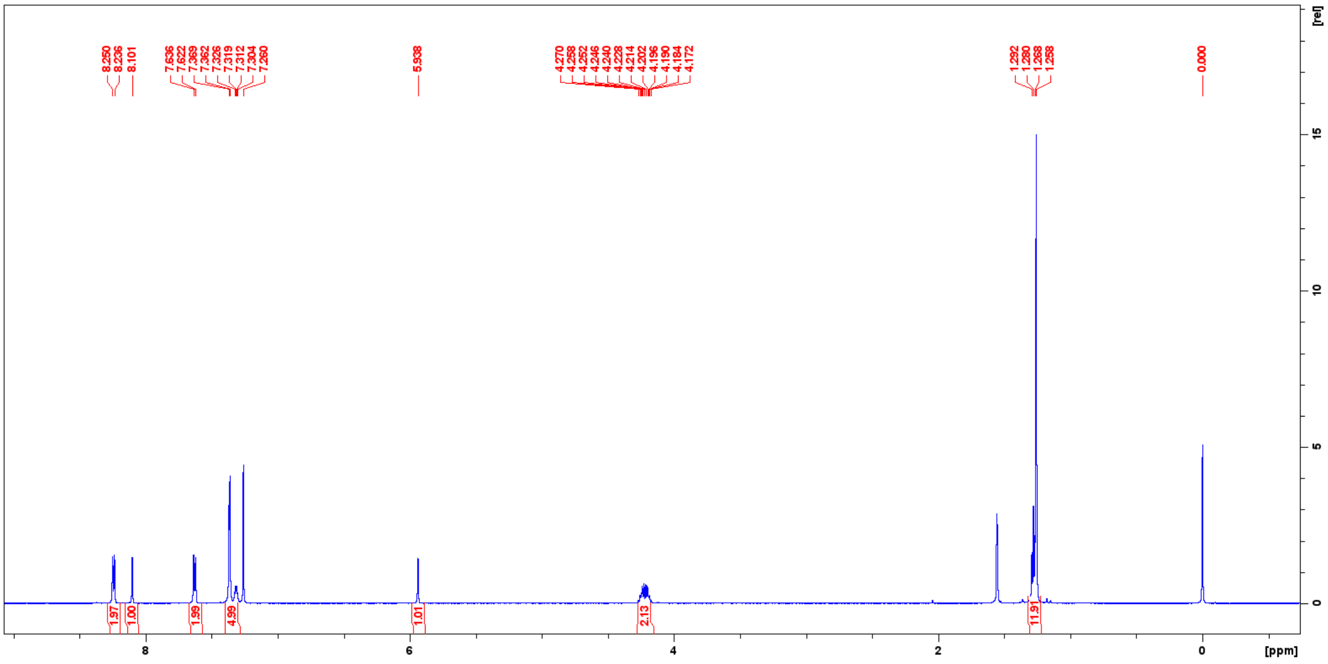


$^{13}$C NMR (150 MHz, $CDCl_3$) spectrum of **7g**.

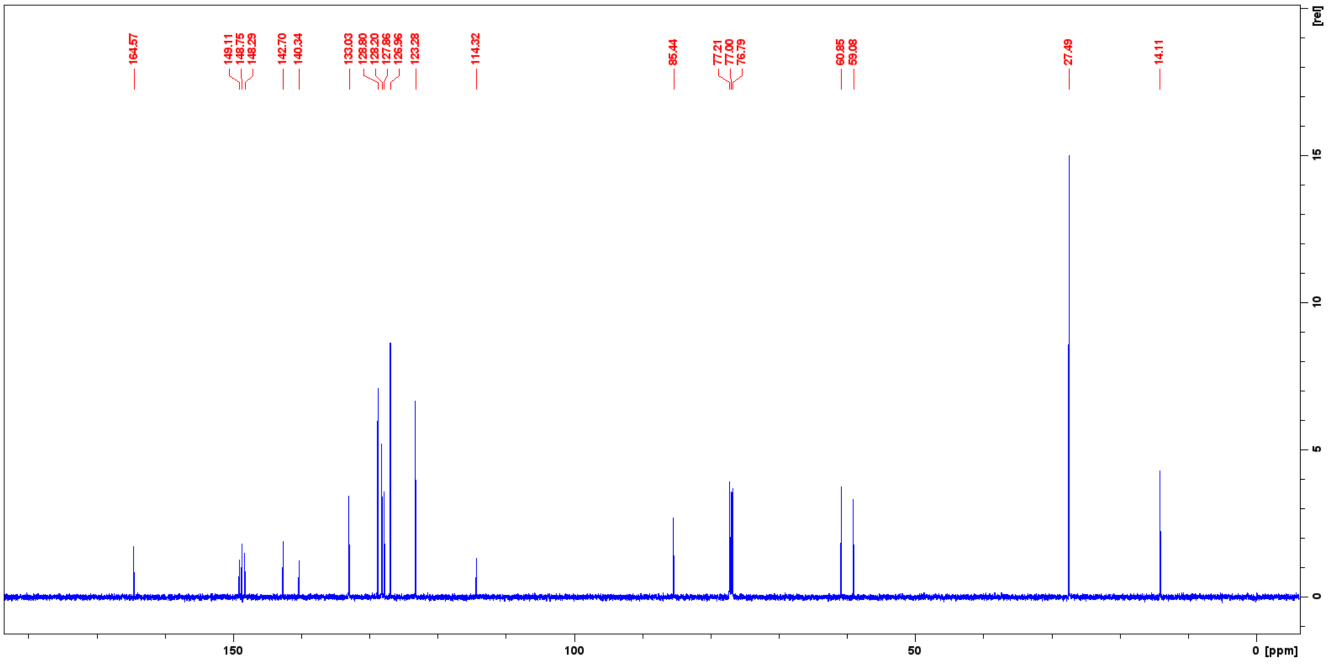

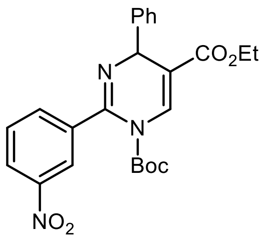


**7h**

$^{1}$H NMR (600 MHz, $CDCl_3$) spectrum of **7h**.

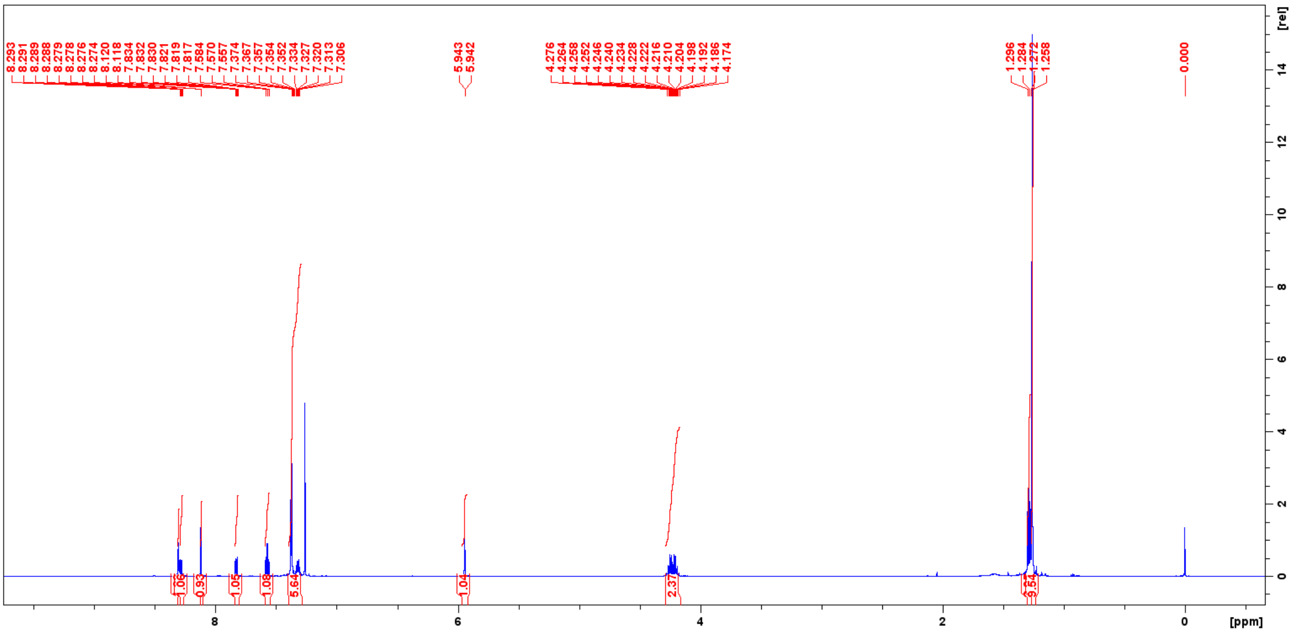


$^{13}$C NMR (150 MHz, $CDCl_3$) spectrum of **7h**.

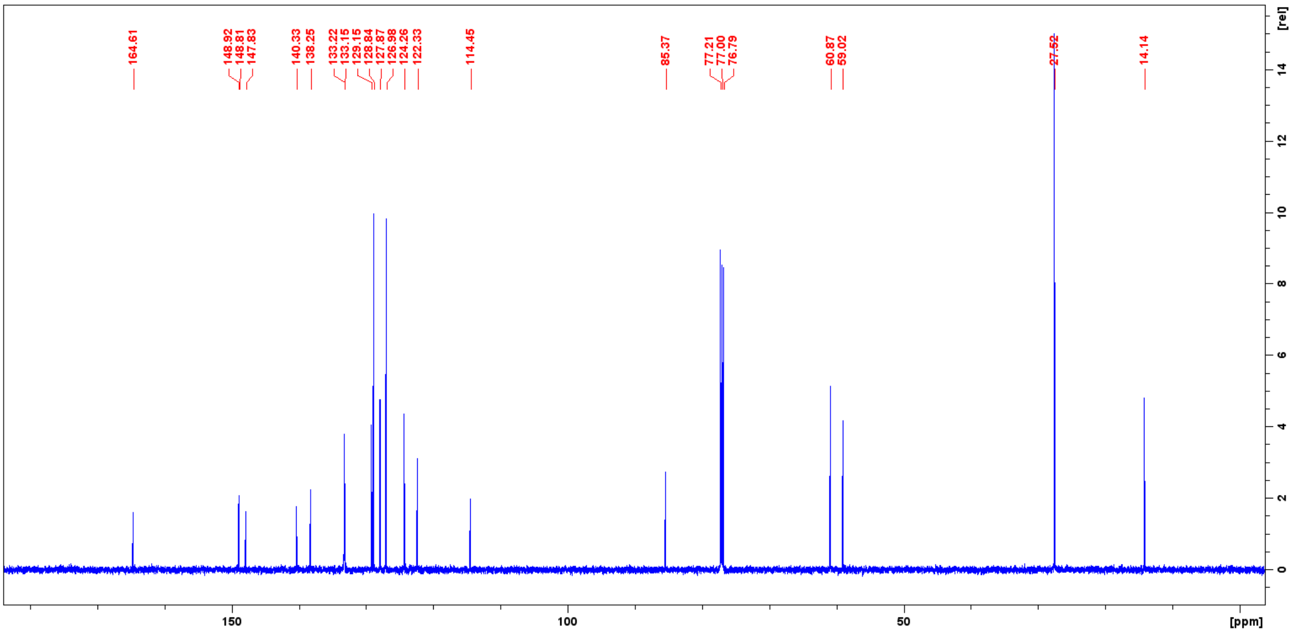

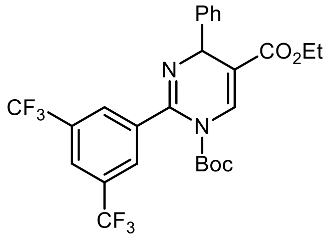


**7i**

$^{1}$H NMR (600 MHz, CDCl3) spectrum of **7i**.

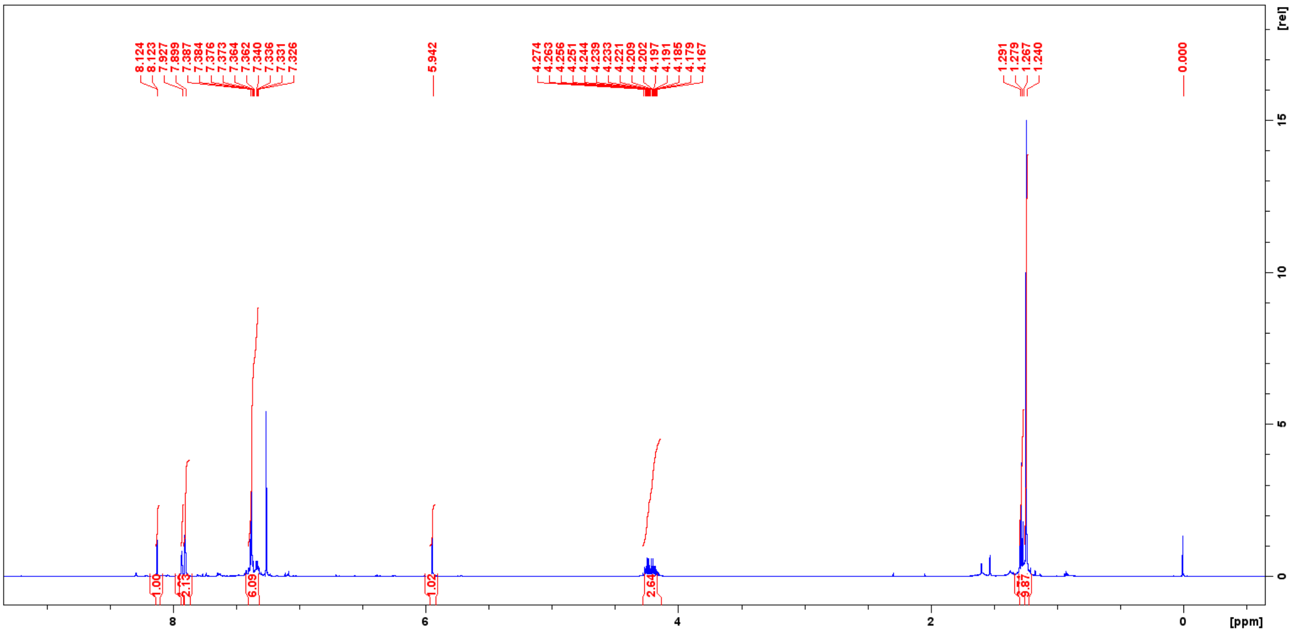

$^{13}$C NMR (150 MHz, CDCl3) spectrum of **7i**.

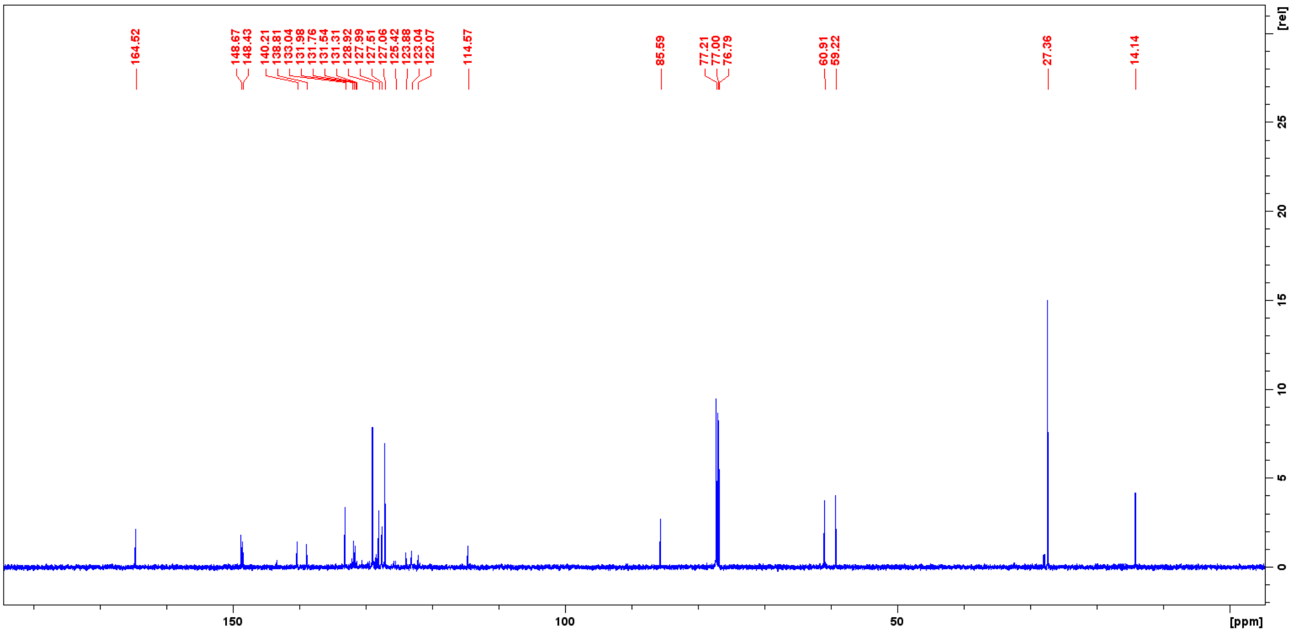

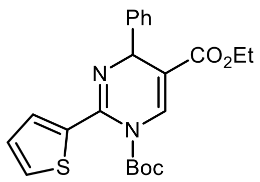

**7j**

$^{1}$H NMR (600 MHz, $CDCl_3$) spectrum of **7j**.

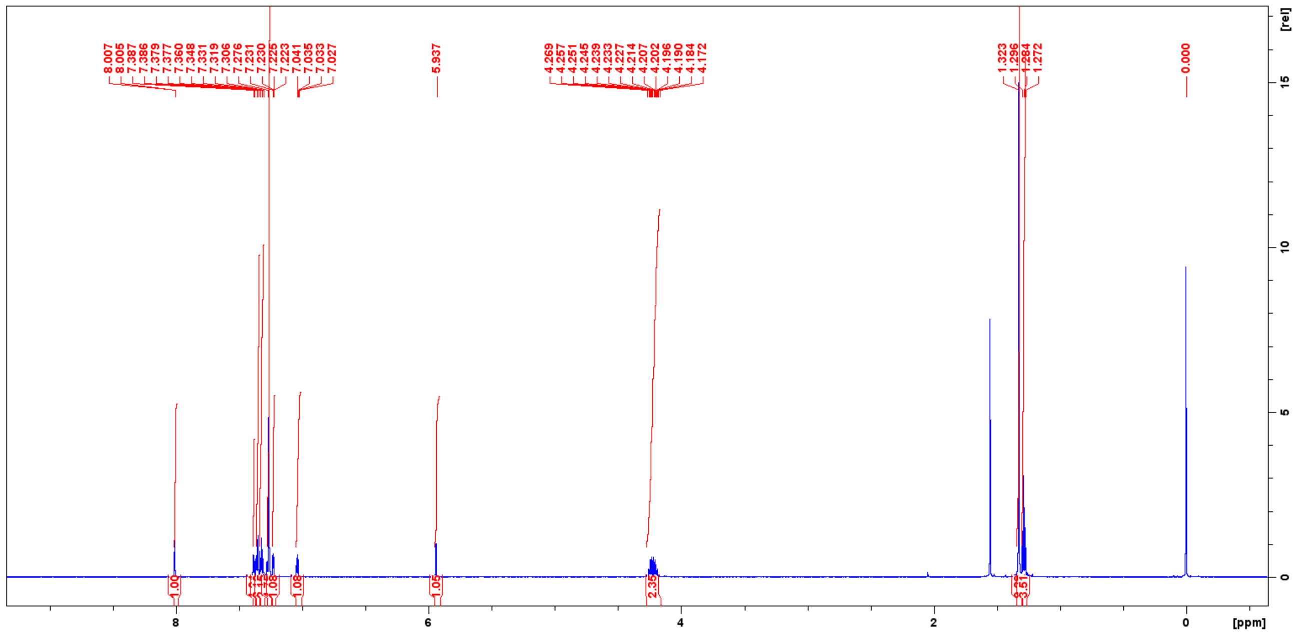

$^{13}$C NMR (150 MHz, $CDCl_3$) spectrum of **7j**.

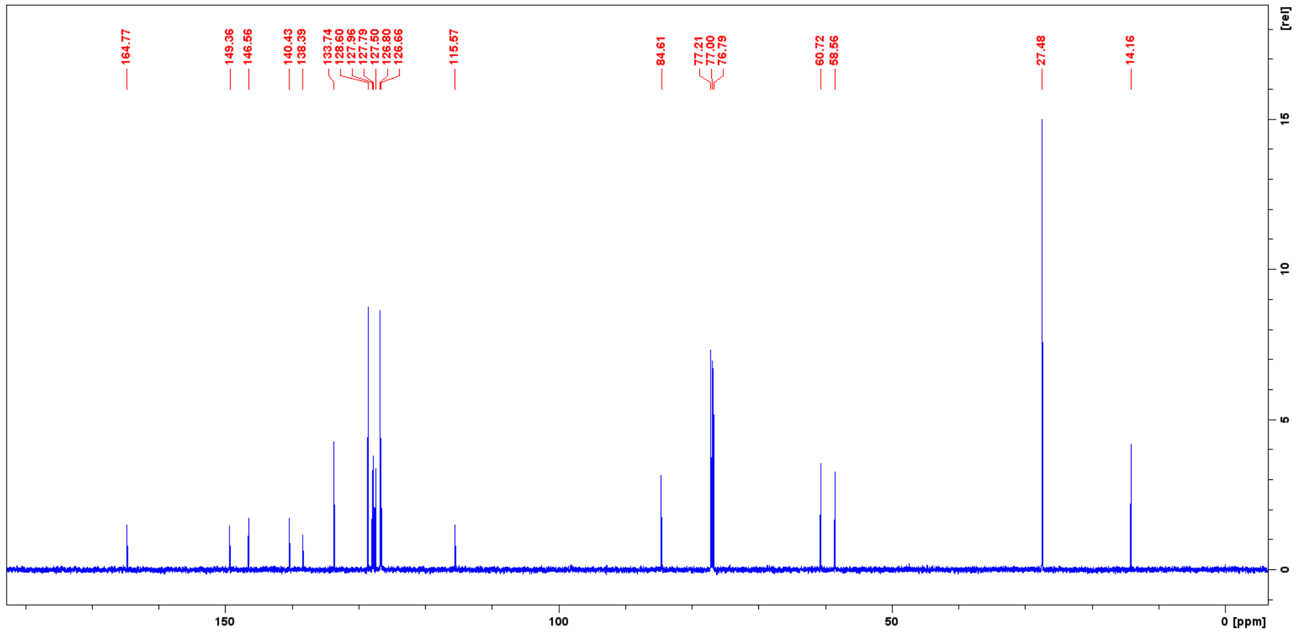

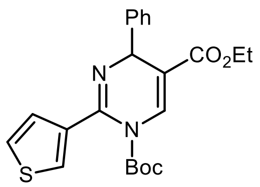


**7k**

$^{1}$H NMR (600 MHz, $CDCl_3$) spectrum of **7k**.

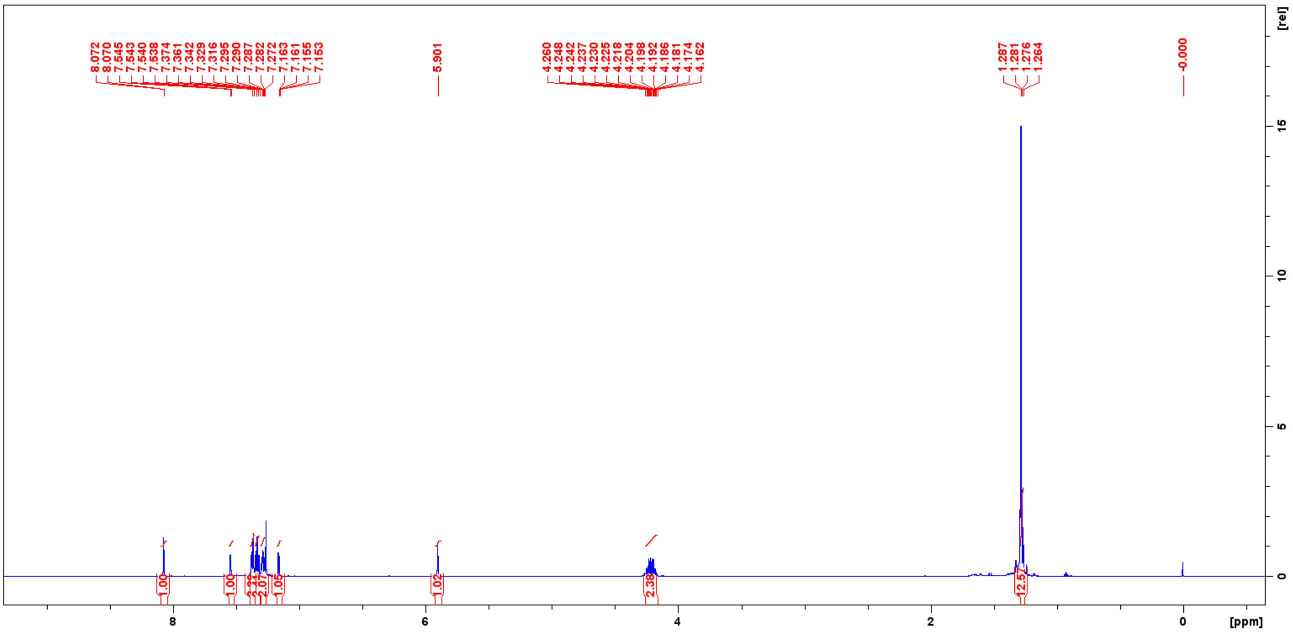

$^{13}$C NMR (150 MHz, $CDCl_3$) spectrum of **7k**.

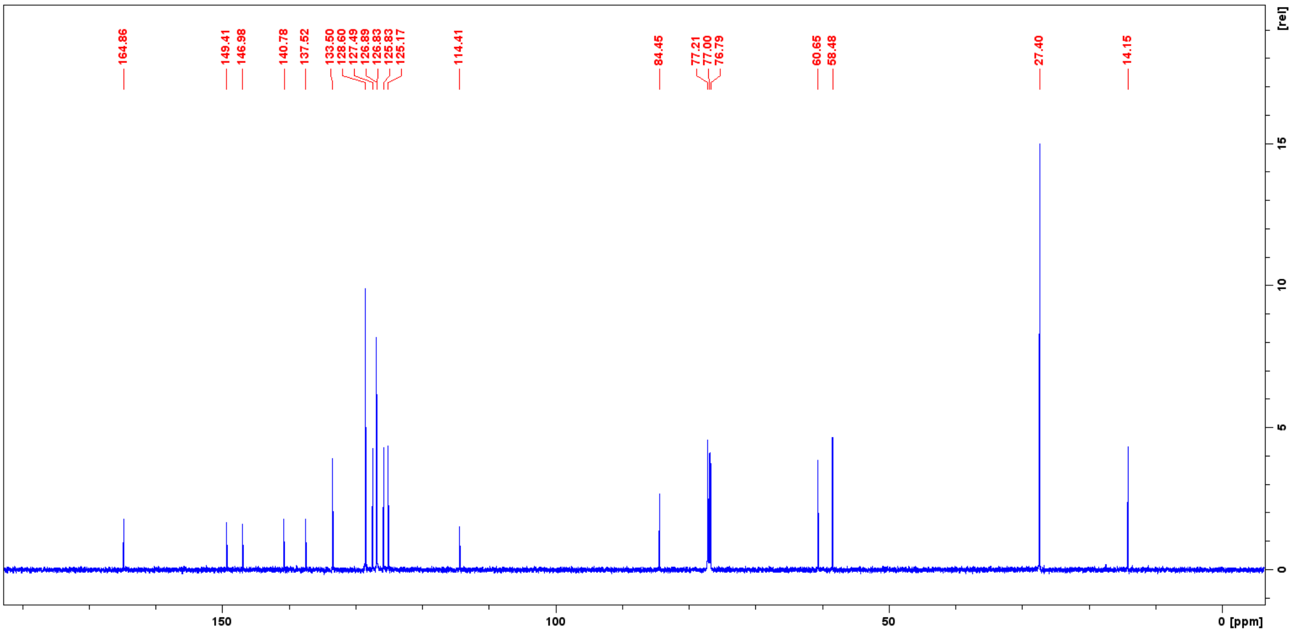

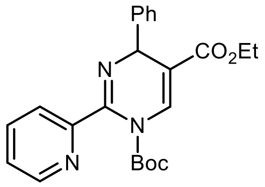

**7l**

$^{1}$H NMR (600 MHz, $CDCl_3$) spectrum of **7l**.

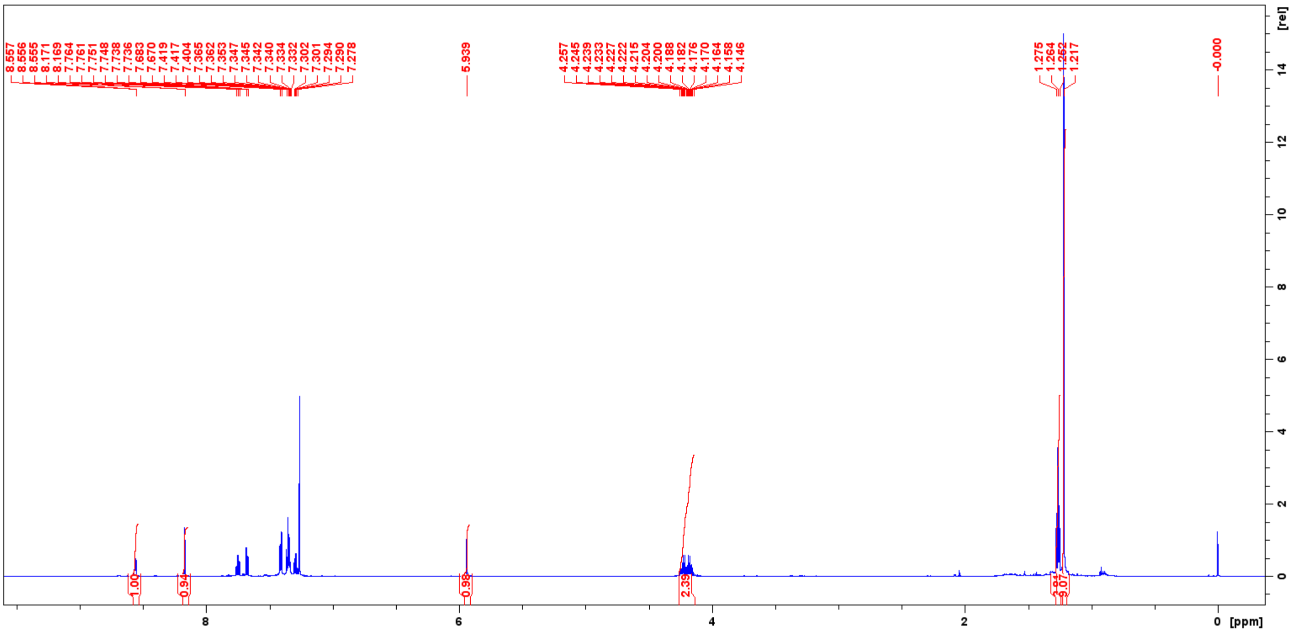

$^{13}$C NMR (150 MHz, $CDCl_3$) spectrum of **7l**.

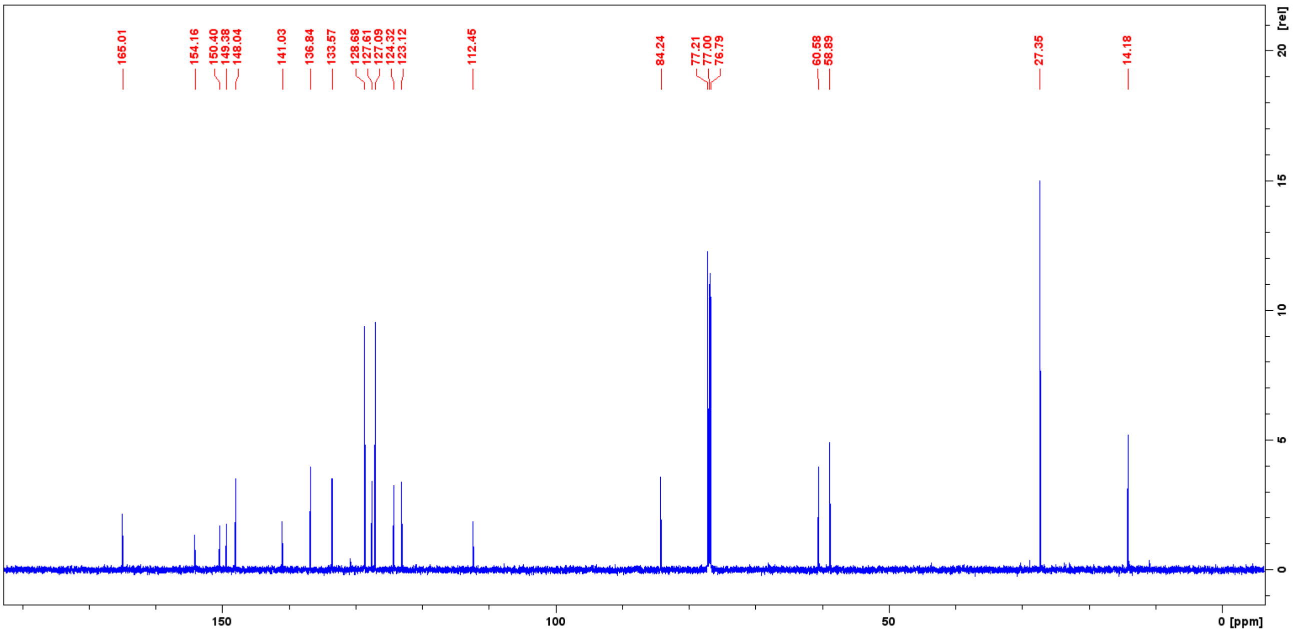

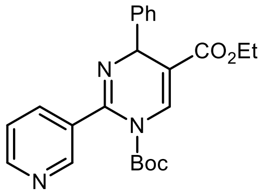


**7m**

$^{1}$H NMR (600 MHz, $CDCl_3$) spectrum of **7m**.

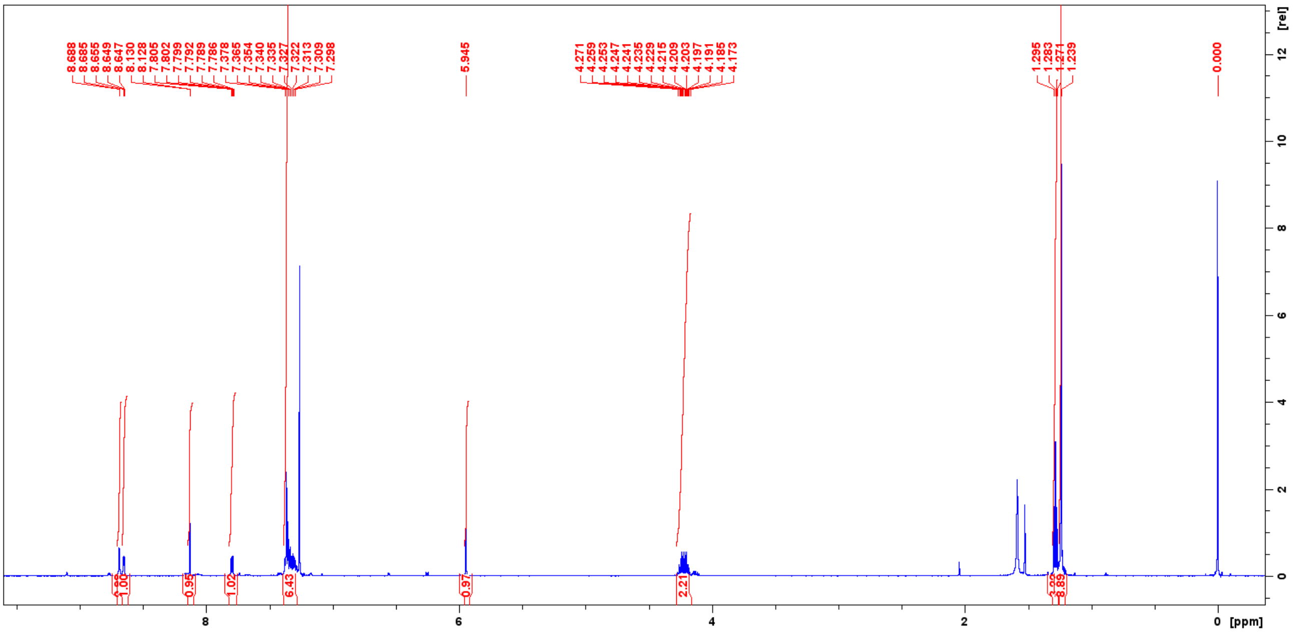


$^{13}$C NMR (150 MHz, $CDCl_3$) spectrum of **7m**.

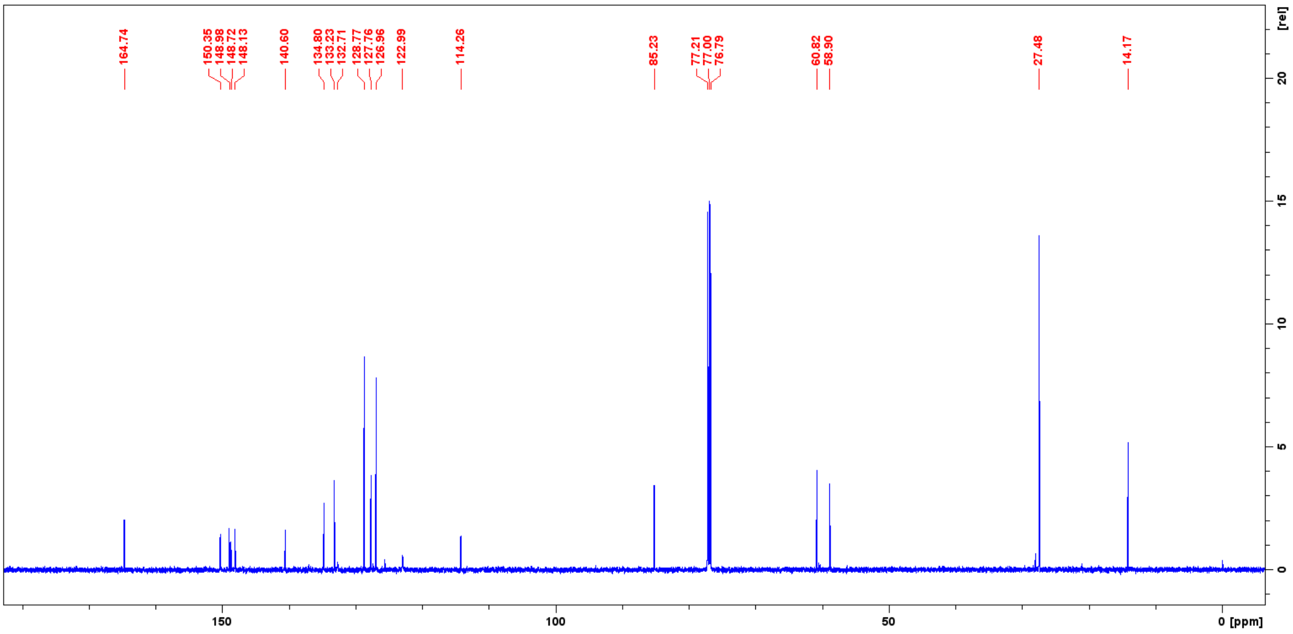

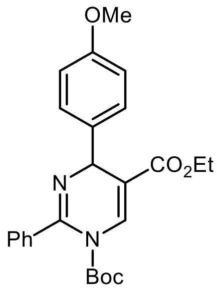


**7n**

$^{1}$H NMR (600 MHz, $CDCl_3$) spectrum of **7n**.

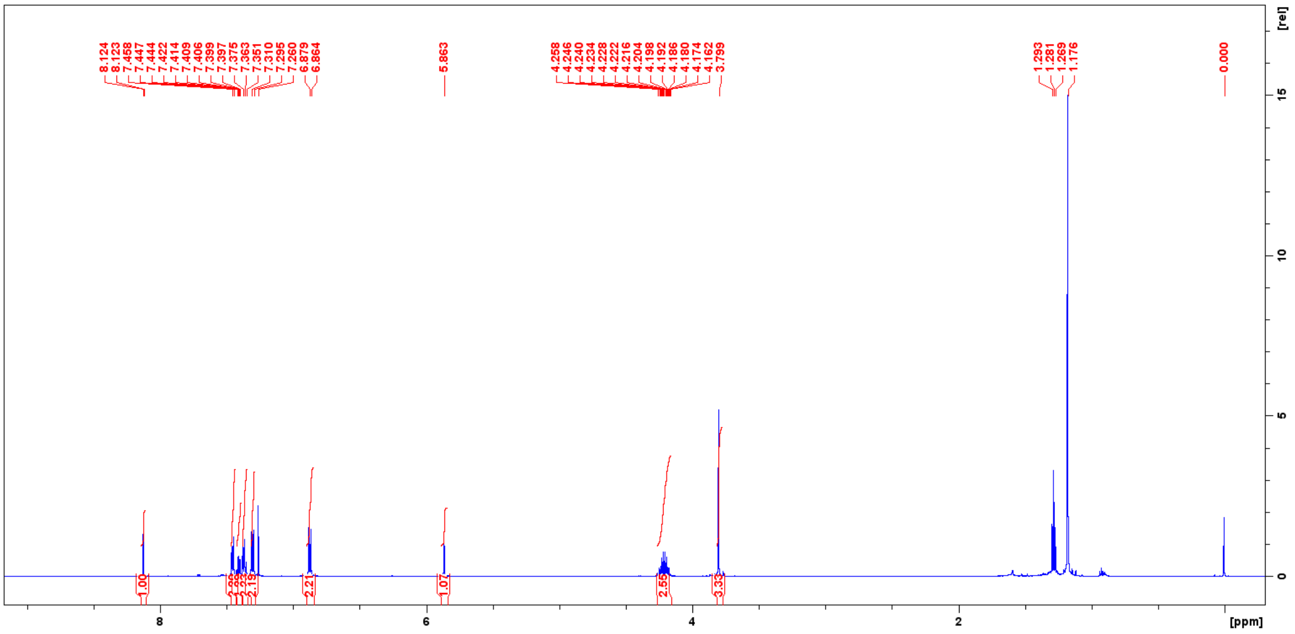


$^{13}$C NMR (150 MHz, $CDCl_3$) spectrum of **7n**.

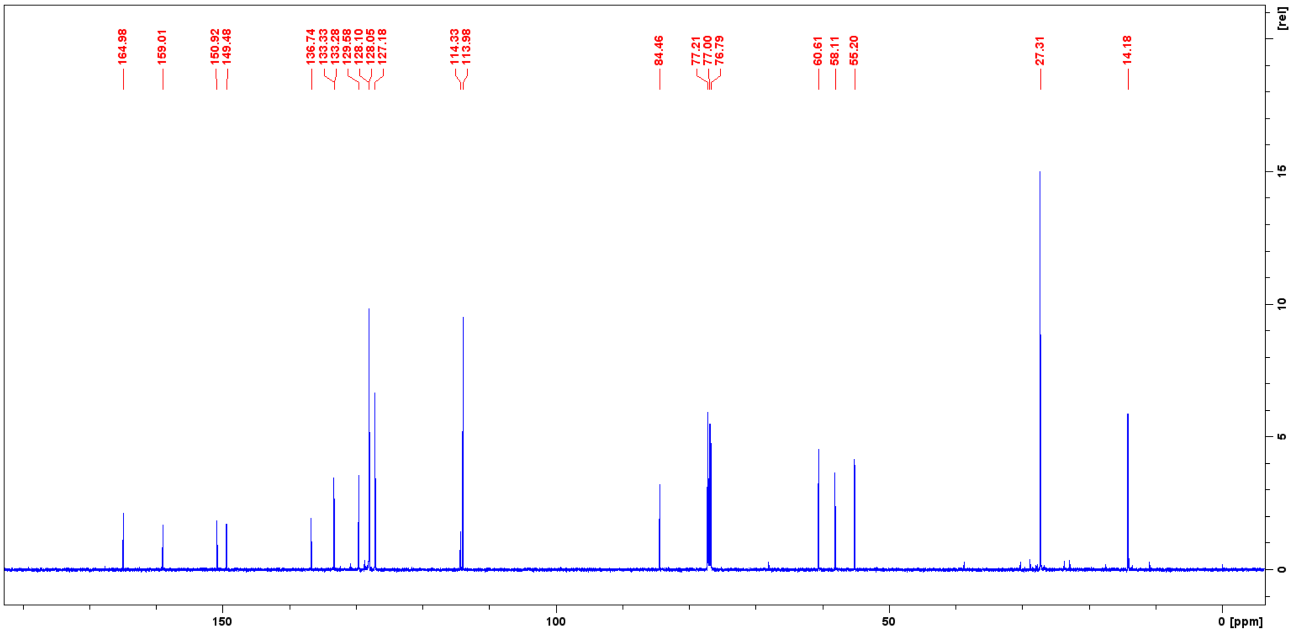

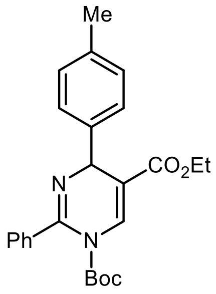


**7o**

$^{1}$H NMR (600 MHz, $CDCl_3$) spectrum of **7o**.

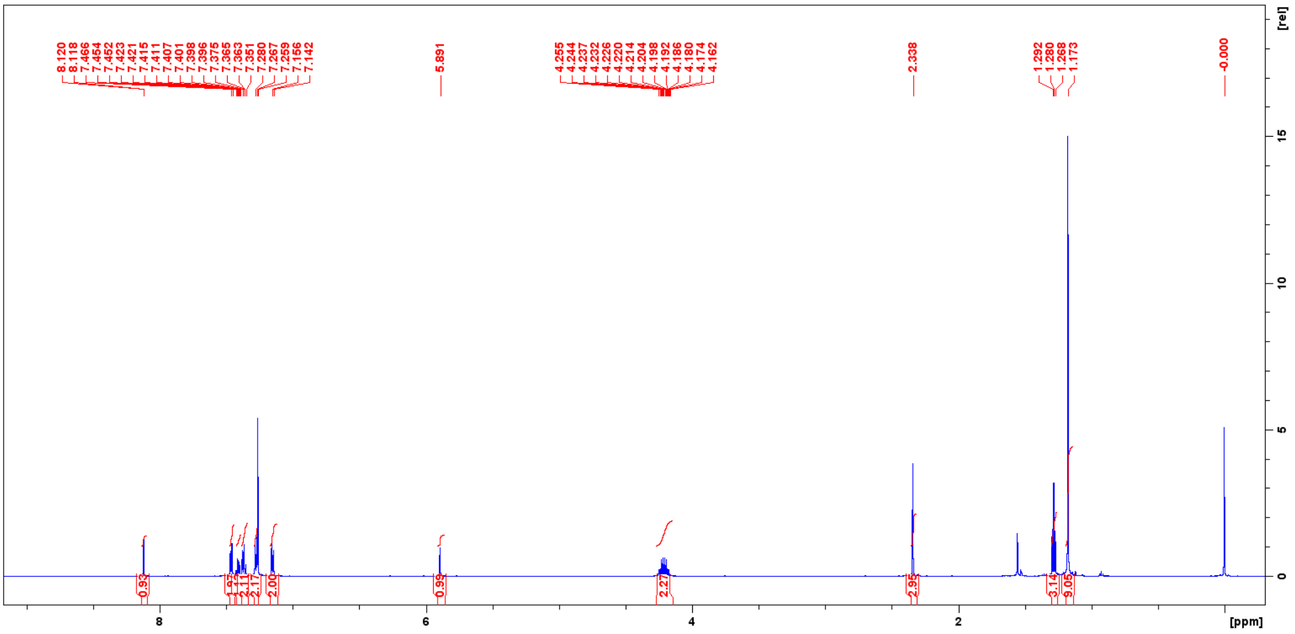


$^{13}$C NMR (150 MHz, $CDCl_3$) spectrum of **7o**.

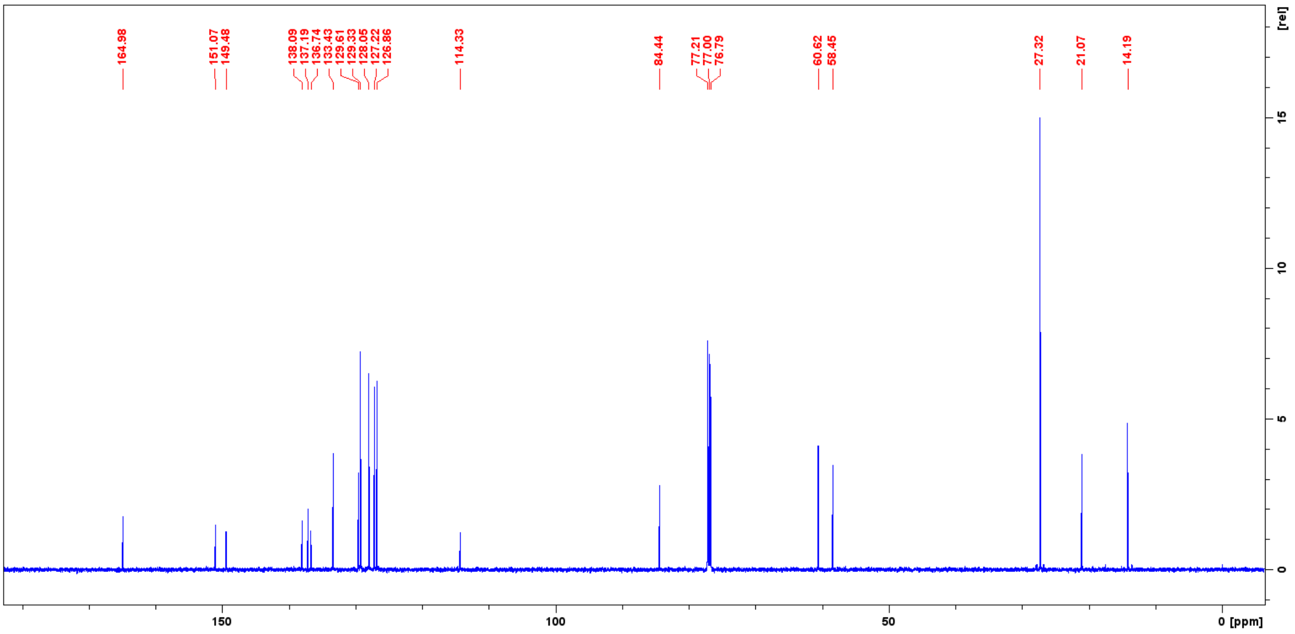

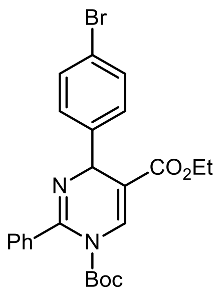


**7p**

$^{1}$H NMR (600 MHz, $CDCl_3$) spectrum of **7p**.

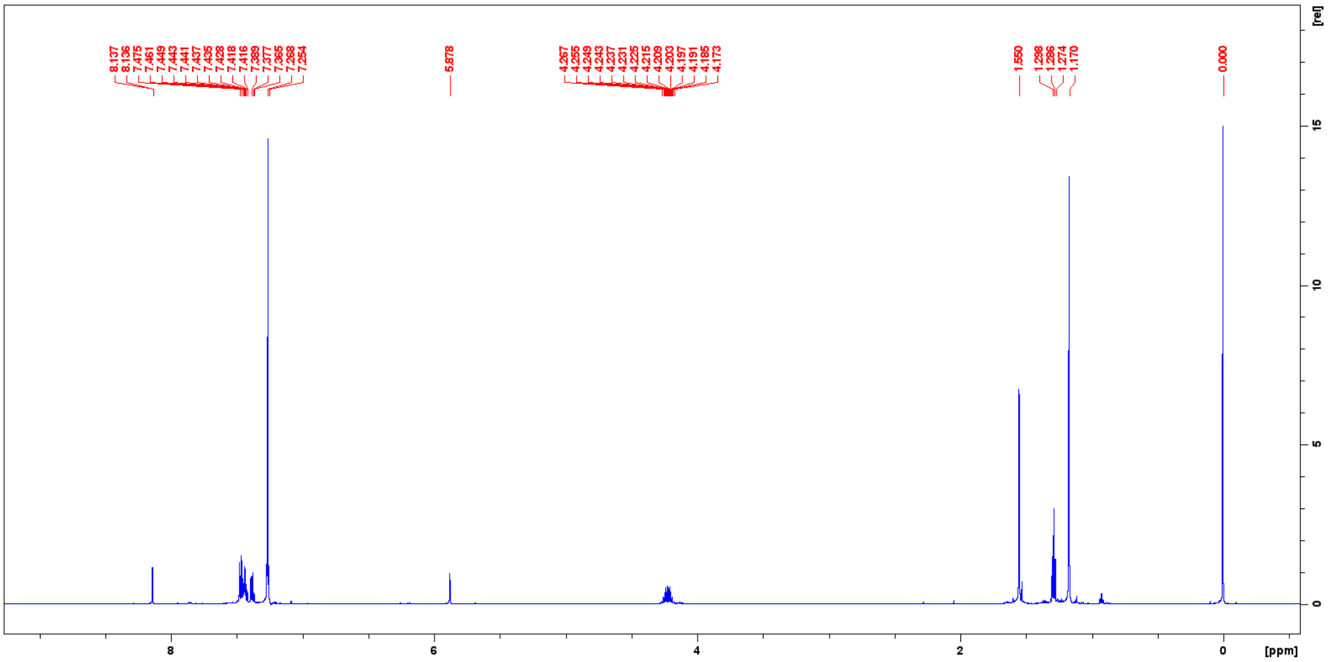

$^{13}$C NMR (150 MHz, $CDCl_3$) spectrum of **7p**.

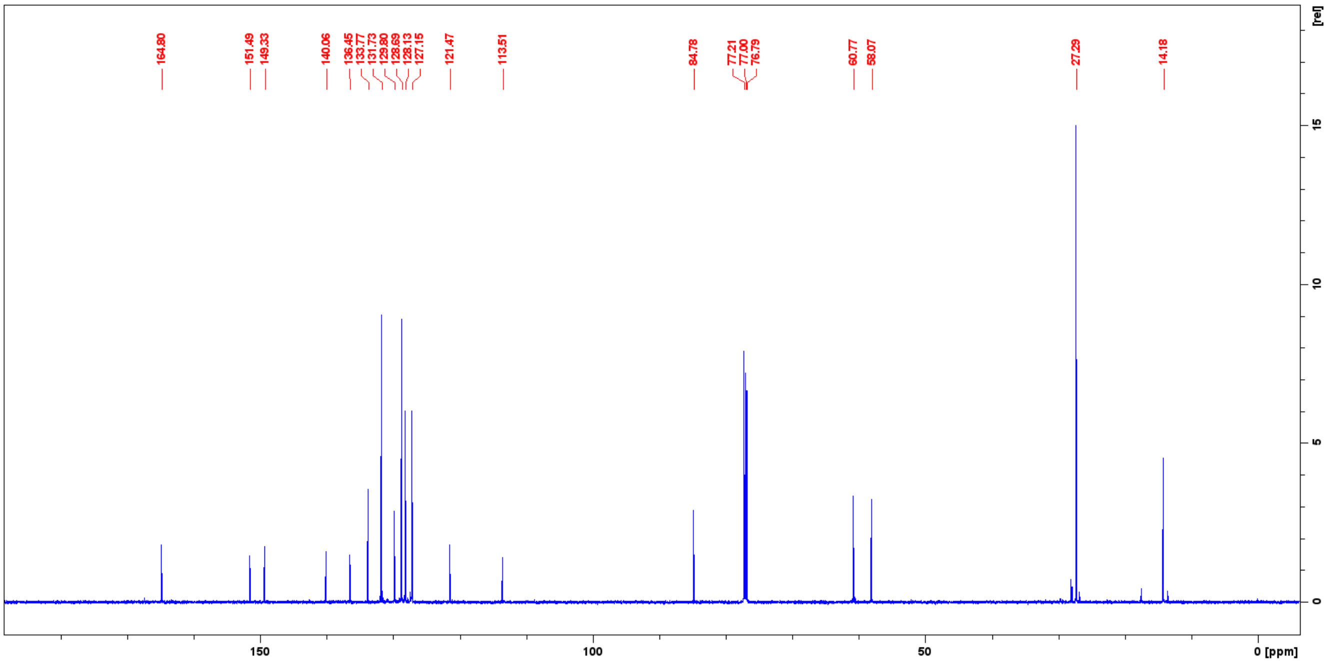

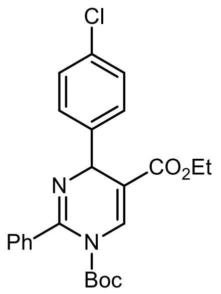


**7q**

$^{1}$H NMR (600 MHz, $CDCl_3$) spectrum of **7q**.

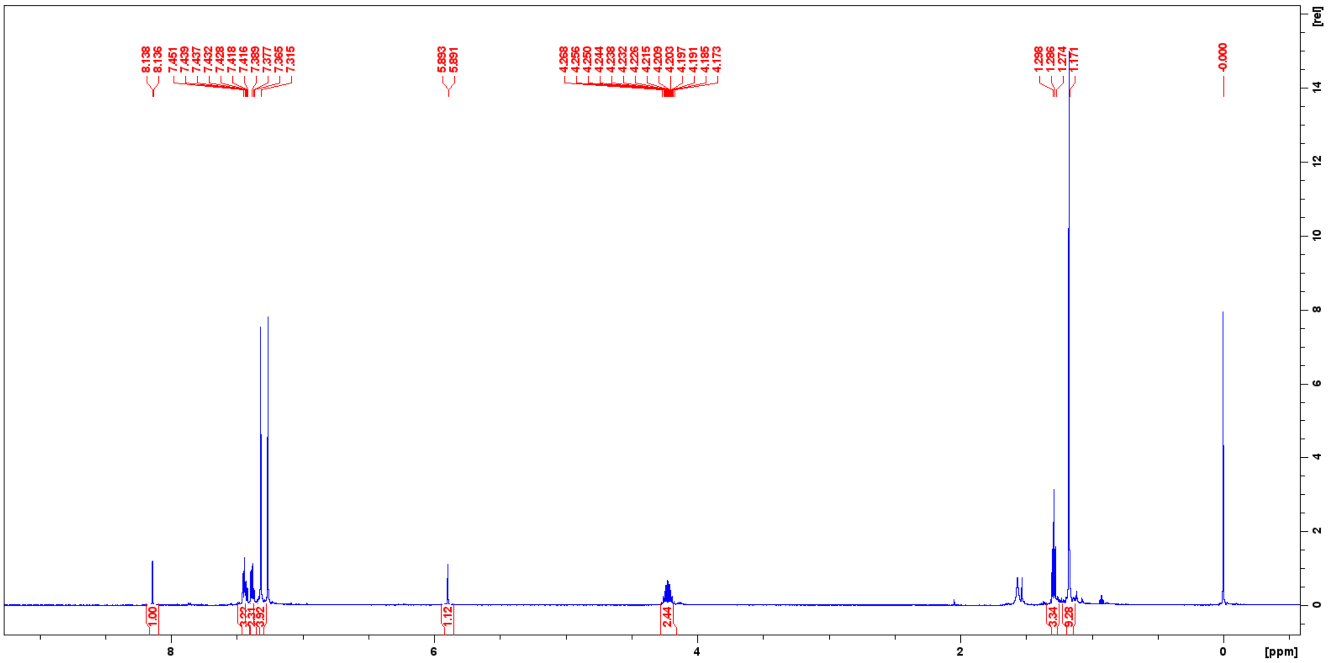


$^{13}$C NMR (150 MHz, $CDCl_3$) spectrum of **7q**.

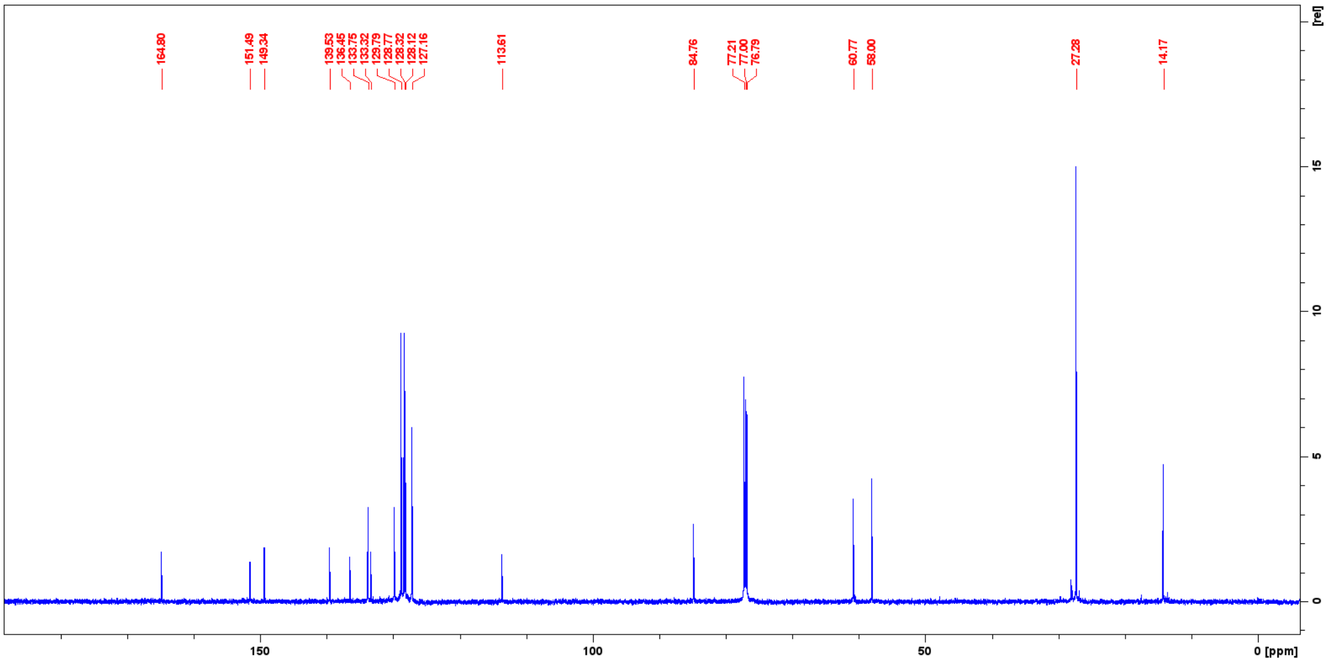

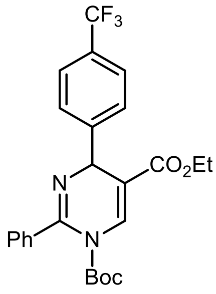


**7r**

$^{1}$H NMR (600 MHz, $CDCl_3$) spectrum of **7r**.

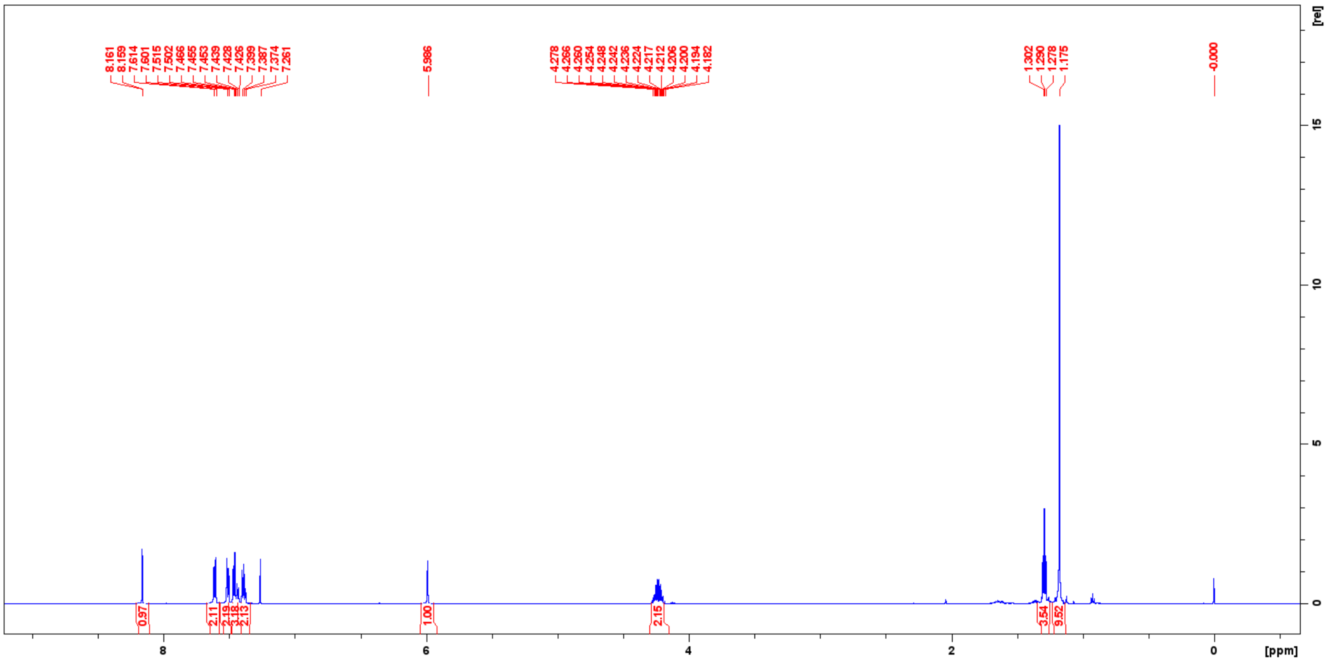


$^{13}$C NMR (150 MHz, $CDCl_3$) spectrum of **7r**.

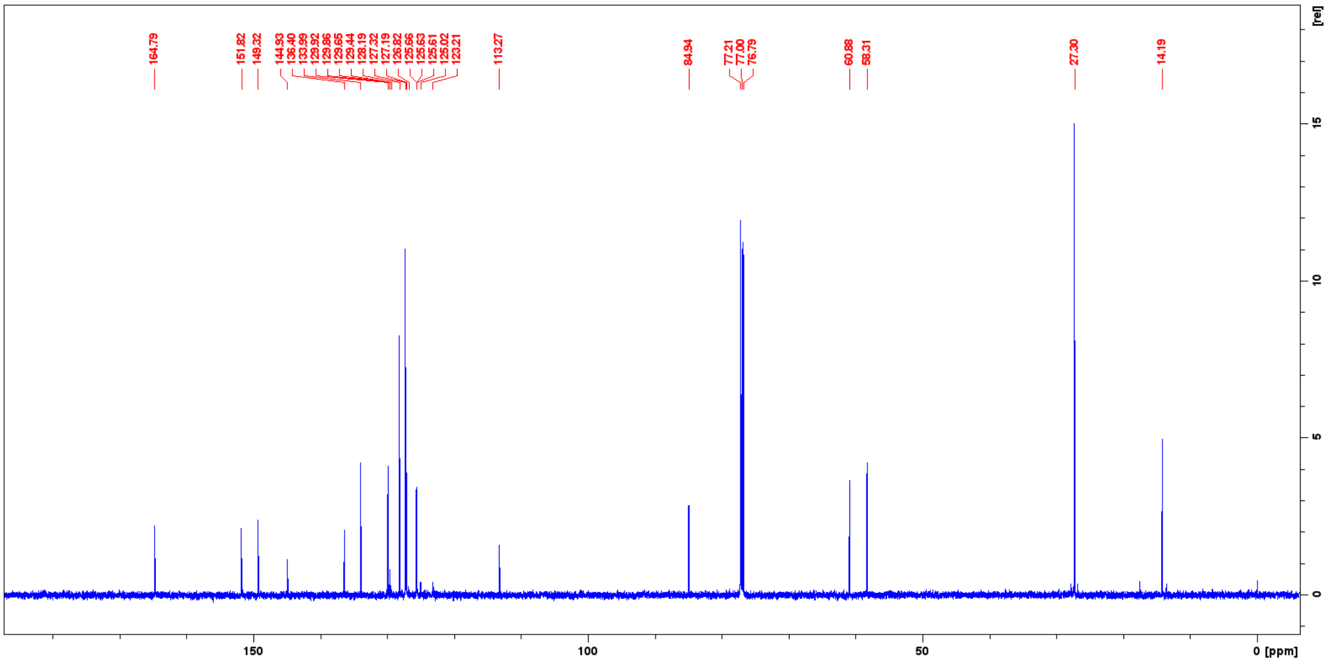

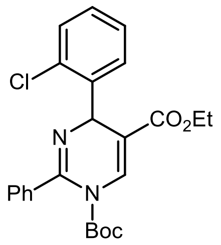


**7s**

$^{1}$H NMR (600 MHz, $CDCl_3$) spectrum of **7s**.

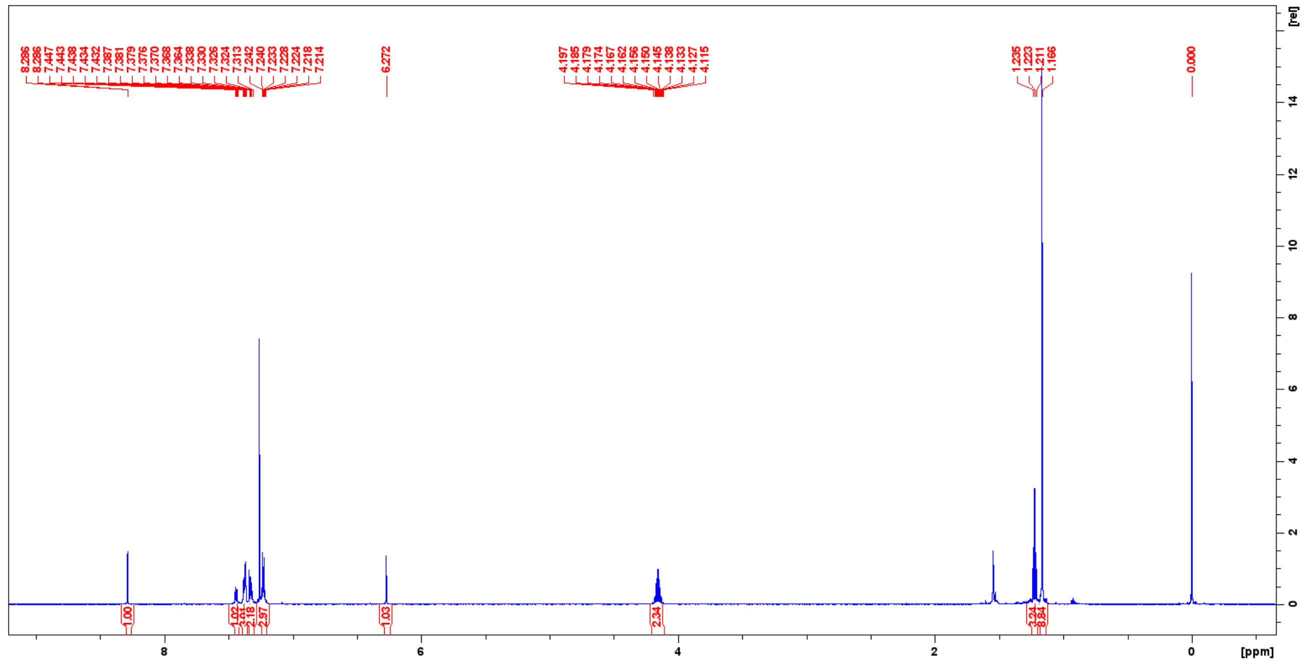

$^{13}$C NMR (150 MHz, $CDCl_3$) spectrum of **7s**.

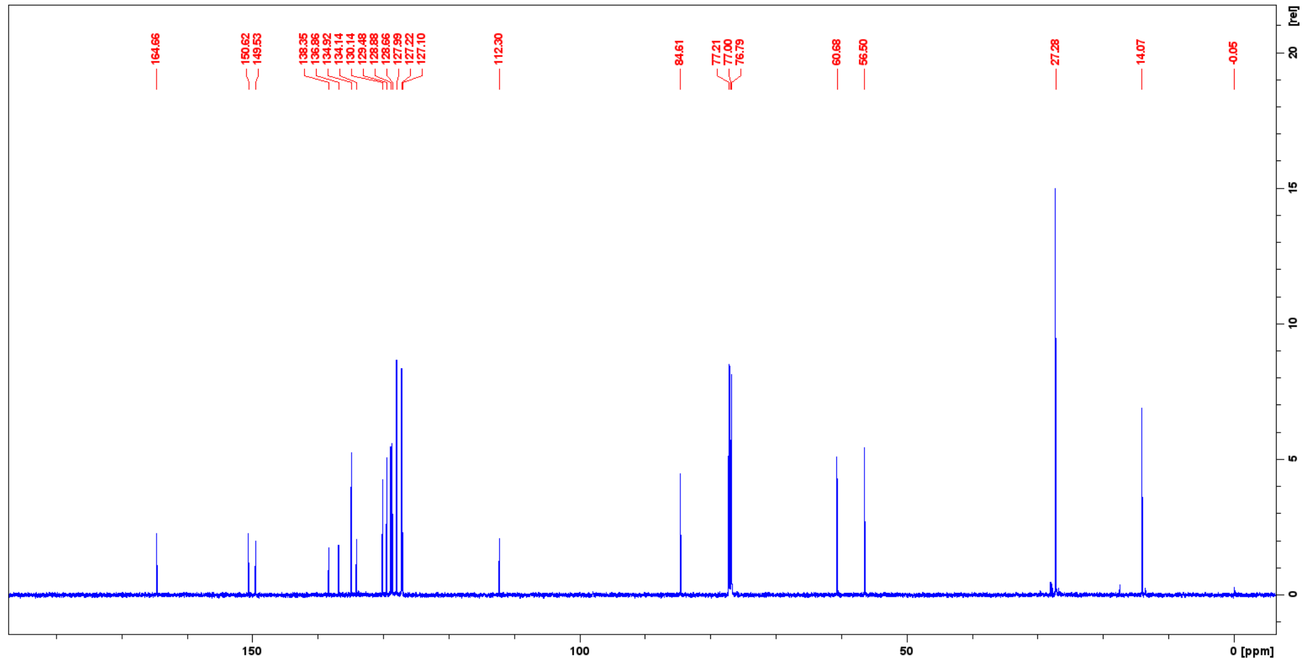

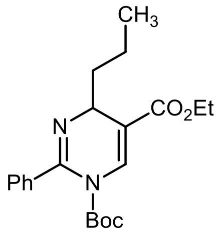


**7t**

$^{1}$H NMR (600 MHz, $CDCl_3$) spectrum of **7t**.

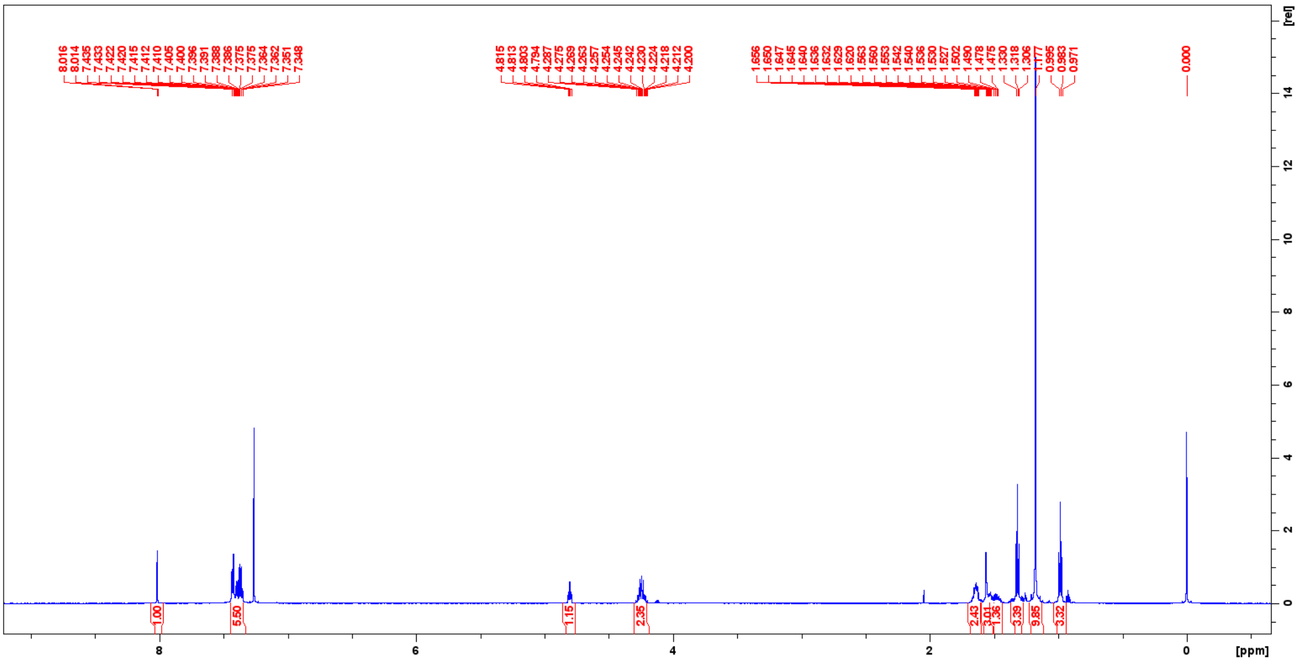


$^{13}$C NMR (150 MHz, $CDCl_3$) spectrum of **7t**.

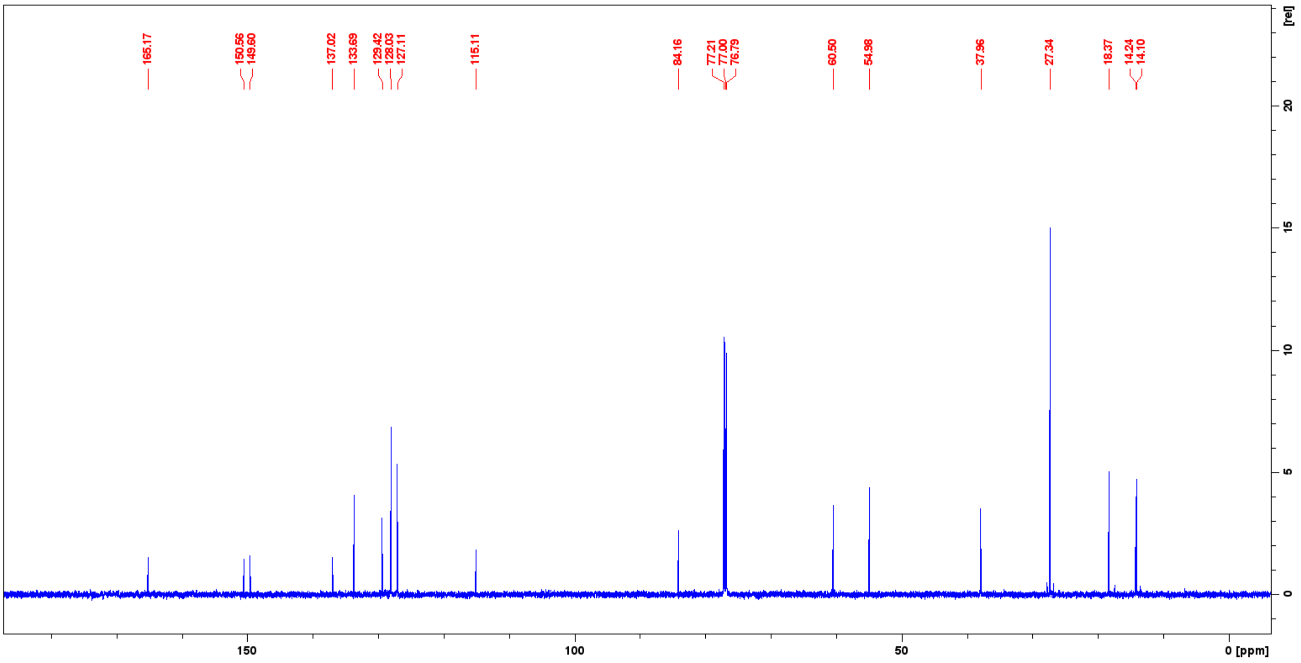

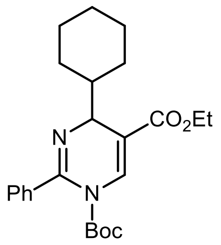


**7u**

$^{1}$H NMR (600 MHz, $CDCl_3$) spectrum of **7u**.

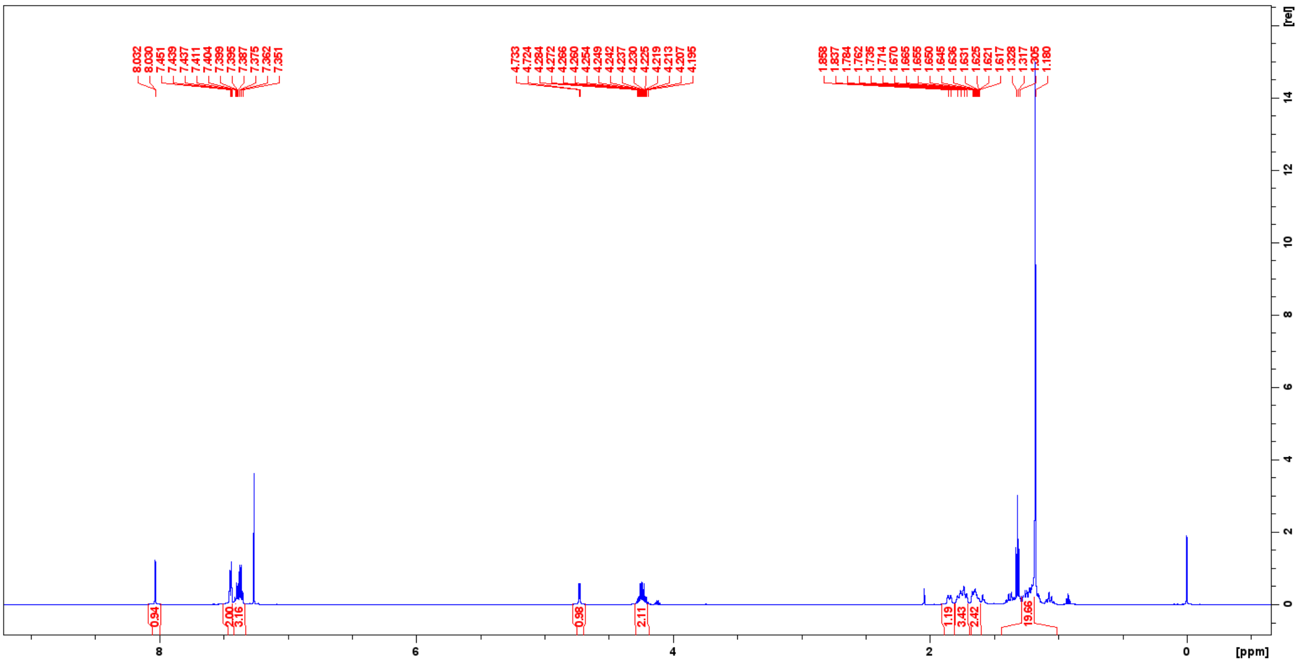


$^{13}$C NMR (150 MHz, $CDCl_3$) spectrum of **7u**.

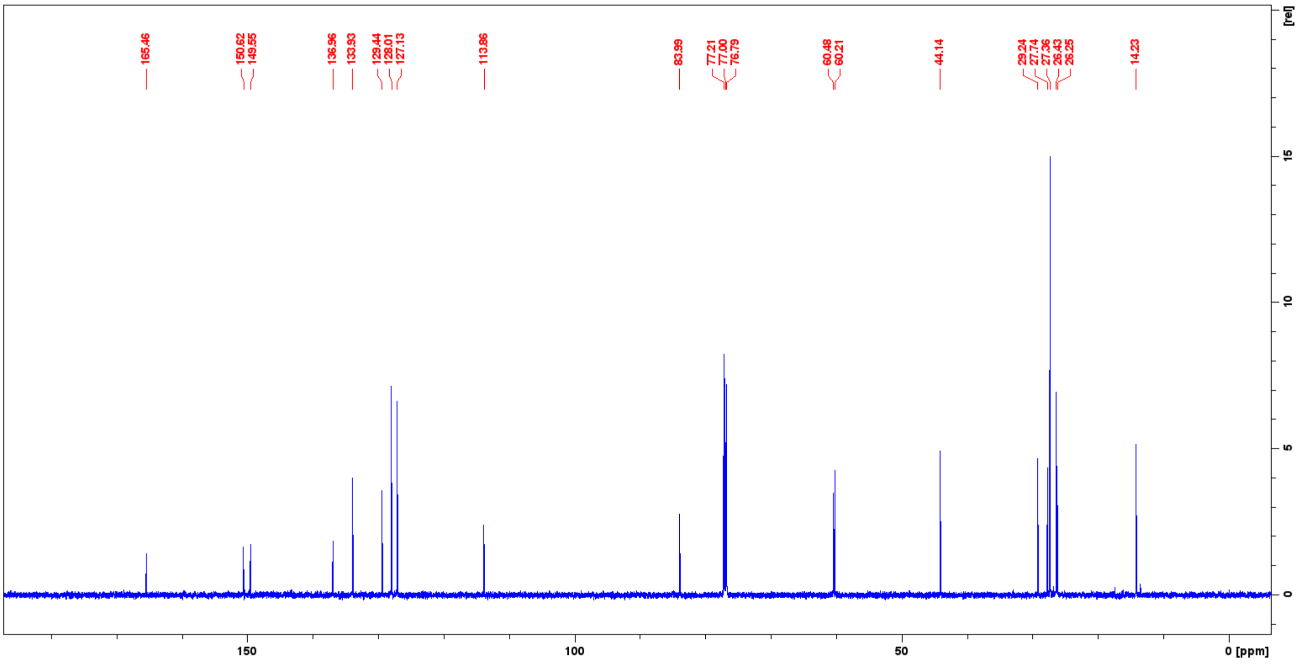

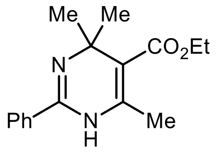

**9**

$^{1}$H NMR (500 MHz, $CD_3OD$) spectrum of **9**.

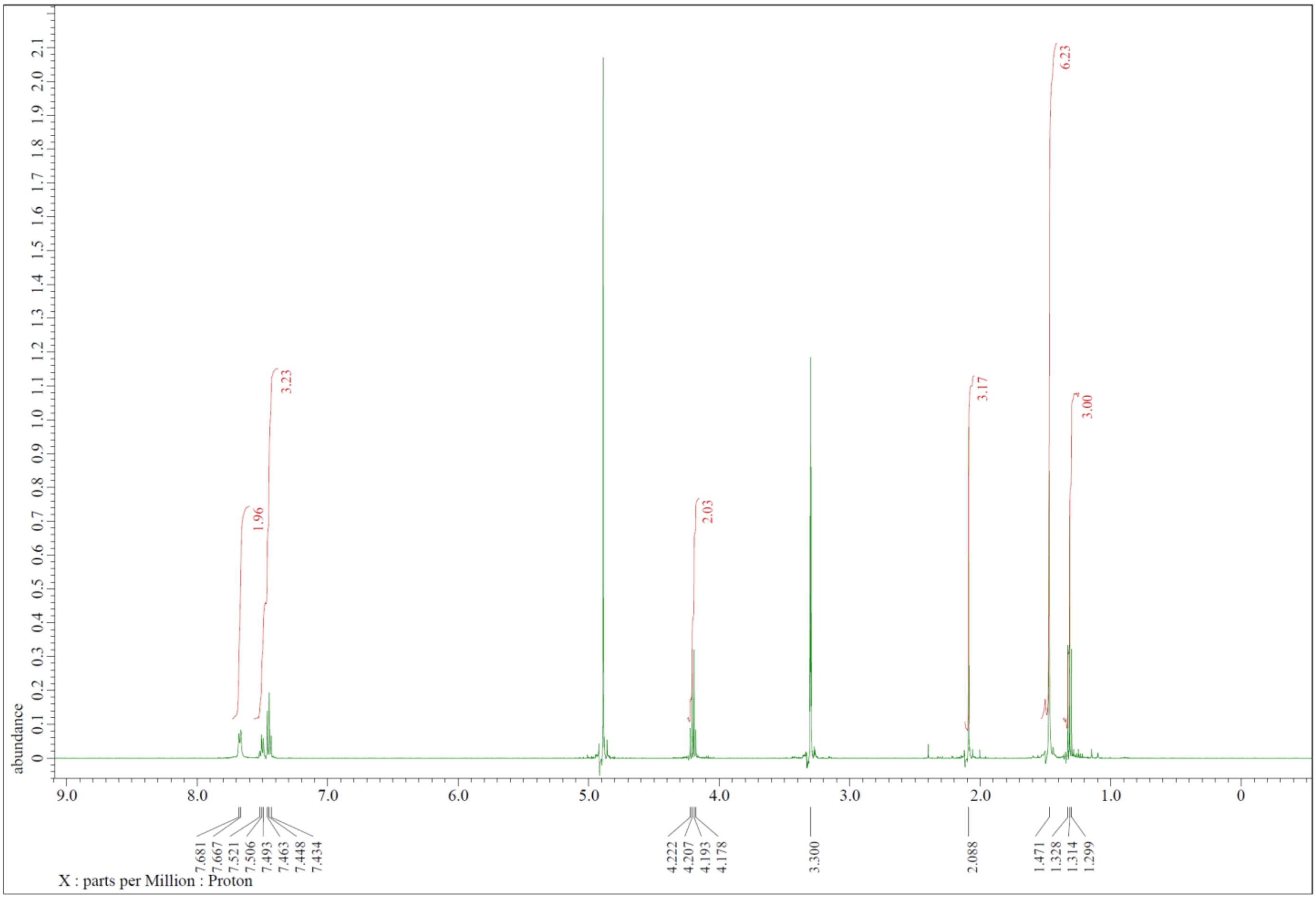

$^{13}C$ NMR (125 MHz, $CD_3OD$) spectrum of **9**.

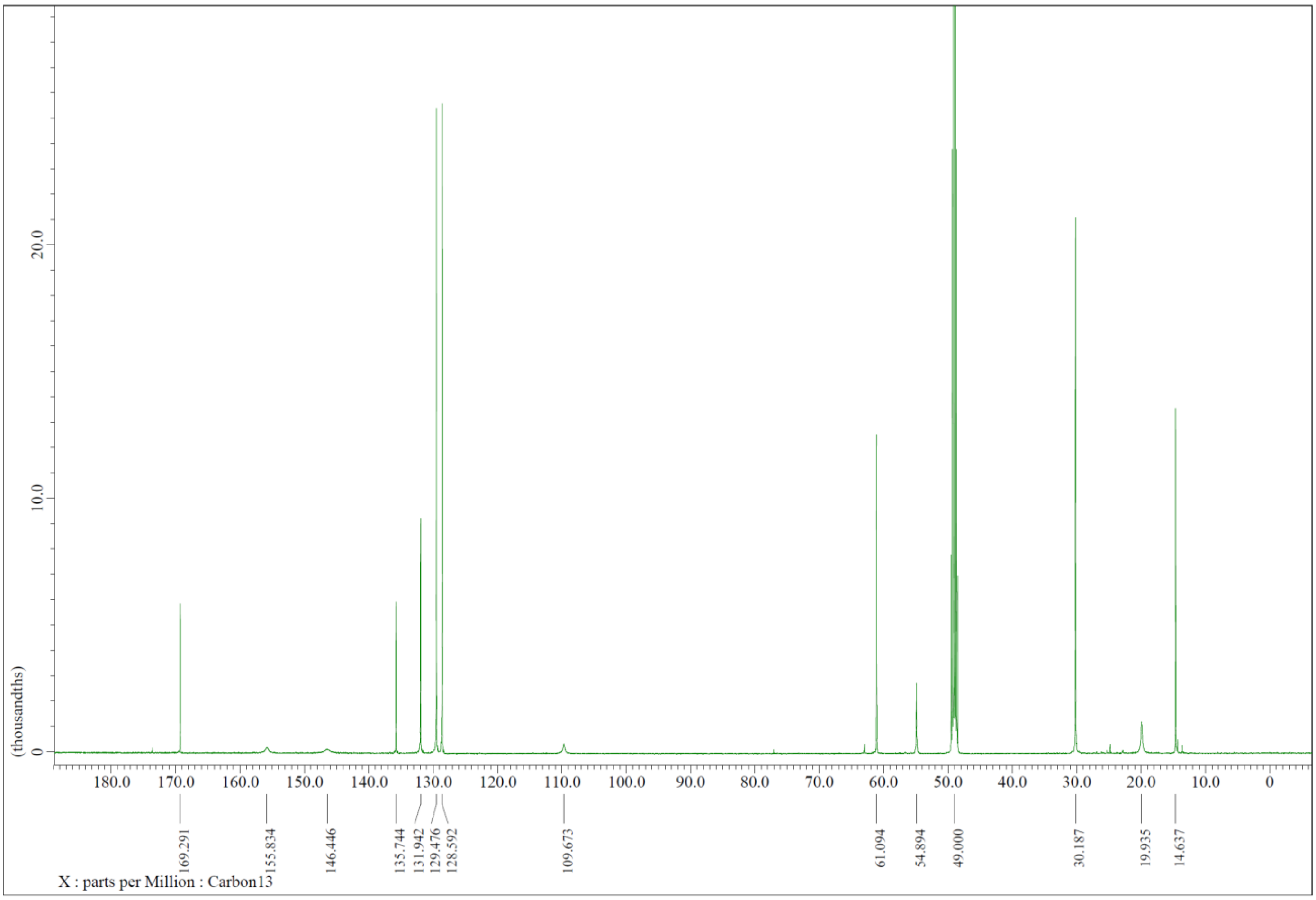

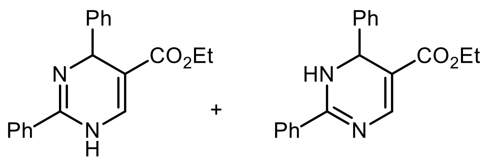


**10a** **11a**

$^1$H NMR (500 MHz, DMSO-$d_6$) spectrum of **10a** + **11a**.

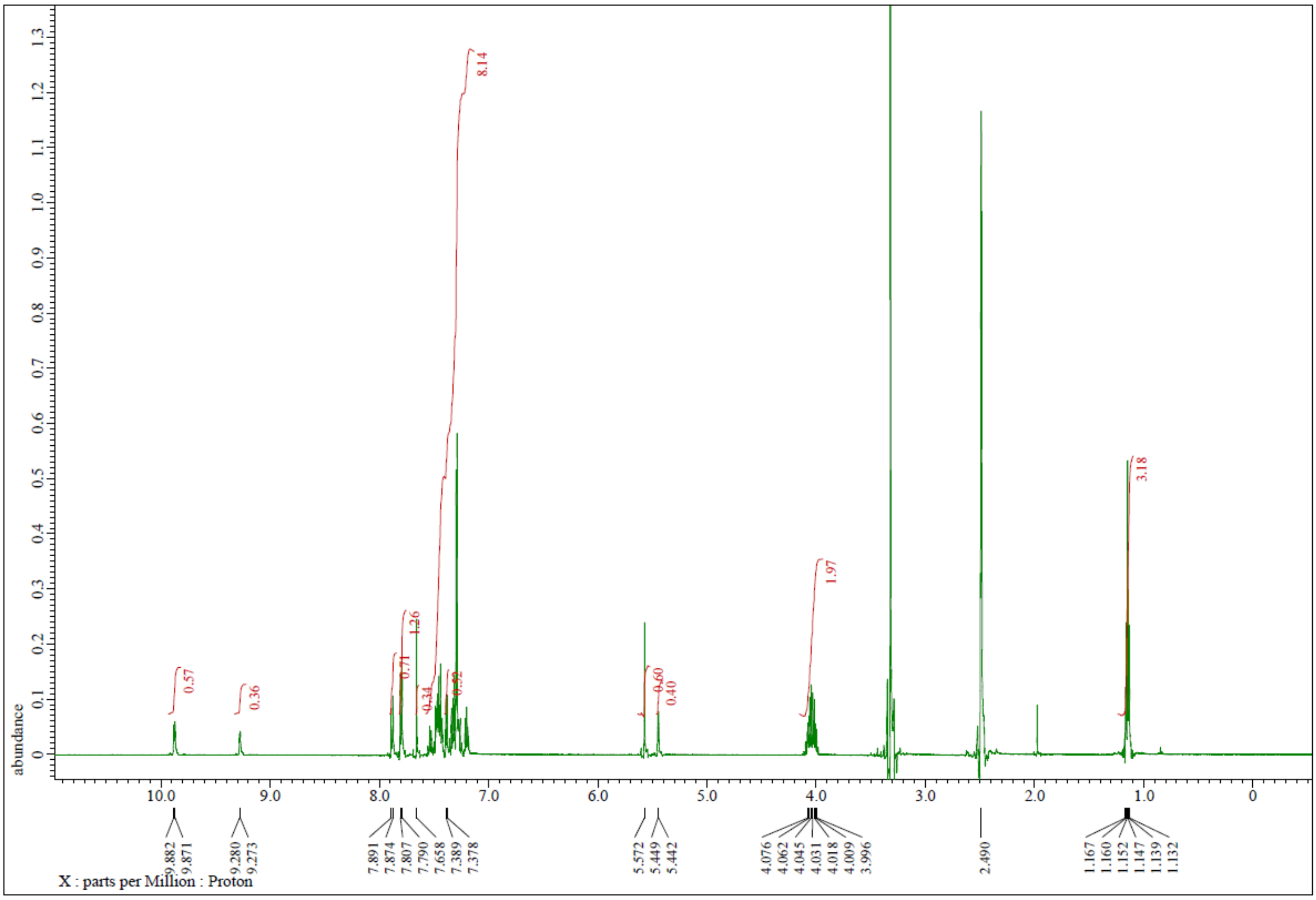

$^{1}$H NMR (500 MHz, $CD_3OD$) spectrum of **10a** + **11a**.

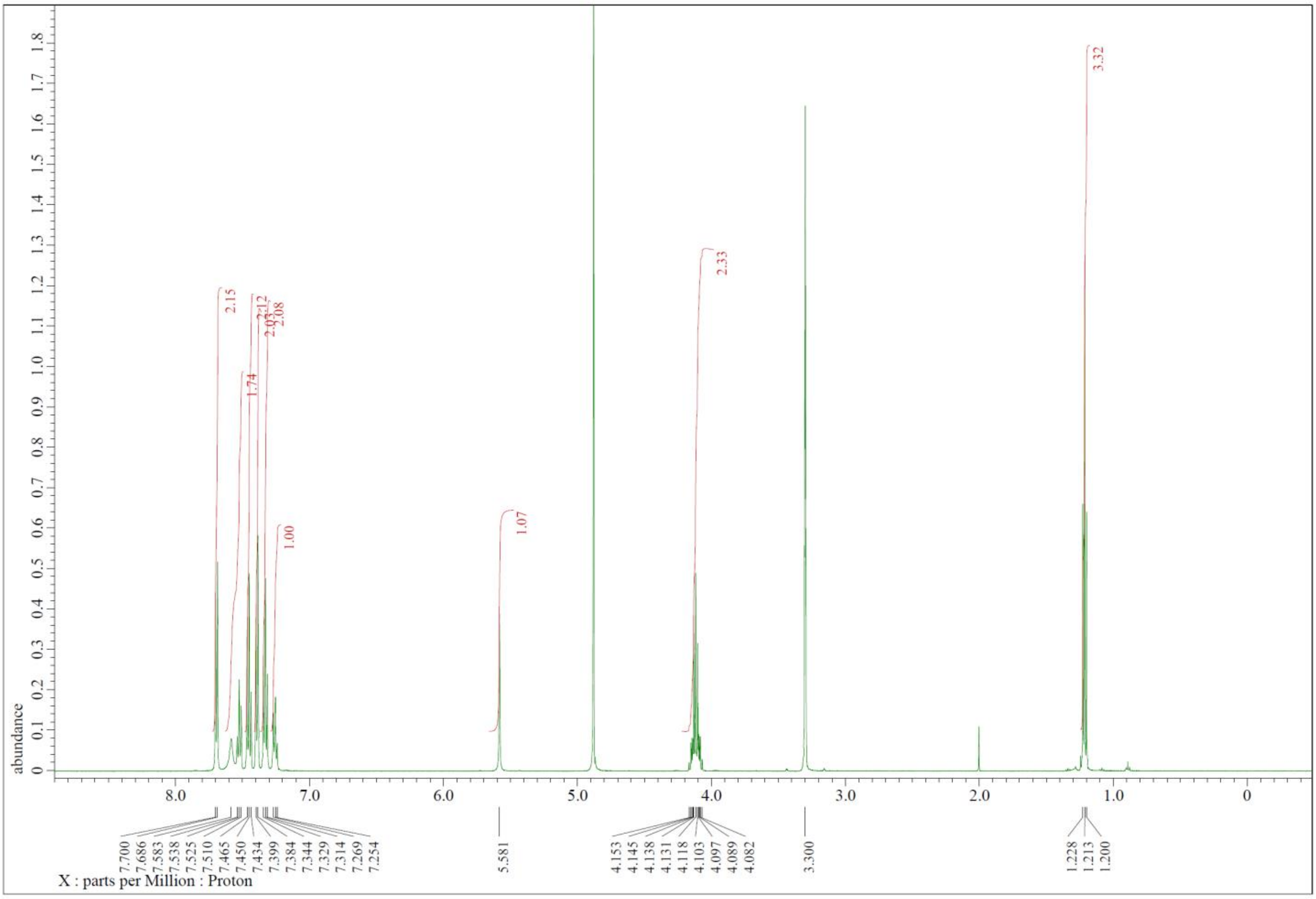

$^{13}C$ NMR (125 MHz, $CD_3OD$) spectrum of **10a** + **11a**.

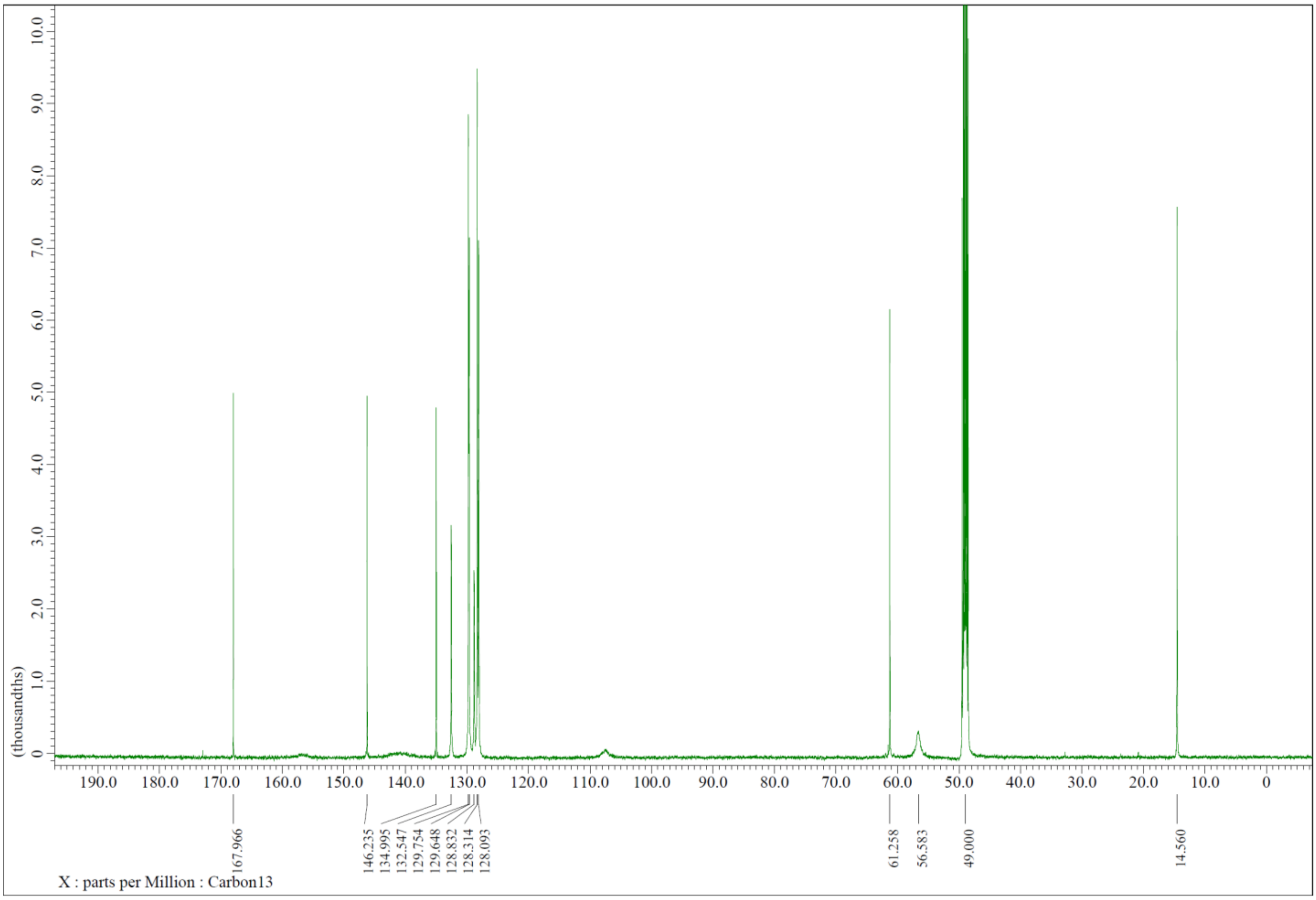

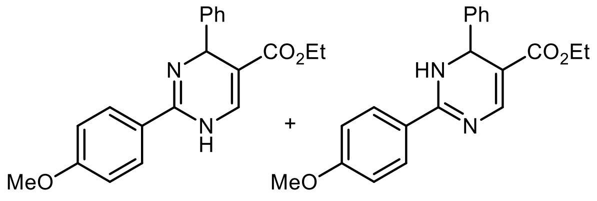


**10b** **11b**

$^{1}$H NMR (500 MHz, DMSO-$d_6$) spectrum of **10b** + **11b**.

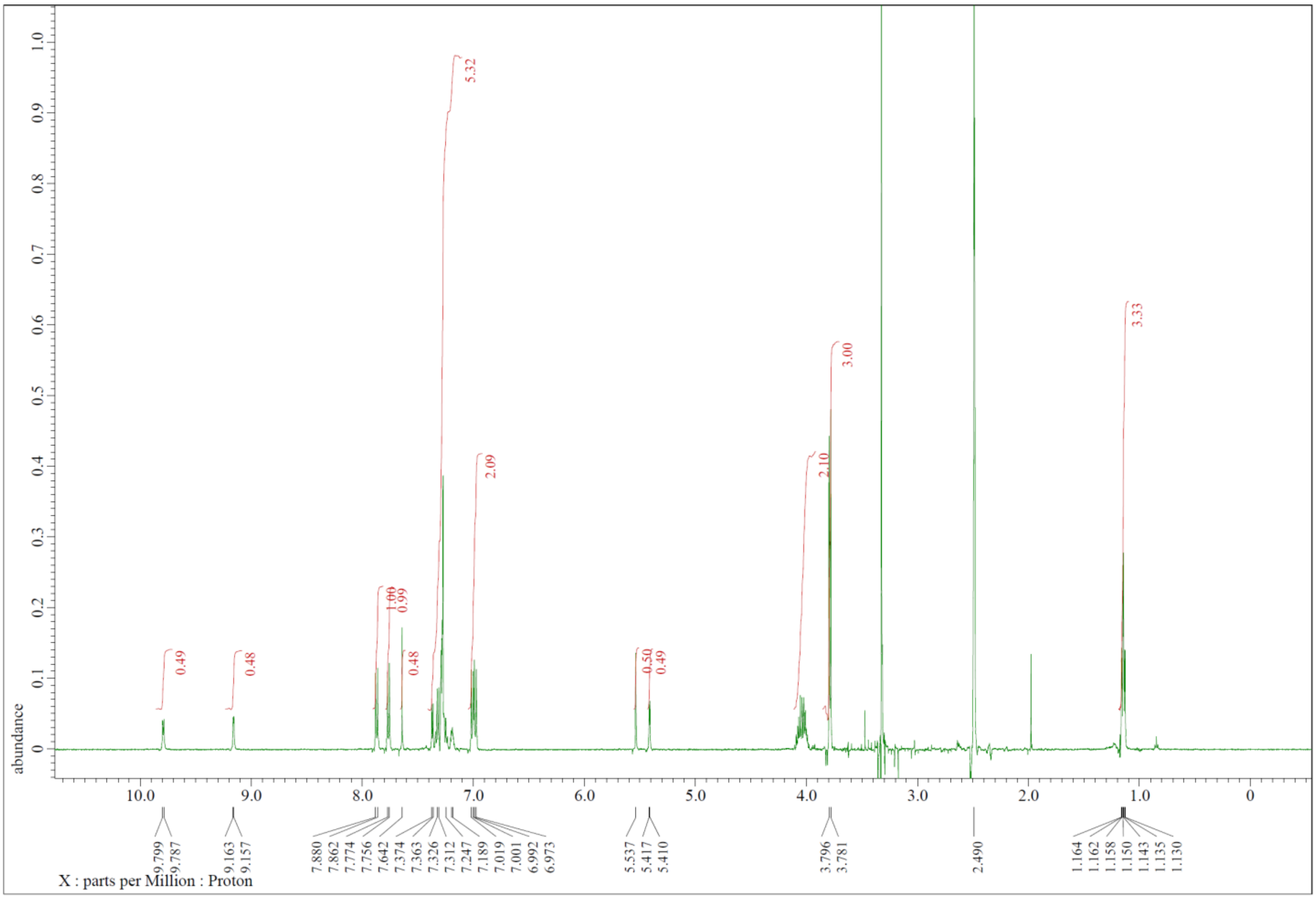

$^{1}$H NMR (500 MHz, $CD_3OD$) spectrum of **10b** + **11b**.

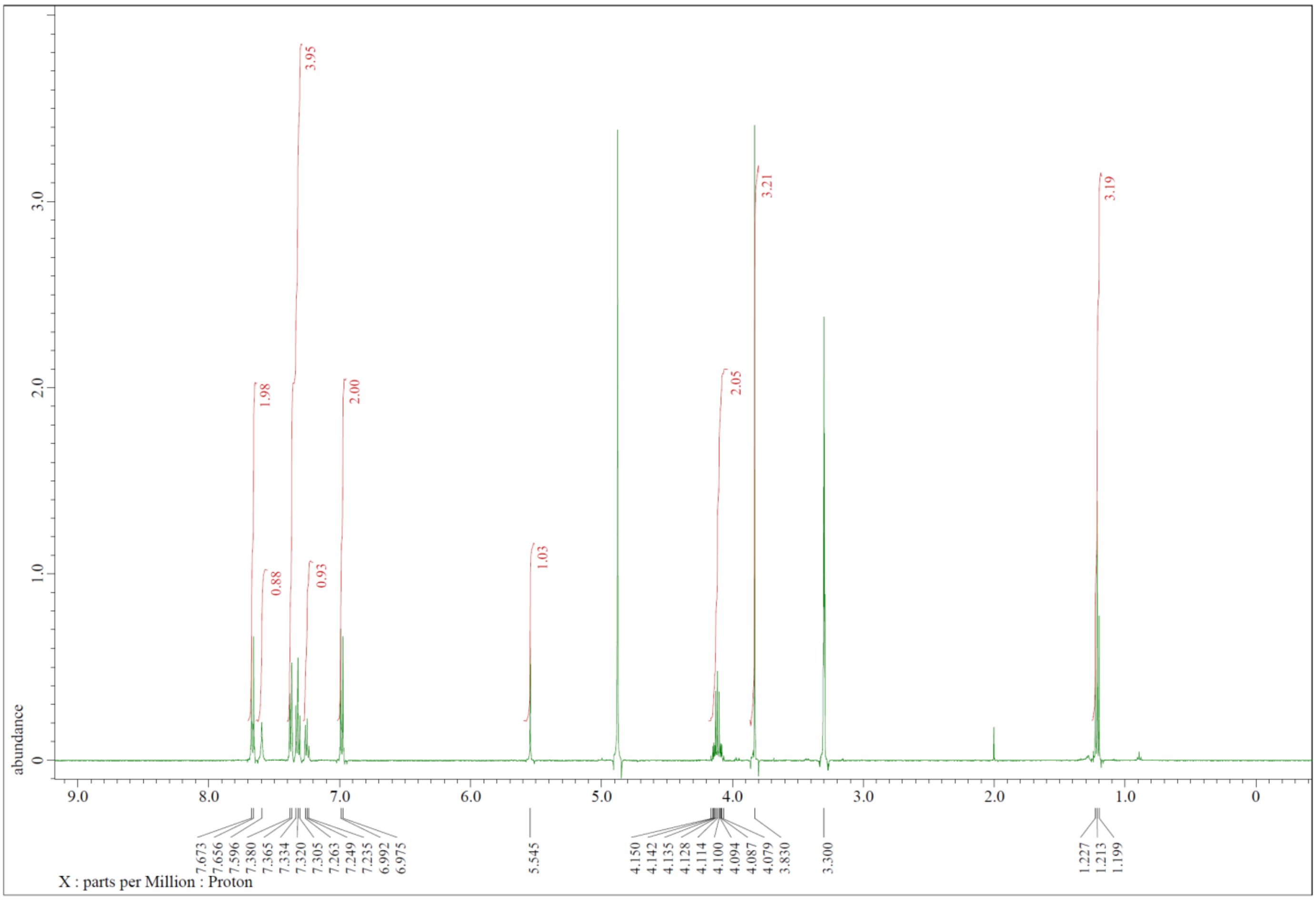

$^{13}C$ NMR (125 MHz, $CD_3OD$) spectrum of **10b** + **11b**.

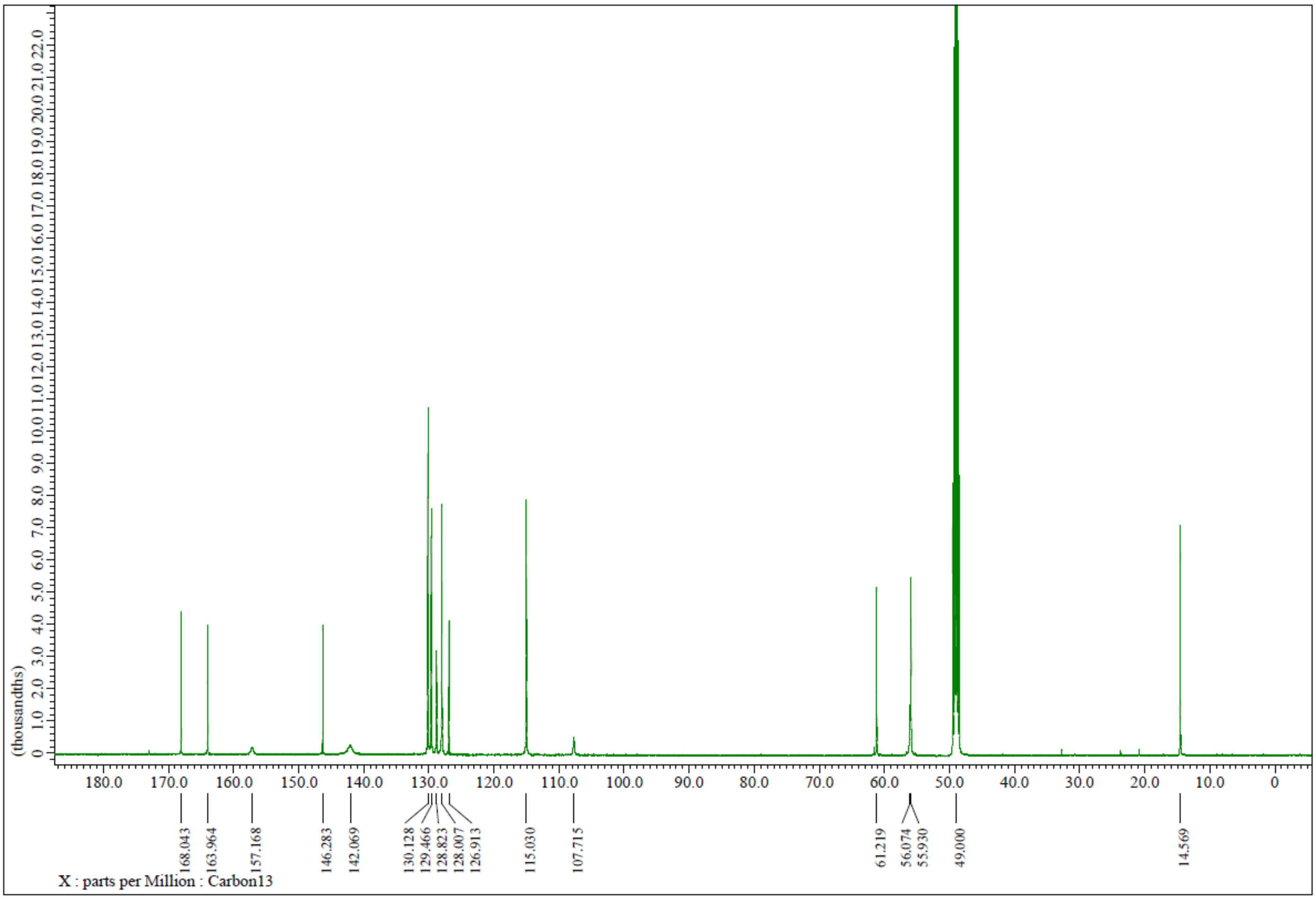

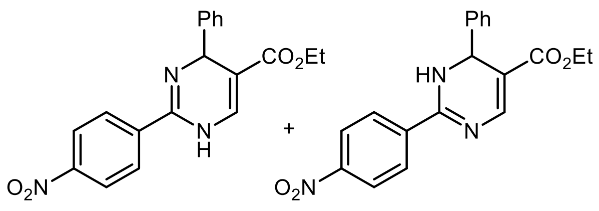


**10g** **11g**

$^{1}$H NMR (500 MHz, DMSO-$d_6$) spectrum of **10g** + **11g**.

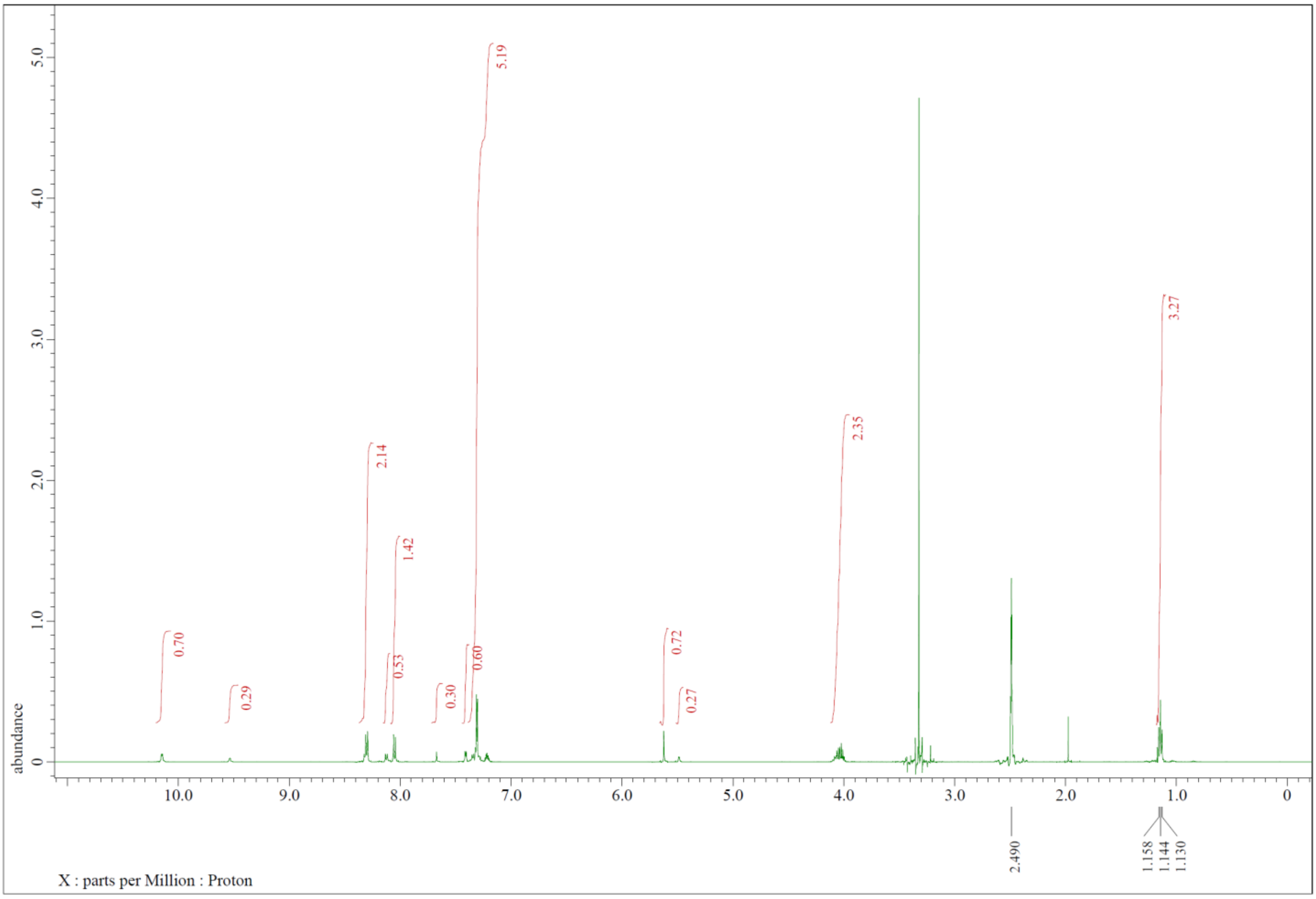

$^{1}$H NMR (500 MHz, $CD_3OD$) spectrum of **10g** + **11g**.

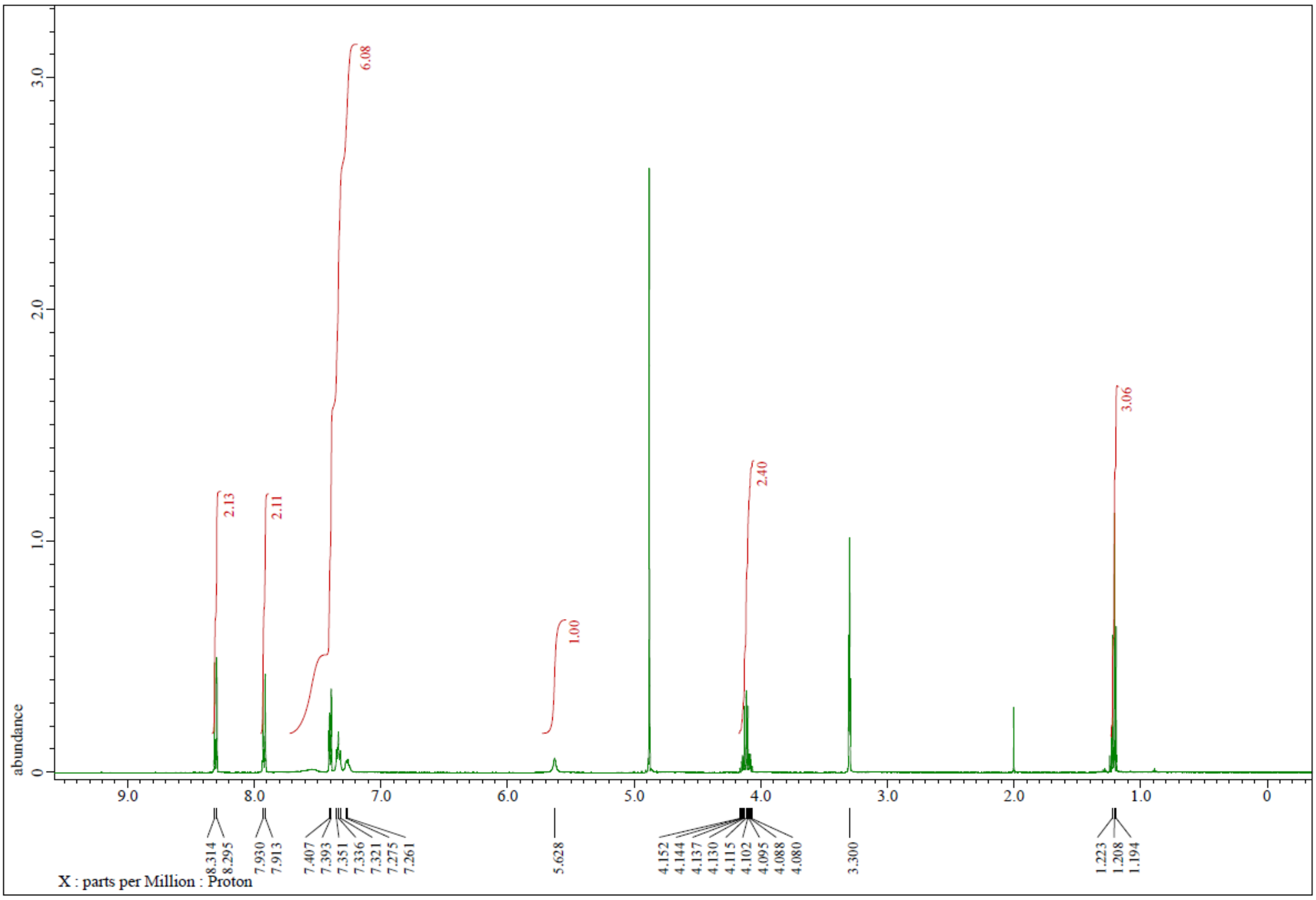

$^{13}C$ NMR (125 MHz, $CD_3OD$) spectrum of **10g** + **11g**.

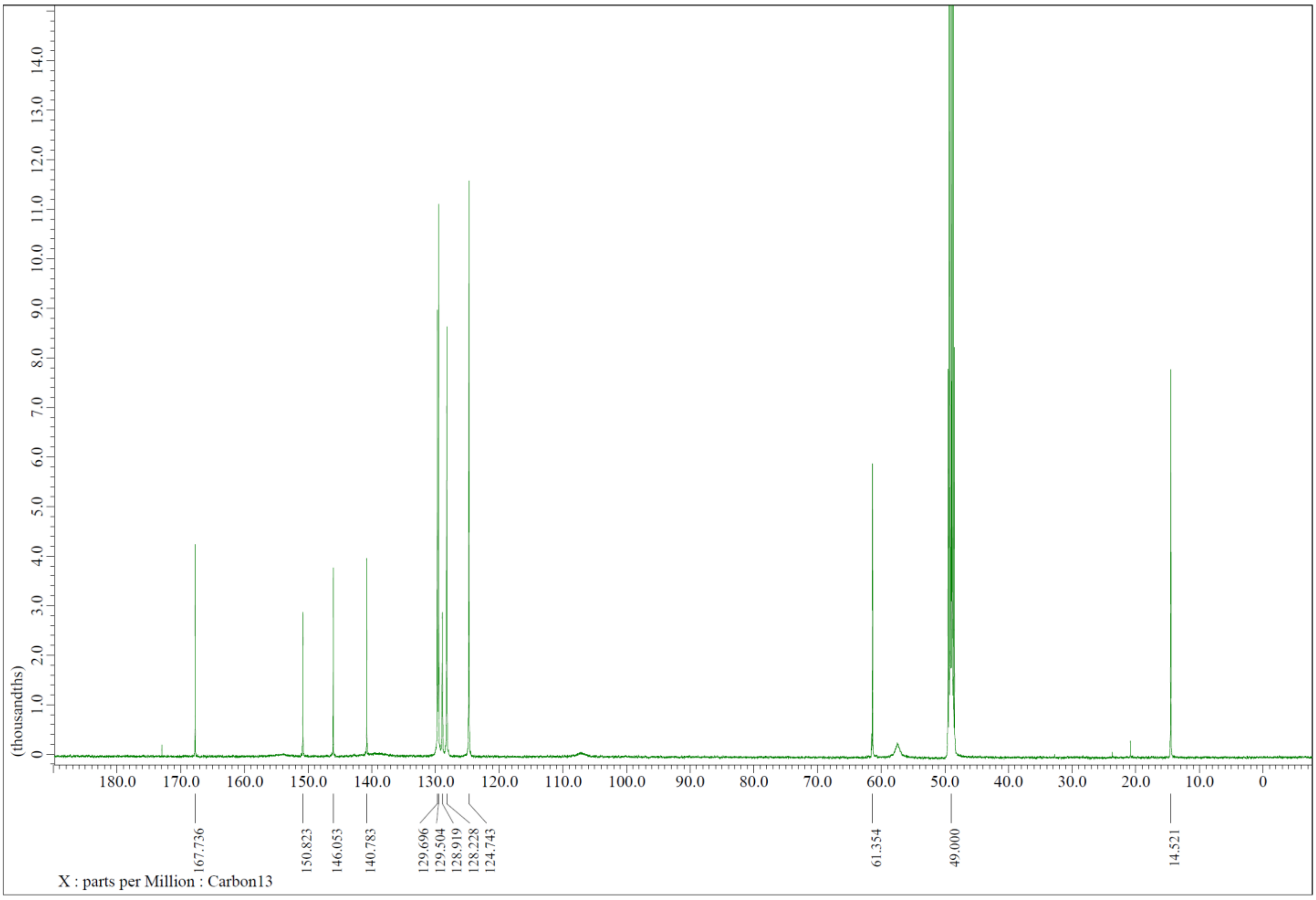

Supplement: RA-012-D2RA05155A-s001 [file RA-012-D2RA05155A-s001.pdf]
